# Clinical effectiveness of pharmacological interventions for managing chronic migraine in adults: a systematic review and network meta-analysis

**DOI:** 10.1186/s10194-023-01696-w

**Published:** 2023-12-06

**Authors:** Seyran Naghdi, Martin Underwood, Jason Madan, Anna Brown, Callum Duncan, Manjit Matharu, Aiva Aksentyte, Natasha Davies, Sophie Rees, Andrew Cooklin, Amy Grove, Hema Mistry

**Affiliations:** 1 Warwick Clinical Trials Unit, Warwick Medical School, University of Warwick, Gibbet Hill Road, Coventry, CV4 7AL UK; 2 University Hospitals Coventry and Warwickshire NHS Trust, Coventry, CV2 2DX UK; 3 Health Science Division, Warwick Medical School, University of Warwick, Coventry, CV4 7AL UK; 4 Department of Neurology, NHS Grampian, Aberdeen Royal Infirmary, Aberdeen, AB25 2ZN UK; 5 Headache and Facial Pain Group, University College London (UCL) Queen Square Institute of Neurology and The National Hospital for Neurology and Neurosurgery, London, WC1N 3BG UK; 6 Bristol Clinical Trials Unit, University of Bristol, Bristol, BS8 1QU UK

## Abstract

**Background:**

Chronic migraine can be a profoundly disabling disorder that may be treated with preventive medications. However, uncertainty remains as to which preventive medication is the most effective. We present a network meta-analysis to determine the effectiveness and rank of preventive drugs for chronic migraine in adults.

**Methods:**

We identified, reviewed, and extracted data from randomised controlled trials (RCTs) of preventive drugs for chronic migraine with at least 200 participants. Data were analysed using network meta-analysis.

**Findings:**

We included 12 RCTs of six medications (Eptinezumab, Erenumab, Fremanezumab, Galcanezumab, Onabotulinumtoxin A, and Topiramate) compared to placebo or each other. All drugs effectively reduced monthly headache and migraine days compared with placebo. The most effective drug for monthly headache days was Eptinezumab 300mg, with a mean difference of -2.46 days, 95% Credible Interval (CrI): -3.23 to -1.69. On the Surface Under the Cumulative Ranking Area (SUCRA) analysis, the probability that Eptinezumab 300mg was ranked highest was 0.82. For monthly migraine days, the most effective medication was Fremanezumab-monthly, with a mean difference: -2.77 days, 95% CrI: -3.36 to -2.17, and 0.98 probability of being ranked the highest. All included drugs, except Topiramate, improved headache-related quality of life. No eligible studies were identified for the other common preventive oral medications such as Amitriptyline, Candesartan, and Propranolol. The main reasons were that the studies did not define chronic migraine, were undertaken before the definition of chronic migraine, or were too small.

**Interpretation:**

All six medications were more effective than the placebo on monthly headache and migraine days. The absolute differences in the number of headache/migraine days are, at best, modest. No evidence was found to determine the relative effectiveness of the six included drugs with other oral preventive medications.

**Registration:**

PROSPERO (number CRD42021265990).

**Supplementary Information:**

The online version contains supplementary material available at 10.1186/s10194-023-01696-w.

## Introduction

Chronic migraine is defined as headaches on 15 days or more a month, for over three months, with features of migraine on at least eight days [1]. Chronic migraine can be a profoundly disabling condition that worsens people’s lives [2–4]. Typical estimates for its prevalence range from 1.4% to 2.2% [5]. Episodic migraine differs in that is diagnosed in people with migraine who have fewer than 15 headache days a month [1]. The overall economic costs of chronic and episodic migraine are substantial. In the USA, costs reached $36 billion in 2016; in Europe, costs reached €108 billion in 2018, and in the UK, estimates ranged from £6.2 to £9.7 billion (assuming 15%-23% of migraine prevalence in 2018) [6–8]. Most of these costs are attributable to chronic migraine; therefore, determining the most effective treatment for chronic migraine not only has the potential to benefit migraine suffers but also to reduce societal costs [9, 10].

Oral drugs have been the mainstay of chronic migraine treatment for many years. Topiramate and Propranolol are recommended by UK guideline producers—National Institute for Health and Social Care Excellence (NICE) and Scottish Intercollegiate Guidelines Network (SIGN) based on mixed-quality evidence [11, 12]. Weaker evidence supports the use of Amitriptyline, recommended by both, and for Candesartan, Flunarizine, and Valproate, recommended only by SIGN [11–13]. The definition of chronic migraine was introduced around 2007 when Topiramate was being trialled. Neither, NICE or SIGN in their guidelines or in the latest American Headache Society Consensus Statement is there a distinction between chronic and episodic migraine [11–14]. In 2012, Onabotulinumtoxin A (BTA) was approved by NICE for chronic migraine in people who had not responded to at least three prior drug treatments [15]. Since then, the new injected calcitonin gene-related peptide (CGRP) monoclonal antibodies (MAbs): Eptinezumab, Erenumab, Fremanezumab, and Galcanezumab have become available [16]. These are the first marketed drugs that have been developed specifically for the prophylaxis of migraine. Trials on other drugs have only been on episodic migraine populations, or in populations where the frequency is not clearly defined or have been a mix of the two populations.

Thus, much of the evidence used to inform treatment choices in chronic migraine, has been inferred from episodic migraine populations. Migraine frequency is also rarely described in the literature [11–13]. Whilst episodic and chronic migraine are likely to be on a continuum, with chronic migraine at the more severe end, we cannot assume that drugs shown to be effective for episodic migraine will be effective in chronic migraine.

The broad spectrum of the preventative drugs poses challenges for conventional head-to-head meta-analyses to identify the most effective option. Network meta-analysis (NMA), extends beyond the traditional pairwise meta-analysis comparison to multiple interventions providing a more precise estimate of a treatment effect size by combining both direct and indirect evidence [17]. Furthermore, they provide probabilities to rank drugs and can help guide decision-making. This systematic review and NMA was performed with the aim of describing the relative effectiveness of preventive medications for chronic migraine.

## Methods

This review is reported in accordance with PRISMA guidelines [18] and the Cochrane Handbook for Systematic Reviews of Interventions [19]. The protocol is registered in the PROSPERO database (number CRD42021265990).

### Inclusion criteria

We included randomised controlled trials (RCTs) evaluating efficacy of drugs versus placebo or other preventive drugs available in the UK for adults (18 years and over) with chronic migraine (Additional file 2: Appendix 2). We excluded studies with fewer than 100 participants per arm due to concerns around study quality.

### Search strategy and selection criteria

We constructed our initial search strategy in MEDLINE using both free text keywords and thesaurus (MeSH) terms for migraine/headache and the preventive medications, with the addition of a search filter for RCTs. No date or language limits were applied. We searched a total of seven databases. We performed forward and backward citation tracking from all included papers (Additional file 1: Appendix 1).

Title and abstract screening were conducted by two reviewers (AB/SN). We then screened according to population, intervention, comparison, and outcomes criteria (Additional file 2: Appendix 2). The abstracts of the retrieved studies were reviewed independently by two out of four reviewers (SN/AA/ND/MU). The same reviewers reviewed the full texts of the remaining studies according to the prespecified inclusion/exclusion criteria. Any discrepancies were discussed with MM/CD until resolved.

### Data extraction

Data were extracted into pre-specified Microsoft Excel forms by one reviewer and 20% randomly checked for accuracy by another reviewer. Our prespecified outcomes of interest were:

1. Monthly Headache Days (MHDs)
2. Monthly Migraine Days (MMDs)
3. Migraine Specific Quality of Life (MSQ) and the Headache Impact Test-6 (HIT-6) [20–22].

We did an additional post-hoc analysis for responder rates (≥ 50% reduction in MHDs or MMDs) to aid clinical interpretation of our findings. The limitations of responder analyses are well documented [23, 24]. Nevertheless, they may have some role in understanding the clinical importance of the findings [25].

We extracted means and standard deviations (SDs) for continuous outcomes and proportion for binary outcomes. If SDs were not provided, these were calculated from standard errors, confidence intervals, or other measures [19].

### Data analysis

For the NMA, we fitted fixed- and random-effects models with a strong prior on heterogeneity to allow for model convergence despite the limited number of studies. The posterior mean deviance as an indicator of model fit and the deviance information criterion (DIC) were used to choose between fixed and random effects models. Network plots were created for each outcome in Stata SE 17 [26] and forest plots for each drug compared to placebo as the reference treatment were generated. We assessed the overall consistency of each network by comparing the posterior mean residual deviance, DIC, and between-study SD for both the NMA model (consistency model) and the unrelated mean effects model (inconsistency model). Node splitting approach was applied to assess local consistency. The statistical analyses used a Bayesian framework using multinma package [27] in R software version 4.1.3 [28]. The ranking of each intervention was evaluated by estimating the probability that each intervention is best, second best, and so on. In addition, the treatment ranking probabilities were summarised using the Surface Under the Cumulative Ranking Area (SUCRA). The higher likelihood of therapy to be ranked top is presented by the SUCRA value closer to one [29]. Data at time points 12 or 16 weeks were analysed for all drugs, the only exception was for BTA, which had been reported at week 24.

### Assessment of risk of bias and certainty in evidence for included trials.

The risk of bias of included trials was assessed using the revised Cochrane risk-of-bias (ROB2) tool for RCTs [30] and the certainty of evidence for the NMA estimates was assessed using the GRADE framework [31].

## Results

**Fig. 1:**
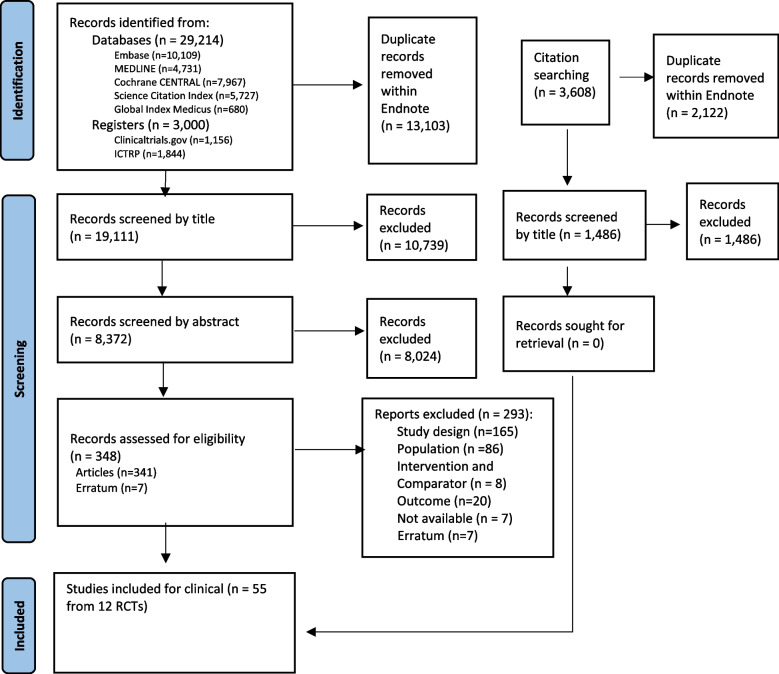
PRISMA flow diagram

We did our initial search in September 2021 (updated searches were done in November 2022 and June 2023). After the removal of duplicates we identified 19,111 records. We excluded 18,763 citations after title and abstract sifting. We obtained 348 records for full-text screening; of these, 293 were excluded. We included 55 articles reporting 12 trials [32–43] (Fig. 1). The number of articles reporting data from each trial ranged from one to 12. Excluded papers are presented in Additional file 2: Appendix 3.

### Study characteristics

Most of the included trials were performed in high income countries. All the included trials were industry funded. Sample sizes ranged from 282 to 1,130 with data from 7,909 participants. The mean age ranged from 35.7 to 46.8 years; and the percentage of females ranged from 79 to 91%. Mean MHDs and MMDs at baseline ranged from 16.2 [34] and 15.1 [44] to 22.1 [41, 43] and 20.4 [34], respectively.

**Fig. 2:**
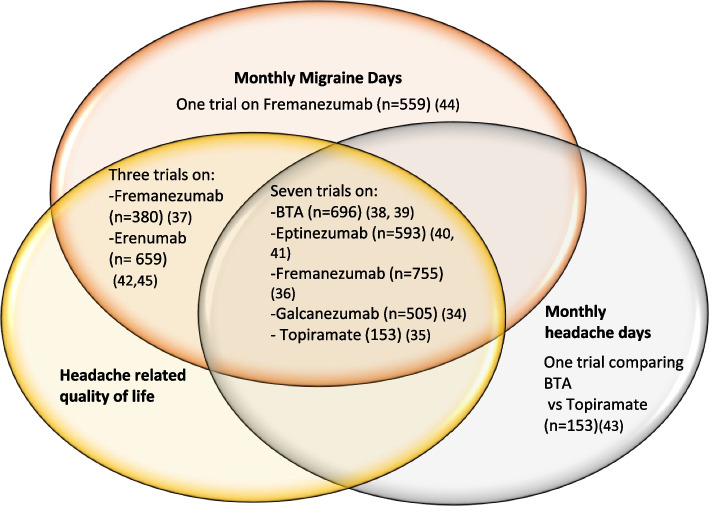
The number of included trials for three pre-specified outcomes of interest

All trials were double-blinded, except one which was an open-label trial [41]. Duration of drug treatment ranged from 12 to 36 weeks (double-blind) and 48 weeks (open-label). The included RCTs evaluated 10 different dosing regimens of CGRP MAbs and one regimen each for BTA and Topiramate. Eight trials measured their primary outcome at week 12. Baseline characteristics are summarised in Additional file 2: Appendix 4. Seven trials (*n* = 5,556) reported data on both MHDs and MMDs (Fig. 2).

**Fig. 3:**
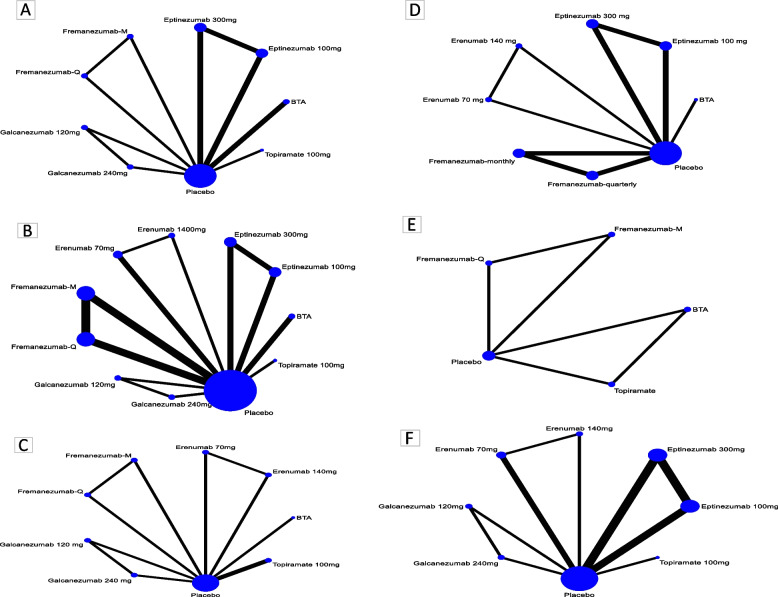
Network plots: **A** MHDs; **B** MMDs; **C** MSQ; **D** HIT-6; **E** 50% reduction in MHDs; **F** 50% reduction in MMDs

Fixed-effects NMA models were chosen because the DIC difference was less than three for all outcomes. We found no indirect evidence for most outcomes, thus the direct evidence and NMA estimates were similar (Fig. 3).

### Monthly Headache Days (MHDs) (Table 1)

MHDs were reported in eight trials (*n*= 5,838) using data on Fremanezumab, Eptinezumab, Galcanezumab, BTA, and Topiramate [32, 34, 36–39, 41, 45]. Compared with placebo, the mean difference (MD) in MHDs decreased significantly for all drugs. The most effective was Eptinezumab 300mg (MD -2.46, 95% Credible Interval (CrI) -3.23 to -1.69) and the least effective was Topiramate 100mg (MD: -1.10, 95% Crl: -2.33 to 0.17). The highest probability SUCRA ranking was 0.82 for Eptinezumab 300mg and the lowest probability SUCRA ranking was 0.31 for Topiramate 100mg.

**Table 1:**
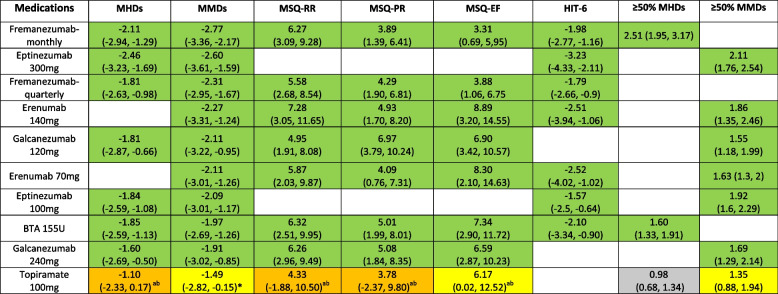
Effect size of each treatment versus Placebo (MD (95% Crl) for MHDS, MMDs, MSQ, and HIT-6, and RRs (95% Crl) for ≥ 50% MHDs and MMDs)

GRADE rating and interpretation

High:  Definitely more beneficial than Placebo

Moderate:  Probably more beneficial than Placebo;  Probably less beneficial than Placebo

Low:  May be more beneficial than Placebo

*Abbreviations*: *MHDs* Monthly Headache Days, *MMDs* Monthly Migraine Days, *MSQ-RR* Migraine-Specific QoL-Restrictive Role, *MSQ-PR* Migraine-Specific QoL-Preventative Role, *MSQ-EF* Migraine-Specific QoL-Emotional Function, *HIT-6* Headache Impact Test-6,* ≥ 50% MHDs* 50% or more response in MHDs reported in four trials (*n*= 3,039) [34, 41, 45, 46], *≥ 50% MMDs* 50% or more response in MMDs reported in seven trials (*n *= 4,695) [32, 38–40, 43–50]

^a^downgraded due to risk of bias

^b^downgraded due to imprecision

### Monthly Migraine Days (MMDs) (Table 1)

MMDs were reported in 11 trials (*n*= 8,365) using data on Fremanezumab, Eptinezumab, Galcanezumab, Erenumab, BTA, and Topiramate [32, 34–40, 42–44]. All treatments significantly reduced the mean MMDs compared to placebo. The most effective drug is Fremanezumab-monthly (MD: -2.77, 95% CrI: -3.37 to -2.16) and the least effective was Topiramate 100mg (MD: -1.49, 95% Crl: -2.82 to -0.15). Fremanezumab-monthly had the highest probability ranking to reduce MMD (SUCRA 0.98), the lowest probability ranking treatment was Topiramate 100mg (SUCRA 0.38).

### Headache-related quality of life (QoL) (Table 1)

Headache-related QoL was reported in 10 trials (*n* = 7,181). Of these, five trials used the MSQ [32, 33, 46–49] and six trials used HIT-6 [34, 35, 38, 39, 46, 49].

In each comparison using the MSQ, the drugs tested were more effective than placebo. For improvement in the MSQ-Restrictive Role (MSQ-RR), Erenumab 140mg (MD: 7.28, 95% CrI: 3.05 to 11.65, SUCRA 0.75) was superior and had the highest probability of being ranked best and Topiramate 100mg had the least improvement (MD: 4.33, 95% CrI: -1.88 to 10.5, SUCRA 0.40). For MSQ-Preventative Role (MSQ-PR), the results indicated that Galcanezumab 120mg (MD: 6.97, 95% CrI: 3.79 to 10.24, SUCRA 0.88) was more effective and had the largest SUCRA ranking. Topiramate (MD: 3.78 95% CrI: -2.37 to 9.80, SUCRA 0.44), had the least improvement, although the SUCRA ranking was slightly higher than Fremanezumab-monthly (0.44 vs. 0.41). Erenumab 140mg (MD: 8.89, 95% CrI: 3.20 to 14.55, SUCRA 0.79) was the most effective in improving of MSQ-Emotional Function (MSQ-EF) and was superior in terms of ranking; and Fremanezumab-monthly (MD: 3.31, 95% CrI: 0.69 to 5.95, SUCRA 0.23) was the least effective treatment.

The most effective and highest ranked treatment in the reduction of HIT-6 was Eptinezumab 300mg (MD: -3.22, 95% CrI: -4.33 to 2.09, SUCRA 0.98) and the least effective drug was Eptinezumab 100mg (MD: -1.56, 95% CrI: -1.87 to -0.62, SUCRA 0.45).

The global approach to test for overall consistency for all outcomes showed no evidence of inconsistency in the data points (Table 1 and Additional file 2: Appendix 5).

### Risk of bias and GRADE for included studies

Risk-of-bias ratings by trial are presented in Additional file 2: Appendix 6. In terms of overall risk of bias, two trials were rated as being at high risk of bias [41, 44], four at medium risk of bias [32, 34, 39, 40], and six at low risk of bias [35–38, 42, 43]. Overall, there was no major concern that the studies were not applicable to the research question for this assessment. Using the GRADE approach, we found that the relative certainty of the evidence for each estimate was judged to be low to high. All effect sizes (except Topiramate’s effect size) compared with placebo were assessed at high level of certainty (Table 1 and Additional file 2: Appendix 7).

## Discussion

### Overview and key findings

In this systematic review and NMA of preventive medications for chronic migraine, we found 55 papers reporting 12 RCTs [32–43]. All 12 included RCTs reported positive effects on headache days, migraine days, and headache-related QoL when compared to placebo. Eptinezumab 300mg was the most effective drug and ranked the best treatment in reduction of MHDs. For MMDs, the most effective treatment with the best rank was Fremanezumab-monthly. BTA performed better than Fremanezumab-quarterly in terms of mean change in MHDs, but not in the mean change in MMDs. In terms of a ≥ 50% reduction in MHDs, Fremanezumab monthly was superior to BTA and placebo was better than Topiramate. For a ≥ 50% reduction in MMDs, Eptinezumab 300mg was more effective than the other MAbs and Topiramate. The apparent differences in relative effectoveness of different prepraration on monthly migraine days and monthly headache days is most probably due to the differences in the studies included in the two analyses (Fig. 2). Overall, the CGRP MAbs are marginally more effective than BTA with no consistent pattern as to which MAbs might overall be the most effective, and Topiramate was the clear outlier.

The largest improvement on headache-related QoL was related to Galcanezumab 120 mg for MSQ-PR, but for MSQ-RR and MSQ-EF Erenumab 140 mg was superior to other drugs. For HIT-6, the results depict that Eptinezumab 300 mg was the most effective treatment. Strikingly, the least effective drug was Topiramate with a ≤ 0.3 probability of being the most effective for MHDs and MMDs with only very limited evidence for an effect on headache-related QoL.

It should be noted that there are no established minimally clinically important differences for monthly headache and migraine days [51]. To set our findings in context, we also did some analysis on trials reporting 50% reductions in headache/migraine days (Additional file 2: Appendix 5). In the placebo groups for headache days and migraine days, ~ 25% and ~ 30% of participants respectively, improved by at least 50%. Using data from the original publications, for headache days, Fremanezumab-monthly increased the proportion responding by at least 2.5 times which translates into a number needed to treat (NNT) of around 4.4 [34]. Whilst for migraine days for CGRP MAbs the NNT ranged between 1.55 and 2.11 times (NNT for Galcanezumab 120mg ~ 9.9 [32] and for Eptinezumab 300mg ranged from 3.6 [50] to 6.1 [39]) and for Topiramate this was 1.35 times (NNT ~ 13.9 [45]).

### Generalisibilty and other studies

Our findings for MHDs and MMDs are largely in line with the 2021 NMA, that only included trials of anti-CGRP MAbs. They included seven RCTs (*N* = 5,164) on people with chronic migraine [52]. They concluded Fremanezumab-monthly and Eptinezumab 300mg are effective therapies with an acceptable safety profile for managing chronic migraine [52]. We have expanded on this study by including an additional trial of CGRP MAbs and four trials of BTA and Topiramate [36, 37, 41, 43, 44]; furthermore we also looked at headache-related QoL.

In another study, Erenumab was more effective than BTA in the reduction of MMDs, which is also in line with our results [53]. The effectiveness of different CGRP MAbs for patients who failed previous treatments was investigated [54]. The results showed that Galcanezumab 240mg was the most effective in reducing MMDs followed by Fremanezumab-monthly and Eptinezumab 300mg [54]. This discrepancy with our finding, may be due to previous treatment failures for this population. Moreover, Erenumab in our finding was ranked as the third-best treatment in reduction of MMDs, whilst ranked as the last treatment for those participants with previous treatment failures.

The effect of Eptinezumab 300mg on the HIT-6 (MD: 3.22, 95% CrI: 2.09 to 3.59) well exceeded the target difference of 2.0 set for a 2022 trial of supportive self-management for people living with chronic headaches [55]. The effect sizes observed for commonly used dose of CGRP-MAbs with HIT-6 data are similar, indicating that they too are likely to have a worthwhile effect.

### Strengths

The main strength of our study is the analysis is of adequately powered studies of the newer medications which were trialled after introducing the concept of chronic migraine in 2007. This included the CGRP MAbs namely Fremanezumab, Eptinezumab, Galcanezumab, and Erenumab, along with BTA and Topiramate. Another strength of this review is the comprehensiveness of the search strategy used. The search was run on a broad range of electronic databases to identify all relevant trials and had no date or language restrictions. Furthermore, as migraine is one of the most common disorders causing disability [56] and the core outcome set for preventive trials in migraine gives equal weight to headache/migraine days and headache-related QoL [57], we evaluated how the drugs affected QoL and disabilities associated with migraine.

### Limitations

All included studies were industry funded; therefore, some caution is needed when interpreting the results. In addition, all trials were placebo-controlled; thus, we were not able to estimate any indirect comparisons and assess the local inconsistency. This means that there were no direct drug-to-drug comparisons in our included trials. We included a trial which involved participants with a history of failure of up to four migraine preventive drug classes [42] which might have resulted in bias in our results. Most trials included participants with, and without medication overuse. It is unclear how this might have affected our conclusions.

Excluding studies with fewer than 100 participants per arm has limited our analyses to more recently investigated treatments where the trial methodology is more precise, at the risk that we might exclude pertinent data from smaller, usually older, trials. Because of this, we were unable to identify any eligible studies of adequate quality for other oral drugs commonly used in the management of chronic migraine, such as Amitriptyline, Candesartan, Flunarizine and Propranolol. We re-checked our excluded studies table to check for any studies of currently recommended treatments by NICE or SIGN to identify studies only excluded due to sample size criterion. We found one trial (*n* = 191) comparing Topiramate and Placebo to Topiramate and Propranolol, which nearly fits our criteria [58]. We would not have added this study to our NMA, as they did not report differences in headache days or headache-related QoL at three or six months. There may be other relevant data from trials with mixed populations, or where entry criteria are inadequately defined. Whilst these studies may have included people with chronic migraine, the heterogenous nature of these populations makes them inappropriate to include here.

The selection of ‘migraine days’ as the primary outcome measure and headache-related QoL as a secondary outcome in our study offers a comprehensive view of migraine’s impact on patients’ daily lives, reflecting its real-world complexity. However, it’s important to acknowledge that this choice may limit direct comparability with older studies using ‘attack frequency’ as an outcome measure.

Our results are subject to the quality of the included studies. Approximately 50% of the included RCTs had low risk of bias, but 33% had some concerns of bias. In general, the certainty of evidence for each NMA estimate was judged to be low to high, which highlighted the relative robustness of our findings for application in clinical settings.

Finally, chronic migraine was introduced as a concept in 2007 and so all earlier studies of preventative medications have not been trialled under the definition of ‘chronic migraine’. On the other hand, most small old trials on oral migraine preventatives are of poor methodological quality and/or underpowered and including them in the NMA would have resulted in a large degree of heterogeneity resulting in a high risk of bias. Therefore, our NMA analyses was restricted to BTA, CGRP MAbs and Topiramate. Further high-quality trials on oral drugs such as Amitriptyline, Candesartan, Flunarizine, and Propranolol that are recommended by NICE and/or SIGN are needed.

## Conclusion

To the best of our knowledge, our study is the most comprehensive NMA of preventive medications for adults with chronic migraine. Overall, our data confirm that several drugs provide a worthwhile benefit for people with chronic migraine leading to improvements in headache-related QoL and reduction in headache/migraine days. Our findings show that overall CGRP-MAbs are more effective than Topiramate, but only marginally effective compared to BTA. We are unable to say anything about the comparative effectiveness of the six included drugs and other drugs commonly used in chronic migraine such as Propranolol, Amitriptyline, Candesartan and Flunarizine.

### Supplementary Information

**Additional file 1: Appendix 1.** Literature searches.

**Additional file 2: Appendix 2.** Inclusion and exclusion criteria. **Appendix 3.** The list of excluded studies. **Appendix 4.** Baseline characteristics of the 11 RCTs presented in 51 included studies- part 1. Baseline characteristics of the 12 RCTs presented in 55 included studies- part 2. **Appendix 5.** Network meta-analysis results. **Appendix 6.** Risk of bias assessment result. **Appendix 7.** The Grading of Recommendations Assessment, Development and Evaluation (GRADE) approach for rating the quality of estimates of treatment effect size.

## Data Availability

The datasets used and/or used during the current study are available from the corresponding author upon reasonable request.
