# Supplementary material for: Clinical effectiveness of pharmacological interventions for managing chronic migraine in adults: a systematic review and network meta-analysis
Source: J Headache Pain. 2023 Dec 6;24(1):164. doi: 10.1186/s10194-023-01696-w (PMC10702068; doi:10.1186/s10194-023-01696-w)
Supplement: Supplementary file 1 — Additional file 1: Appendix 1. Literature searches. [file 10194_2023_1696_MOESM1_ESM.docx]

**Additional file 1: Appendix 1. Literature searches**

## Overview

| ***Bibliographic databases and clinical trials registers*** | | | |
| --- | --- | --- | --- |
| **Database** | | **Date searched** | **Number of records** |
| MEDLINE All (via Ovid) | | 08/09/21 | 4,029 |
| Embase (via Ovid) | | 08/09/21 | 8,404 |
| Cochrane CENTRAL (via Cochrane Library) | | 08/09/21 | 6,754 |
| Science Citation Index (via Web of Science) | | 08/09/21 | 4,737 |
| Global Index Medicus (via World Health Organization) | | 14/09/21 | 200 |
| Clinicaltrials.gov | | 15/09/21 | 338 |
| International Clinical Trials Registry Platform (ICTRP) (World Health Organization) | | 15/09/21 | 512 |
| **Total number of records retrieved: 24,974**  **Duplicates removed (EndNote): 8,368**  **Final number for screening: 16,606** | | | |
| ***Bibliographic databases and clinical trials registers; additional search for riboflavin, magnesium and coenzyme Q10*** | | | |
| **Source** | | **Date searched** | **Number of records** |
| MEDLINE All (via Ovid) | | 08/02/22 | 163 |
| Embase (via Ovid) | | 08/02/22 | 587 |
| Cochrane CENTRAL (via Cochrane Library) | | 08/02/22 | 331 |
| Science Citation Index (via Web of Science) | | 08/02/22 | 359 |
| Global Index Medicus (via World Health Organization) | | 08/02/22 | 24 |
| Clinicaltrials.gov | | 08/02/22 | 15 |
| International Clinical Trials Registry Platform (ICTRP) (World Health Organization) | | 08/02/22 | 38 |
| **Total number of records retrieved: 1,517**  **Duplicates removed within this set (EndNote): 481**  **Duplicates removed against original search (EndNote):  448**  **Final number for screening: 588** | | | |
| ***Pragmatic search for recent systematic reviews, to check reference lists/included studies*** | | | |
| **Database** | | **Date searched** | **Number of records** |
| MEDLINE All (via Ovid) | | 14/02/22 | 114 |
| Embase (via Ovid) | | 14/02/22 | 164 |
| Cochrane Database of Systematic Reviews (via Cochrane Library) | | 14/02/22 | 4 |
| **Total number of records retrieved: 282**  **Duplicates removed within this set (EndNote): 103**  **Final number for screening: 179** | | | |
| ***Bibliographic databases and clinical trials registers; search update November 2022 (including all relevant drug terms)*** | | | |
| **Database** | **Date searched** | | **Number of records** |
| MEDLINE All (via Ovid) | 07/11/22 | | 390 |
| Embase (via Ovid) | 07/11/22 | | 710 |
| Cochrane CENTRAL (via Cochrane Library) | 07/11/22 | | 713 |
| Science Citation Index (via Web of Science) | 07/11/22 | | 440 |
| Global Index Medicus (via World Health Organization) | 07/11/22 | | 222 |
| Clinicaltrials.gov | 08/11/22 | | 390 |
| International Clinical Trials Registry Platform (ICTRP) (World Health Organization) | 08/11/22 | | 631 |
| **Total number of records retrieved: 3,496**  **Duplicates removed within this set (EndNote): 1,096**  **Duplicates removed against previous searches (EndNote): 1,066**  **Final number for screening: 1,334** | | | |

| ***Other sources; citation tracking*** | | |
| --- | --- | --- |
| **Source** | **Date searched** | **Number of records** |
| Reference lists – included studies (Web of Science) | 23/11/22 | 875 |
| Forwards citation tracking:  Science Citation Index (Web of Science) | 22-23/11/22 | 2,710 |
| Forwards citation tracking: Google Scholar (for studies not found in Web of Science only) | 23/11/22 | 23 |
| **Total number of records retrieved: 3,608**  **Duplicates removed (both within this set and against previous searches) (Endnote): 2,122**  **Final number for screening: 1,486** | | |
| ***Checking for retraction notices, errata and comments relating to included studies*** | | |
| **Source** | **Date searched** | **Number of records** |
| MEDLINE All (via Ovid) | 22/11/22 | 23 |
| Embase (via Ovid) | 22/11/22 | 0 |
| Retraction Watch website | 22/11/22 | 0 |
| **Total number of records retrieved: 23** | | |

| ***Bibliographic databases and clinical trials registers; search update June 2023 (including all relevant drug terms)*** | | |
| --- | --- | --- |
| **Database** | **Date searched** | **Number of records** |
| MEDLINE All (via Ovid) | 15/06/23 | 149 |
| Embase (via Ovid) | 15/06/23 | 408 |
| Cochrane CENTRAL (via Cochrane Library) | 15/06/23 | 169 |
| Science Citation Index (via Web of Science) | 15/06/23 | 191 |
| Global Index Medicus (via World Health Organization) | 15/06/23 | 234 |
| Clinicaltrials.gov | 15/06/23 | 413 |
| International Clinical Trials Registry Platform (ICTRP) (World Health Organization) | 15/06/23 | 663 |
| **Total number of records retrieved: 2,227**  **Duplicates removed (both within this set and against previous searches) (EndNote): 1,644**  **Final number for screening: 583** | | |

## MEDLINE search strategy: original searches, September 2021

Date searched: 08/09/21

Database: Ovid MEDLINE(R) ALL <1946 to September 07, 2021>

Search Strategy:

--------------------------------------------------------------------------------

1 (headache* or head ache* or migrain* or cephalgi* or cephalalgi* or hemicrani*).ab,kf,ti. (112921)

2 Headache/ or exp Headache Disorders/ (61239)

3 1 or 2 [population: migraine/headache] (124144)

4 (((calcitonin gene-related peptide or CGRP) adj5 (antibod* or antagon* or inhibit* or block*)) or anti-CGRP or anti-calcitonin gene-related peptide or monoclonal antibod* or mAb or mAbs or moAb or moAbs).ab,kf,ti. (216437)

5 Calcitonin Gene-Related Peptide/ai (436)

6 Antibodies, Monoclonal/ or Antibodies, Monoclonal, Humanized/ (217039)

7 Calcitonin Gene-Related Peptide Receptor Antagonists/ (701)

8 (erenumab or galcanezumab or fremanezumab or eptinezumab).ab,kf,ti,nm. (507)

9 (rimegepant or ubrogepant or atogepant or gepant?).ab,kf,ti,nm. (214)

10 exp Botulinum Toxins/ (17105)

11 (botulin* adj toxin*).ab,kf,ti,nm. (21943)

12 (botulinum* or botox* or onabotulinum*).ab,kf,ti,nm. (25159)

13 (antidepress* or anti depress*).ab,kf,ti. (73890)

14 exp Antidepressive Agents/ (153122)

15 (amitriptyline or venlafaxine or mirtazapine or duloxetine).ab,kf,ti,nm. (17955)

16 exp "Serotonin and Noradrenaline Reuptake Inhibitors"/ (5005)

17 (SNRI or SNRIs or (serotonin adj2 (noradrenaline or norepinephrine) adj reuptake inhib*)).ab,kf,ti. (2908)

18 exp Angiotensin-Converting Enzyme Inhibitors/ (45324)

19 (Angiotensin Converting Enzyme Inhibit* or ACE inhibit*).ab,kf,ti. (37937)

20 acei.ab,kf,ti. (4344)

21 lisinopril.ab,kf,ti,nm. (3086)

22 ((angiotensin receptor or angiotensin II receptor) adj (block* or antagon*)).ab,kf,ti. (14474)

23 (ARB or ARBs).ab,kf,ti. (7873)

24 exp Angiotensin Receptor Antagonists/ (25403)

25 candesartan.ab,kf,ti,nm. (3374)

26 ((beta adj3 block*) or betablock*).ab,kf,ti. (55697)

27 ((adrenergic or adrenoreceptor* or adrenoceptor*) adj3 (antagon* or block*)).ab,kf,ti. (34997)

28 exp Adrenergic beta-Antagonists/ (85444)

29 (propranolol or metoprolol or timolol or atenolol or nadolol or nebivolol or pindolol).ab,kf,ti,nm. (67114)

30 (calcium adj2 (block* or antagon* or inhibit*)).ab,kf,ti. (41676)

31 (CCB or CCBs).ab,kf,ti. (2619)

32 exp Calcium Channel Blockers/ (88532)

33 (flunarizine or verapamil).ab,kf,ti,nm. (27700)

34 (anticonvuls* or antiepilep* or anti convuls* or anti epilep*).ab,kf,ti. (53599)

35 exp Anticonvulsants/ (147158)

36 (topiramate or valproate or divalproex or valproic acid or gabapentin).ab,kf,ti,nm. (31200)

37 Pizotyline/ (250)

38 (pizotifen or pizotyline).ab,kf,ti,nm. (418)

39 (alpha adj4 agonist*).ab,kf,ti. (15369)

40 exp Adrenergic alpha-Agonists/ (164069)

41 (clonidine or guanfacine).ab,kf,ti,nm. (19180)

42 4 or 5 or 6 or 7 or 8 or 9 or 10 or 11 or 12 or 13 or 14 or 15 or 16 or 17 or 18 or 19 or 20 or 21 or 22 or 23 or 24 or 25 or 26 or 27 or 28 or 29 or 30 or 31 or 32 or 33 or 34 or 35 or 36 or 37 or 38 or 39 or 40 or 41 [Interventions: named drugs/drug classes or types] (1098623)

43 randomized controlled trial.pt. (542809)

44 controlled clinical trial.pt. (94373)

45 randomized.ab. (533045)

46 placebo.ab. (221237)

47 clinical trials as topic.sh. (197235)

48 randomly.ab. (365421)

49 trial.ti. (247114)

50 43 or 44 or 45 or 46 or 47 or 48 or 49 (1392358)

51 exp animals/ not humans.sh. (4882975)

52 50 not 51 [RCTs filter] (1281368)

53 3 and 42 and 52 [population and interventions and RCTs filter] (3949)

54 ("in data review" or in process or publisher or "pubmed not medline").st. (4677722)

55 (random* or controlled trial* or clinical trial* or rct).ab,kf,ti. (1547833)

56 54 and 55 [pragmatic filter to pick up RCTs that have not been fully indexed for MEDLINE yet] (236445)

57 3 and 42 and 56 [population and interventions and non-MEDLINE RCT filter] (365)

58 53 or 57 (4029)

The migraine/headache search terms (lines 1-3) and botox search terms (lines 10-12) are based on those used in:

Herd CP, Tomlinson CL, Rick C, Scotton WJ, Edwards J, Ives N, Clarke CE, Sinclair A. Botulinum toxins for the prevention of migraine in adults. Cochrane Database of Systematic Reviews 2018, Issue 6. Art. No.: CD011616. DOI: 10.1002/14651858.CD011616.pub2.

The search filter for RCTs (lines 43-52) is the Cochrane Highly Sensitive Search Strategy for identifying randomized trials in MEDLINE: sensitivity- and precision-maximizing version (2008 revision); Ovid format:

Lefebvre C, Glanville J, Briscoe S, Littlewood A, Marshall C, Metzendorf M-I, et al. Technical Supplement to Chapter 4: Searching for and selecting studies. In: Higgins JPT, Thomas J, Chandler J, Cumpston MS, Li T, Page MJ, Welch VA (eds). Cochrane Handbook for Systematic Reviews of Interventions Version 6.2 (updated February 2021). Cochrane, 2021. Available from: [www.training.cochrane.org/handbook](http://www.training.cochrane.org/handbook).

## MEDLINE search strategy: additional searches for riboflavin, magnesium and coenzyme Q10, February 2022

Date searched: 08/02/22

Ovid MEDLINE(R) ALL <1946 to February 07, 2022>

1 (headache* or head ache* or migrain* or cephalgi* or cephalalgi* or hemicrani*).ab,kf,ti. 115846

2 Headache/ or exp Headache Disorders/ 62888

3 1 or 2 [population: migraine/headache] 127140

4 Riboflavin/ 9019

5 (riboflavin or vitamin b2 or vitamin b 2).ab,kf,ti,nm. 14667

6 Ubiquinone/ 9986

7 (coenzyme q* or co enzyme q* or ubidecarenone or ubiquino* or coq10 or co q10).ab,kf,ti,nm. 17133

8 Magnesium/ or exp Magnesium Compounds/ 83822

9 magnesium.ab,kf,ti,nm. 113129

10 4 or 5 or 6 or 7 or 8 or 9 [interventions: 3 drugs added February 2022] 147736

11 randomized controlled trial.pt. 558117

12 controlled clinical trial.pt. 94685

13 randomized.ab. 550007

14 placebo.ab. 225467

15 clinical trials as topic.sh. 199113

16 randomly.ab. 375668

17 trial.ti. 256318

18 11 or 12 or 13 or 14 or 15 or 16 or 17 1425517

19 exp animals/ not humans.sh. 4955382

20 18 not 19 [Cochrane Highly Sensitive Search Strategy for identifying randomized trials in MEDLINE: sensitivity- and precision-maximizing version (2008 revision)] 1311348

21 3 and 10 and 20 [population + interventions + RCT filter] 161

22 ("in data review" or in process or publisher or "pubmed not medline").st. 4673502

23 (random* or controlled trial* or clinical trial* or rct).ab,kf,ti. 1597122

24 22 and 23 [filter to pick up RCTs that have not been fully indexed for MEDLINE yet] 231267

25 3 and 10 and 24 [population + interventions + RCT filter for non indexed studies] 18

26 21 or 25 163

## MEDLINE search strategy: pragmatic search for recent systematic reviews, to check reference lists/included studies, February 2022

Date searched: 14/02/22

Ovid MEDLINE(R) ALL <1946 to February 11, 2022>

 1 exp Migraine Disorders/pc 2569

2 "migrain*".ab,hw,kf,ti. 43508

3 ((prevent* or prophyla*) adj2 (treatment? or therap* or medication? or drug?)).ab,hw,kf,ti. 179039

4 2 and 3 3218

5 (migrain* adj4 (prevent* or prophyla*)).ab,hw,kf,ti. 3883

6 1 or 4 or 5 5846

7 (metaanalys* or "meta analys*").tw. 222321

8 (systematic* adj3 review*).mp. 276043

9 meta analysis.pt. 152804

10 7 or 8 or 9 [pragmatic systematic review filter] 392108

11 (((calcitonin gene-related peptide or CGRP) adj5 (antibod* or antagon* or inhibit* or block*)) or anti-CGRP or anti-calcitonin gene-related peptide or monoclonal antibod* or mAb or mAbs or moAb or moAbs).ab,kf,ti. 219332

12 Calcitonin Gene-Related Peptide/ai 452

13 Antibodies, Monoclonal/ or Antibodies, Monoclonal, Humanized/ 221635

14 Calcitonin Gene-Related Peptide Receptor Antagonists/ 781

15 (erenumab or galcanezumab or fremanezumab or eptinezumab).ab,kf,ti,nm. 588

16 (rimegepant or ubrogepant or atogepant or gepant?).ab,kf,ti,nm. 247

17 exp Botulinum Toxins/ 17563

18 (botulin* adj toxin*).ab,kf,ti,nm. 22444

19 (botulinum* or botox* or onabotulinum*).ab,kf,ti,nm. 25677

20 (antidepress* or anti depress*).ab,kf,ti. 75518

21 exp Antidepressive Agents/ 155320

22 (amitriptyline or venlafaxine or mirtazapine or duloxetine).ab,kf,ti,nm. 18204

23 exp "Serotonin and Noradrenaline Reuptake Inhibitors"/ 5141

24 (SNRI or SNRIs or (serotonin adj2 (noradrenaline or norepinephrine) adj reuptake inhib*)).ab,kf,ti. 2996

25 exp Angiotensin-Converting Enzyme Inhibitors/ 45974

26 (Angiotensin Converting Enzyme Inhibit* or ACE inhibit*).ab,kf,ti. 38458

27 acei.ab,kf,ti. 4519

28 lisinopril.ab,kf,ti,nm. 3114

29 ((angiotensin receptor or angiotensin II receptor) adj (block* or antagon*)).ab,kf,ti. 14830

30 (ARB or ARBs).ab,kf,ti. 8220

31 exp Angiotensin Receptor Antagonists/ 26157

32 candesartan.ab,kf,ti,nm. 3407

33 ((beta adj3 block*) or betablock*).ab,kf,ti. 56350

34 ((adrenergic or adrenoreceptor* or adrenoceptor*) adj3 (antagon* or block*)).ab,kf,ti. 35141

35 exp Adrenergic beta-Antagonists/ 85957

36 (propranolol or metoprolol or timolol or atenolol or nadolol or nebivolol or pindolol).ab,kf,ti,nm. 67483

37 (calcium adj2 (block* or antagon* or inhibit*)).ab,kf,ti. 41979

38 (CCB or CCBs).ab,kf,ti. 2692

39 exp Calcium Channel Blockers/ 89276

40 (flunarizine or verapamil).ab,kf,ti,nm. 27822

41 (anticonvuls* or antiepilep* or anti convuls* or anti epilep*).ab,kf,ti. 54399

42 exp Anticonvulsants/ 149062

43 (topiramate or valproate or divalproex or valproic acid or gabapentin).ab,kf,ti,nm. 31789

44 Pizotyline/ 250

45 (pizotifen or pizotyline).ab,kf,ti,nm. 420

46 (alpha adj4 agonist*).ab,kf,ti. 15482

47 exp Adrenergic alpha-Agonists/ 165206

48 (clonidine or guanfacine).ab,kf,ti,nm. 19260

49 Riboflavin/ 9020

50 (riboflavin or vitamin b2 or vitamin b 2).ab,kf,ti,nm. 14670

51 Ubiquinone/ 9995

52 (coenzyme q* or co enzyme q* or ubidecarenone or ubiquino* or coq10 or co q10).ab,kf,ti,nm. 17147

53 Magnesium/ or exp Magnesium Compounds/ 83845

54 magnesium.ab,kf,ti,nm. 113174

55 or/11-54 1249348

56 6 and 10 and 55 182

57 limit 56 to yr="2017 - 2022" 114

## MEDLINE search strategy: update searches, November 2022 & June 2023

Date searched: 07/11/22

Ovid MEDLINE(R) ALL <1946 to November 04, 2022>

1 (headache* or head ache* or migrain* or cephalgi* or cephalalgi* or hemicrani*).ab,kf,ti. 121076

2 Headache/ or exp Headache Disorders/ 64821

3 1 or 2 [population: migraine/headache, based on Cochrane botox review] 132425

4 (((calcitonin gene-related peptide or CGRP) adj5 (antibod* or antagon* or inhibit* or block*)) or anti-CGRP or anti-calcitonin gene-related peptide or monoclonal antibod* or mAb or mAbs or moAb or moAbs).ab,kf,ti. 224346

5 Calcitonin Gene-Related Peptide/ai 463

6 Antibodies, Monoclonal/ or Antibodies, Monoclonal, Humanized/ 227720

7 Calcitonin Gene-Related Peptide Receptor Antagonists/ 887

8 (erenumab or galcanezumab or fremanezumab or eptinezumab).ab,kf,ti,nm. 730

9 (rimegepant or ubrogepant or atogepant or gepant?).ab,kf,ti,nm. 300

10 exp Botulinum Toxins/ 18153

11 (botulin* adj toxin*).ab,kf,ti,nm. 23232

12 (botulinum* or botox* or onabotulinum*).ab,kf,ti,nm. 26565

13 (antidepress* or anti depress*).ab,kf,ti. 78168

14 exp Antidepressive Agents/ 158352

15 (amitriptyline or venlafaxine or mirtazapine or duloxetine).ab,kf,ti,nm. 18641

16 exp "Serotonin and Noradrenaline Reuptake Inhibitors"/ 5336

17 (SNRI or SNRIs or (serotonin adj2 (noradrenaline or norepinephrine) adj reuptake inhib*)).ab,kf,ti. 3138

18 exp Angiotensin-Converting Enzyme Inhibitors/ 46764

19 (Angiotensin Converting Enzyme Inhibit* or ACE inhibit*).ab,kf,ti. 39244

20 acei.ab,kf,ti. 4749

21 lisinopril.ab,kf,ti,nm. 3155

22 ((angiotensin receptor or angiotensin II receptor) adj (block* or antagon*)).ab,kf,ti. 15370

23 (ARB or ARBs).ab,kf,ti. 8687

24 exp Angiotensin Receptor Antagonists/ 27181

25 candesartan.ab,kf,ti,nm. 3449

26 ((beta adj3 block*) or betablock*).ab,kf,ti. 57470

27 ((adrenergic or adrenoreceptor* or adrenoceptor*) adj3 (antagon* or block*)).ab,kf,ti. 35378

28 exp Adrenergic beta-Antagonists/ 86663

29 (propranolol or metoprolol or timolol or atenolol or nadolol or nebivolol or pindolol).ab,kf,ti,nm. 68123

30 (calcium adj2 (block* or antagon* or inhibit*)).ab,kf,ti. 42541

31 (CCB or CCBs).ab,kf,ti. 2828

32 exp Calcium Channel Blockers/ 90326

33 (flunarizine or verapamil).ab,kf,ti,nm. 28045

34 (anticonvuls* or antiepilep* or anti convuls* or anti epilep*).ab,kf,ti. 55690

35 exp Anticonvulsants/ 152010

36 (topiramate or valproate or divalproex or valproic acid or gabapentin).ab,kf,ti,nm. 32842

37 Pizotyline/ 252

38 (pizotifen or pizotyline).ab,kf,ti,nm. 425

39 (alpha adj4 agonist*).ab,kf,ti. 15644

40 exp Adrenergic alpha-Agonists/ 166795

41 (clonidine or guanfacine).ab,kf,ti,nm. 19418

42 Riboflavin/ 9260

43 (riboflavin or vitamin b2 or vitamin b 2).ab,kf,ti,nm. 15160

44 Ubiquinone/ 10256

45 (coenzyme q* or co enzyme q* or ubidecarenone or ubiquino* or coq10 or co q10).ab,kf,ti,nm. 17694

46 Magnesium/ or exp Magnesium Compounds/ 85028

47 magnesium.ab,kf,ti,nm. 115926

48 4 or 5 or 6 or 7 or 8 or 9 or 10 or 11 or 12 or 13 or 14 or 15 or 16 or 17 or 18 or 19 or 20 or 21 or 22 or 23 or 24 or 25 or 26 or 27 or 28 or 29 or 30 or 31 or 32 or 33 or 34 or 35 or 36 or 37 or 38 or 39 or 40 or 41 or 42 or 43 or 44 or 45 or 46 or 47 [Interventions: named drugs/drug classes or types] 1275840

49 randomized controlled trial.pt. 579949

50 controlled clinical trial.pt. 95083

51 randomized.ab. 580977

52 placebo.ab. 232922

53 clinical trials as topic.sh. 200534

54 randomly.ab. 394586

55 trial.ti. 273031

56 49 or 50 or 51 or 52 or 53 or 54 or 55 1482588

57 exp animals/ not humans.sh. 5060853

58 56 not 57 [Cochrane Highly Sensitive Search Strategy for identifying randomized trials in MEDLINE: sensitivity- and precision-maximizing version (2008 revision)] 1364006

59 3 and 48 and 58 [population and interventions and RCT filter] 4313

60 ("in data review" or in process or publisher or "pubmed not medline").st. 4897386

61 (random* or controlled trial* or clinical trial* or rct).ab,kf,ti. 1688331

62 60 and 61 [filter to pick up RCTs that have not been fully indexed for MEDLINE yet] 242577

63 3 and 48 and 62 [population and interventions and non-MEDLINE RCT filter] 328

64 59 or 63 4390

65 limit 64 to ed=20210908-20221107 303

66 limit 64 to ep=20210908-20221107 211

67 limit 64 to dt=20210908-20221107 259

68 limit 64 to ez=20210908-20221107 259

69 limit 64 to da=20210908-20221107 366

70 65 or 66 or 67 or 68 or 69 390

Date searched: 15/06/23

Ovid MEDLINE(R) ALL <1946 to June 14, 2023>

*As above, but lines 64-70 are:*

64 59 or 63 4509

65 limit 64 to ed=20221107-20230615 101

66 limit 64 to ep=20221107-20230615 98

67 limit 64 to dt=20221107-20230615 127

68 limit 64 to ez=20221107-20230615 127

69 limit 64 to da=20221107-20230615 147

70 65 or 66 or 67 or 68 or 69 149
