# Supplementary material for: Clinical effectiveness of pharmacological interventions for managing chronic migraine in adults: a systematic review and network meta-analysis
Source: J Headache Pain. 2023 Dec 6;24(1):164. doi: 10.1186/s10194-023-01696-w (PMC10702068; doi:10.1186/s10194-023-01696-w)
Supplement: Supplementary file 2 — Additional file 2: Appendix 2. Inclusion and exclusion criteria. Appendix 3. The list of excluded studies. Appendix 4. Baseline characteristics of the 11 RCTs presented in 51 included studies- part 1. Baseline characteristics of the 12 RCTs presented in 55 included studies- part 2. Appendix 5. Network meta-analysis results. Appendix 6. Risk of bias assessment result. Appendix 7. The Grading of Recommendations Assessment, Development and Evaluation (GRADE) approach for rating the quality of estimates of treatment effect size. [file 10194_2023_1696_MOESM2_ESM.docx]

**Additional file 2: Appendix 2. Inclusion and Exclusion Criteria**

| **INCLUSION CRITERIA** | | **EXCLUSION CRITERIA** |
| --- | --- | --- |
| **Population** | - Adults (≥18 years old) with chronic migraine. | - Children and young people aged < 18 years - Participants with menstrual migraine, acute migraine, abdominal migraine, vestibular migraine, or any other conditions-related migraine - Trials that examined participants with other primary headaches including tension-type headaches, cluster headaches, and all sorts of secondary headaches. |
| **Intervention** | - Available or anticipated be available pharmacological medications in the UK: CGRP MAbs, BTA, anti-depressants, ACE inhibitors and angiotensin receptor blockers, beta-blockers, calcium channel blockers, pizotifen, and anticonvulsants (topiramate, valproate/divalproex, gabapentin). | - Studies comparing cognitive-behavioural therapy, psychological interventions, exercise, dietary, and relaxation - Studies which were dose-response trials. - Studies comparing different preparations of the same drug in absence of a placebo - Laboratory studies without clinical outcomes. |
| **Comparator** | - Placebo, or - Usual care, or - Other prophylactic drugs. | - Chinese traditional medicines i.e., herbal medicine/drugs and other herbal remedies which are not prescribed in the UK. - Drugs which are not prescribed by NHS or recommended by NICE or SMC. |
| **Outcomes of interest** | - Headache days - Migraine days - Headache-related QoL - Migraine specified QoL Headache intensity and duration - Health service activity - Days lost from usual activities - Any other reported outcomes | - Non-human outcomes - Outcomes with insufficient information. |
| **Study design** | - RCTs in any setting. - RCTs with more than 100 participants per arm. | - Non-randomised trials, quasi-randomized trials, observational studies (e.g., case reports and case series), subgroup analysis, and other designs. - RCTs with fewer than 100 per arm. |
| Abbreviations: CGRP = calcitonin gene-related peptide; MAbs = monoclonal antibodies; NHS = National Health Service; NICE = National Institute for Health and Care Excellence; QoL = quality of life; RCTs = Randomised controlled trials; SMC = Scottish Medicines Consortium | | |

**Additional file 2: Appendix 3. The list of excluded studies**

| **Publications** | **Reason(s) for exclusion** |
| --- | --- |
| 1.Pradalier A, Rancurel G, Dordain G, Verdure L, Rascol A, Dry J. Acute Migraine Attack Therapy: Comparison of Naproxen Sodium and an Ergotamine Tartrate Compound. Cephalalgia. 1985;5(2):107-113. doi:[10.1046/j.1468-2982.1985.0502107.x](https://doi.org/10.1046/j.1468-2982.1985.0502107.x) | Acute Migraine |
| 2.Abbasi V, Atalu A, Seddighnia P. Comparison of Levetiracetam and sodium Valproate in the prevention of migraine: a randomized clinical trial study. International Journal of Basic & Clinical Pharmacology. 2018 Aug;7(8):1460. | Small sample size of episodic migraine |
| 3.Krakowski AJ, Engisch R. A new agent for chemotherapy of migraine headaches: a controlled study. Psychosomatics: Journal of Consultation and Liaison Psychiatry. 1973 Sep. | Small sample size of episodic migraine |
| 4.Adam EI, Gore SM, Price WH. Double blind trial of clonidine in the treatment of migraine in a general practice. The Journal of the Royal College of General Practitioners. 1978 Oct 1;28(195):587-90. | Small sample size of episodic migraine |
| 5.Soares AD, Louçana PM, Nasi EP, Sousa KM, Sá OM, Silva-Néto RP. A double-blind, randomized, and placebo-controlled clinical trial with omega-3 polyunsaturated fatty acids (OPFA ɷ-3) for the prevention of migraine in chronic migraine patients using amitriptyline. Nutritional neuroscience. 2018 Mar 16;21(3):219-23. | Small sample size of chronic migraine, and not recommended by NICE or SIGN |
| 6.Afshari D, Rafizadeh S, Rezaei M. A comparative study of the effects of low-dose topiramate versus sodium valproate in migraine prophylaxis. International journal of Neuroscience. 2012 Jan 1;122(2):60-8. | Small sample size of episodic migraine |
| 7.Allais G, De Lorenzo C, Quirico PE, Airola G, Tolardo G, Mana O, Benedetto C. Acupuncture in the prophylactic treatment of migraine without aura: a comparison with flunarizine. Headache: The Journal of Head and Face Pain. 2002 Oct;42(9):855-61. | Small sample size of episodic migraine |
| 8. Chabi A, Zhang Y, Jackson S, Cady R, Lines C, Herring WJ, Connor KM, Michelson D. Randomized controlled trial of the orexin receptor antagonist filorexant for migraine prophylaxis. Cephalalgia. 2015 Apr;35(5):379-88. | Not available in the market |
| 9. Andersson PG, Dahl S, Hansen JH, Hansen PE, Hedman C, Nygaard Kristensen T, de Fine Olivarius B. Prophylactic treatment of classical and non‐classical migraine with metoprolol—a comparison with placebo. Cephalalgia. 1983 Dec;3(4):207-12. | Small sample size of episodic migraine |
| 10. Camporeale A, Kudrow D, Sides R, Wang S, Van Dycke A, Selzler KJ, Stauffer VL. A phase 3, long-term, open-label safety study of Galcanezumab in patients with migraine. BMC neurology. 2018 Dec;18(1):1-2. | A safety study, different Galcanezumab doses. |
| 11.Ansell E, Fazzone T, Festenstein R, Johnson ES, Thavapalan M, Wilkinson M, Wozniak I. Nimodipine in migraine prophylaxis. Cephalalgia. 1988 Dec;8(4):269-72. | Small sample size of episodic migraine |
| 12.Bostani A, Rajabi A, Moradian N, Razazian N, Rezaei M. The effects of cinnarizine versus sodium valproate in migraine prophylaxis. International Journal of Neuroscience. 2013 Jul 1;123(7):487-93. | Not clear if chronic, small sample size |
| 13.Ashina M, Doležil D, Bonner JH, Zhou L, Klatt J, Picard H, Mikol DD. A phase 2, randomized, double-blind, placebo-controlled trial of AMG 301, a pituitary adenylate cyclase-activating polypeptide PAC1 receptor monoclonal antibody for migraine prevention. Cephalalgia. 2021 Jan;41(1):33-44. | Not available in the market |
| 14.Assarzadegan F, Tabesh H, Hosseini-Zijoud SM, Beale AD, Shoghli A, Yazdi MG, Mansouri B, Hesami O, Moghadam NB, Kasmaei HD. Comparing zonisamide with sodium valproate in the management of migraine headaches: double-blind randomized clinical trial of efficacy and safety. Iranian Red Crescent Medical Journal. 2016 Sep;18(9). | Small sample size of episodic migraine |
| 15. Barrientos N, Chana P. Botulinum toxin type A in prophylactic treatment of migraine headaches: a preliminary study. The Journal of headache and pain. 2003 Dec;4(3):146-51. | Small sample size of episodic migraine |
| 16.Bavrasad R, Nejad SE, Yarahmadi AR, Sajedi SI, Rahim F. Assessment of the middle dose of topiramate in comparison with sodium valproate for migraine prophylaxis: a randomized-double-blind study. International Journal of Pharmacology. 2010 Sep 1;6(5):670-5. | Small sample size of episodic migraine |
| 17.Beran RG, Spira PJ. Levetiracetam in chronic daily headache: A double-blind, randomised placebo-controlled study: (The Australian KEPPRA Headache Trial [AUS-KHT]). Cephalalgia. 2011 Apr;31(5):530-6. | Small sample size of chronic headache day |
| 18. Bigal ME, Dodick DW, Krymchantowski AV, VanderPluym JH, Tepper SJ, Aycardi E, Loupe PS, Ma Y, Goadsby PJ. TEV-48125 for the preventive treatment of chronic migraine: efficacy at early time points. Neurology. 2016 Jul 5;87(1):41-8. | Small sample size of mixed population of chronic migraine and HFEM. Also, no outcome of interests. |
| 19. Blumenfeld AM, Stevanovic DM, Ortega M, Cohen JM, Seminerio MJ, Yang R, Jiang B, Tepper SJ. No “Wearing‐Off Effect” Seen in Quarterly or Monthly Dosing of Fremanezumab: Subanalysis of a Randomized Long‐Term Study. Headache: The Journal of Head and Face Pain. 2020 Nov;60(10):2431-43. | Wearing-Off Effect study |
| 20. Blumenfeld AM, Schim JD, Chippendale TJ. Botulinum toxin type A and divalproex sodium for prophylactic treatment of episodic or chronic migraine. Headache: The Journal of Head and Face Pain. 2008 Feb;48(2):210-20. | Small size of mixed population |
| 21. Brandes JL, Saper JR, Diamond M, Couch JR, Lewis DW, Schmitt J, Neto W, Schwabe S, Jacobs D, MIGR-002 Study Group, MIGR-002 Study Group. Topiramate for migraine prevention: a randomized controlled trial. Jama. 2004 Feb 25;291(8):965-73. | The mixed population of adults and adolescence |
| 22. Standnes B. The prophylactic effect of timolol versus propranolol and placebo in common migraine: beta-blockers in migraine. Cephalalgia. 1982 Sep;2(3):165-70. | Small sample size of episodic migraine |
| 23. Broessner G, Reuter U, Bonner JH, Dodick DW, Hallström Y, Picard H, Zhang F, Lenz RA, Klatt J, Mikol DD. The spectrum of response to Erenumab in patients with episodic migraine and subgroup analysis of patients achieving≥ 50%,≥ 75%, and 100% response. Headache: The Journal of Head and Face Pain. 2020 Oct;60(9):2026-40. | No report of Adverse Events |
| 24. Bruno MA, Krymchantowski AV. Amitriptyline and intraoral devices for migraine prevention: a randomized comparative trial. Arquivos de Neuro-Psiquiatria. 2018;76:213-8. | Small sample size of episodic migraine |
| 25. R. K. Cady, C. P. Schreiber, J. A. H. Porter, A. M. Blumenfeld, and K. U. Farmer, “A multi-center double-blind pilot comparison of OnabotulinumtoxinA and topiramate for the prophylactic treatment of chronic migraine,” *Headache: The Journal of Head and Face Pain*, vol. 51, no. 1, pp. 21–32, 2011. | Pilot study, small sample size of chronic migraine |
| 26.Cady RK, Voirin J, Farmer K, Browning R, Beach ME, Tarrasch J. Two center, randomized pilot study of migraine prophylaxis comparing paradigms using pre‐emptive frovatriptan or daily topiramate: research and clinical implications. Headache: The Journal of Head and Face Pain. 2012 May;52(5):749-64. | Small sample size of episodic migraine |
| 27.Cao K, Han F, Lin A, Yang W, Zhao J, Zhang H, Ding Y, Xie W, Xu Y, Yu T, Wang X. Zhengtian Capsule versus flunarizine in patients with migraine: a multi-center, double-blind, double-dummy, randomized controlled, non-inferior clinical trial. BMC complementary and alternative medicine. 2016 Dec;16(1):1-0. | Herbal remedy |
| 28. Chankrachang S, Arayawichanont A, Poungvarin N, Nidhinandana S, Boonkongchuen P, Towanabut S, Sithinamsuwan P, Kongsaengdao S. Prophylactic botulinum type A toxin complex (Dysport®) for migraine without aura. Headache: The Journal of Head and Face Pain. 2011 Jan;51(1):52-63. | Small sample size of episodic migraine |
| 29.Charles JA, Jotkowitz S, Byrd LH. Prevention of migraine with olmesartan in patients with hypertension/prehypertension. Headache: The Journal of Head and Face Pain. 2006 Mar;46(3):503-7. | Small sample size of different doses of the same drug, no placebo/control |
| 30. Christensen CE, Younis S, Deen M, Khan S, Ghanizada H, Ashina M. Migraine induction with calcitonin gene-related peptide in patients from Erenumab trials. The Journal of Headache and Pain. 2018 Dec;19(1):1-9. | Question not relevant, not a trial of migraine prevention |
| 31. Couch JR, Amitriptyline Versus Placebo Study Group. Amitriptyline in the prophylactic treatment of migraine and chronic daily headache. Headache: The Journal of Head and Face Pain. 2011 Jan;51(1):33-51. | Small sample size of episodic and chronic migraine |
| . 32. Yang CP, Chang MH, Liu PE, Li TC, Hsieh CL, Hwang KL, Chang HH. Acupuncture versus topiramate in chronic migraine prophylaxis: a randomized clinical trial. Cephalalgia. 2011 Nov;31(15):1510-21. | Small sample size of chronic migraine, and compared with Chinese traditional medicines |
| 33.d'Amato CC, Pizza V, Marmolo T, Giordano E, Alfano V, Nasta A. Fluoxetine for migraine prophylaxis: a double‐blind trial. Headache: The Journal of Head and Face Pain. 1999 Nov;39(10):716-9. | Small sample size of migraine |
| 34.Buse DC, Lipton RB, Hallström Y, Reuter U, Tepper SJ, Zhang F, Sapra S, Picard H, Mikol DD, Lenz RA. Migraine-related disability, impact, and health-related quality of life among patients with episodic migraine receiving preventive treatment with Erenumab. Cephalalgia. 2018 Sep;38(10):1622-31. | No report of Adverse Events |
| 35. Diener HC, Krupp P, Schmitt T, Steitz G, Milde K, Freytag S, Study Group. Cyclandelate in the prophylaxis of migraine: a placebo-controlled study. Cephalalgia. 2001 Feb;21(1):66-70. | No report of Adverse Events |
| 36. Diener HC, F” h M, Laccarino C, Wessely P, Isler H, Stienge H, Fischer M, Wedekind W, Taneri Z. Cyclandelate in the prophylaxis of migraine: a randomized, parallel, double-blind study in comparison with placebo and propranolol. Cephalalgia. 1996 Oct;16(6):441-7. | Small sample size of episodic or chronic migraine |
| 37.Diener HC, Hartung E, Chrubasik JO, Evers S, Schoenen J, Eikermann A, Latta G, Hauke W, Study Group. A comparative study of oral acetylsalicyclic acid and metoprolol for the prophylactic treatment of migraine. A randomized, controlled, double-blind, parallel group phase III study. Cephalalgia. 2001 Mar;21(2):120-8. | No report of Adverse Events |
| 38. Diener HC, Bussone G, Oene JV, Lahaye M, Schwalen S, Goadsby PJ. Topiramate reduces headache days in chronic migraine: a randomized, double-blind, placebo-controlled study. Cephalalgia. 2007 Jul;27(7):814-23. | Small sample size of chronic migraine |
| 39. Dodick DW, Mauskop A, Elkind AH, DeGryse R, Brin MF, Silberstein SD, Botox CDH Study Group. Botulinum toxin type A for the prophylaxis of chronic daily headache: Subgroup analysis of patients not receiving other prophylactic medications: A randomized double‐blind, placebo‐controlled study. Headache: The Journal of Head and Face Pain. 2005 Apr;45(4):315-24. | Chronic daily headache |
| 40. Domingues RB, Silva AL, Domingues SA, Aquino CC, Kuster GW. A double-blind randomized controlled trial of low doses of propranolol, nortriptyline, and the combination of propranolol and nortriptyline for the preventive treatment of migraine. Arquivos de neuro-psiquiatria. 2009;67:973-7. | Small sample size of episodic or chronic migraine |
| 41. Magalhães E, Menezes C, Cardeal M, Melo A. Botulinum toxin type A versus amitriptyline for the treatment of chronic daily migraine. Clinical neurology and neurosurgery. 2010 Jul 1;112(6):463-6. | Small sample size of chronic migraine, and no outcomes of interest |
| 42. Lipton RB, Pozo-Rosich P, Blumenfeld AM, Dodick DW, McAllister P, Li Y, Lu K, Dabruzzo B, Miceli R, Severt L, Finnegan M. Rates of response to Atogepant for migraine prophylaxis among adults: a secondary analysis of a randomized clinical trial. JAMA Network Open. 2022 Jun 1;5(6):e2215499-. | Episodic migraine |
| 43. Faraji F, Zarinfar N, Zanjani AT, Morteza A. The effect of Helicobacter pylori eradication on migraine: a randomized, double blind, controlled trial. Pain physician. 2012;15(6):495. | This is the eradication of HP not use of a prophylactic treatment |
| 44. Ford JH, Ayer DW, Zhang Q, Carter JN, Leroux E, Skljarevski V, Aurora SK, Tockhorn-Heidenreich A, Lipton RB. Two randomized migraine studies of Galcanezumab: effects on patient functioning and disability. Neurology. 2019 Jul 30;93(5):e508-17. | No report of Adverse Events |
| 45. Freitag FG, Diamond S, Diamond M, Urban G. Botulinum toxin type A in the treatment of chronic migraine without medication overuse. Headache: The Journal of Head and Face Pain. 2008 Feb;48(2):201-9. | Small sample size of chronic migraine |
| 46. Lauretti GR, Rosa CP, Kitayama A, Lopes BC. Comparison of botox® or prosigne® and facial nerve blockade as adjuvant in chronic migraine. Journal of Biomedical Science and Engineering. 2014 Jun 26;2014. | Small sample size of chronic migraine. Prosigne is not recommended by NICE or SIGN |
| 47. Ganji R, Majdinasab N, Hesam S, Rostami N, Sayyah M, Sahebnasagh A. Does atorvastatin have augmentative effects with sodium valproate in prevention of migraine with aura attacks? A triple-blind controlled clinical trial. Journal of pharmaceutical health care and sciences. 2021 Dec;7(1):1-0. | Small sample size of episodic migraine |
| 48. Gawel MJ, Kreeft J, Nelson RF, Simard D, Arnott WS. Comparison of the efficacy and safety of flunarizine to propranolol in the prophylaxis of migraine. Canadian journal of neurological sciences. 1992 Aug;19(3):340-5. | Small sample size of episodic migraine |
| 49. Keyvan G, Abolfazl MB. Comparison of treatment effect of sodium valproate, propranolol and tricyclic antidepressants in migraine. Pakistan journal of biological sciences: PJBS. 2009 Aug 1;12(15):1098-101. | Small sample size of episodic or chronic migraine |
| 50. Hussein HS, Alsalihi NJ, Al Gawwam G. Flunarizine Vs Propranolol in the Prevention of Migrainous Headache Attacks. Age (years). 2021;30(9):30-4. | Small sample size of migraine |
| 51. Ghose K, Niven BE, Berry D. A double-blind crossover comparison of the effects of vigabatrin with placebo in the prevention of migraine headache. The Journal of Headache and Pain. 2002 Sep;3(2):79-85. | Small sample size of migraine |
| 52. Goadsby PJ, Silberstein SD, Yeung PP, Cohen JM, Ning X, Yang R, Dodick DW. Long-term safety, tolerability, and efficacy of Fremanezumab in migraine: a randomized study. Neurology. 2020 Nov 3;95(18):e2487-99. | Comparing the dosing regime of same drug |
| 53. Goadsby PJ, Reuter U, Hallström Y, Broessner G, Bonner JH, Zhang F, Wright IK, Chou DE, Klatt J, Picard H, Lenz RA. One-year sustained efficacy of Erenumab in episodic migraine: results of the STRIVE study. Neurology. 2020 Aug 4;95(5):e469-79. | Reporting the active treatment phase with dose blinding not the RCT part |
| 54. Goadsby PJ, Reuter U, Lanteri-Minet M, da Silva Lima GP, Hours-Zesiger P, Fernandes C, Wen S, Tenenbaum N, Kataria A, Ferrari MD, Klatt J. Long-Term efficacy and safety of Erenumab: results from 64 weeks of the liberty study. Neurology. 2021 Jun 1;96(22):e2724-35. | Not an RCT |
| 55. Grahame R. Drug prophylaxis in migraine. British Medical Journal. 1960 Oct 10;2(5207):1203. | Small sample size of episodic migraine |
| 56. Rompel, H. & Bauermeister PW. Aetiology of migraine and prevention with carbamazepine (Tegretol): results of a double-blind, cross-over study. South African Medical Journal. 1970 Jan 1;44(4):75-80. | Small sample size of episodic migraine |
| 57. Havanka‐Kanniainen H, Hokkanen E, Myllylä VV. Efficacy of nimodipine in comparison with pizotifen in the prophylaxis of migraine. Cephalalgia. 1987 Mar;7(1):7-13. | Small sample size of episodic migraine |
| 58. Silberstein SD, Rapoport AM, Loupe PS, Aycardi E, McDonald M, Yang R, Bigal ME. The effect of beginning treatment with Fremanezumab on headache and associated symptoms in the randomized phase 2 study of high frequency episodic migraine: Post‐hoc analyses on the first 3 weeks of treatment. Headache: The Journal of Head and Face Pain. 2019 Mar;59(3):383-93. | Small sample size of episodic migraine |
| 59. Hering R, Kuritzky A. Sodium valproate in the prophylactic treatment of migraine: a double-blind study versus placebo. Cephalalgia. 1992 Apr;12(2):81-4. | Small sample size of episodic migraine |
| 60. Hirata K, Takeshima T, Sakai F, Tatsuoka Y, Suzuki N, Igarashi H, Nakamura T, Ozeki A, Yamazaki H, Skljarevski V. A long-term open-label safety study of Galcanezumab in Japanese patients with migraine. Expert Opinion on Drug Safety. 2021 Jun 3;20(6):721-33. | Not an RCT |
| 61. Hollanda L, Monteiro L, Melo A. Botulinum toxin type A for cephalic cutaneous allodynia in chronic migraine: a randomized, double-blinded, placebo-controlled trial. Neurology International. 2014 Dec 5;6(4):5133. | Assesses cutaneous allodynia rather than headache improvement |
| 62. Hou M, Xie JF, Kong XP, Zhang Y, Shao YF, Wang C, Ren WT, Cui GF, Xin L, Hou YP. Acupoint injection of Onabotulinumtoxin A for migraines. Toxins. 2015 Oct 30;7(11):4442-54. | Small sample size of episodic migraine |
| 63. Mehvari J, Rafieian-Kopaei M. Prophylactic activity of cyproheptadine and Bellergal on migraine headache. Iranian Journal of Medical Sciences. 2005 Jun 1;30(2). | Small sample size of episodic migraine |
| 64. Song JH, Zhang GB, Ding XD, Huang L, Hong Y, Chen HX. Efficacy of type a botulinum toxin injections and infrared polarized light on treating chronic migraine. Eur Rev Med Pharmacol Sci. 2015 Jan 1;19(11):1976-82. | Small sample size of chronic migraine. Infrared polarised light not a UK treatment |
| 65. Gomersall JD, Stuart A. Amitriptyline in migraine prophylaxis: changes in pattern of attacks during a controlled clinical trial. Journal of Neurology, Neurosurgery & Psychiatry. 1973 Aug 1;36(4):684-90. | Small sample size of episodic migraine |
| 66. Jedynak J, Eross E, Gendolla A, Rettiganti M, Stauffer VL. Shift from high-frequency to low-frequency episodic migraine in patients treated with Galcanezumab: results from two global randomized clinical trials. The Journal of Headache and Pain. 2021 Dec;22(1):1-0. | No report of Adverse Events |
| 67. Grotemeyer KH, Schlake HP, Husstedt IW. Etilefrine Pivalate vs. Dihydroergotamin and Flunarizine in Prophylactic Treatment of Migraine in Patients with Low Blood Pressure—A Randomized Double-Blind-Study. Cephalalgia. 1989 Oct;9(10_suppl):433-4. | Small sample size of episodic migraine |
| 68. Welch KM. 11: Naproxen Sodium in the Treatment of Migraine. Cephalalgia. 1986 Apr;6(4_suppl):85-92. | Small sample size of episodic migraine |
| 69. Kuruppu DK, Tobin J, Dong Y, Aurora SK, Yunes-Medina L, Green AL. Efficacy of Galcanezumab in patients with migraine who did not benefit from commonly prescribed preventive treatments. BMC neurology. 2021 Dec;21(1):1-9. | No report of Adverse Events |
| 70. Lai KL, Niddam DM, Fuh JL, Chen SP, Wang YF, Chen WT, Wu JC, Wang SJ. Flunarizine versus topiramate for chronic migraine prophylaxis: a randomized trial. Acta Neurologica Scandinavica. 2017 Apr;135(4):476-83. | Small sample size of chronic migraine |
| 71. Landy S, McGinnis J, Curlin D, Laizure SC. Selective serotonin reuptake inhibitors for migraine prophylaxis. Headache: The Journal of Head and Face Pain. 1999 Jan;39(1):28-32. | Small sample size of episodic migraine |
| 72. Lepcha A, Amalanathan S, Augustine AM, Tyagi AK, Balraj A. Flunarizine in the prophylaxis of migrainous vertigo: a randomized controlled trial. European Archives of Oto-Rhino-Laryngology. 2014 Nov;271(11):2931-6. | Vertigo migraine |
| 73. Lipton RB, Cohen JM, Galic M, Seminerio MJ, Yeung PP, Aycardi E, Bigal ME, Bibeau K, Buse DC. Effects of Fremanezumab in patients with chronic migraine and comorbid depression: subgroup analysis of the randomized HALO CM study. Headache: The Journal of Head and Face Pain. 2021 Apr;61(4):662-72. | Content of this paper is out of scope |
| 74. Loeb LM, Amorim RP, Mazzacoratti MD, Scorza FA, Peres MF. Botulinum toxin A (BT-A) versus low-level laser therapy (LLLT) in chronic migraine treatment: a comparison. Arquivos de neuro-psiquiatria. 2018;76:663-7. | Small sample size of chronic migraine. Low level laser therapy not a UK treatment for migraine |
| 75. Louis P, Spierings EL. Comparison of flunarizine (Sibelium®) and pizotifen (Sandomigran®) in migraine treatment: A double‐blind study. Cephalalgia. 1982 Dec 1;2(4):197-203. | Small sample size of episodic migraine |
| 76. Luo N, Di W, Zhang A, Wang Y, Ding M, Qi W, Zhu Y, Massing MW, Fang Y. A randomized, one-year clinical trial comparing the efficacy of topiramate, flunarizine, and a combination of flunarizine and topiramate in migraine prophylaxis. Pain Medicine. 2012 Jan 1;13(1):80-6. | Small sample size of chronic migraine |
| 77. Siniatchkin M, Andrasik F, Kropp P, Niederberger U, Strenge H, Averkina N, Lindner V, Stephani U, Gerber WD. Central mechanisms of controlled-release metoprolol in migraine: a double-blind, placebo-controlled study. Cephalalgia. 2007 Sep;27(9):1024-32. | Small sample size of episodic migraine |
| 78. Bigal ME, Dodick DW, Rapoport AM, Silberstein SD, Ma Y, Yang R, Loupe PS, Burstein R, Newman LC, Lipton RB. Safety, tolerability, and efficacy of TEV-48125 for preventive treatment of high-frequency episodic migraine: a multicentre, randomised, double-blind, placebo-controlled, phase 2b study. The Lancet Neurology. 2015 Nov 1;14(11):1081-90. | Small sample size of episodic migraine |
| 79. Simmonds MK, Rashiq S, Sobolev IA, Dick BD, Gray DP, Stewart BJ, Jamieson-Lega KI. The effect of single-dose propofol injection on pain and quality of life in chronic daily headache: a randomized, double-blind, controlled trial. Anesthesia & Analgesia. 2009 Dec 1;109(6):1972-80. | Small sample size of chronic daily headache |
| 80. Mathew NT, Frishberg BM, Gawel M, Dimitrova R, Gibson J, Turkel C, Botox CDH Study Group. Botulinum toxin type A (BOTOX®) for the prophylactic treatment of chronic daily headache: A randomized, double‐blind, placebo‐controlled trial. Headache: The Journal of Head and Face Pain. 2005 Apr;45(4):293-307. | Chronic daily headache |
| 81. Mathew NT, Jaffri SF. A double‐blind comparison of Onabotulinumtoxina (BOTOX®) and Topiramate (TOPAMAX®) for the prophylactic treatment of chronic migraine: A pilot study. Headache: The Journal of Head and Face Pain. 2009 Nov;49(10):1466-78. | Small sample size of chronic migraine |
| 82. Mazdeh M, Mahmudian R, Vafaei SY, Taheri M, Ghafouri-Fard S. Effect of propranolol with and without rosuvastatin on migraine attacks: a triple blind randomized clinical trial. Future Neurology. 2020 May;15(2):FNL44. | Small sample size of episodic or chronic migraine |
| 83. Homam M, Farajpour A, Khadem S, Mostafavian Z. The experiential comparison of levetiracetam efficacy in migraine headache with sodium valproate. Caspian Journal of Neurological Sciences. 2016 Jun 10;2(2):42-9. | Small sample size of episodic migraine |
| 84. Millan‐Guerrero RO, Isais‐Millan R, Barreto‐Vizcaino S, Rivera‐Castano L, Garcia‐Solorzano A, López‐Blanca C, Membrila‐Maldonado M, Muñoz‐Solis R. Subcutaneous histamine versus sodium valproate in migraine prophylaxis: A randomized, controlled, double‐blind study. European journal of neurology. 2007 Oct;14(10):1079-84. | Small sample size of episodic migraine |
| 85. Millán‐Guerrero RO, Isais‐Millán S, Barreto‐Vizcaíno S, Rivera‐Castaño L, Rios‐Madariaga C. Subcutaneous histamine versus botulinum toxin type A in migraine prophylaxis: a randomized, double‐blind study. European journal of neurology. 2009 Jan;16(1):88-94. | Small sample size of episodic migraine |
| 86. Mogollón J, Serrano A, Padrón de Freitez A, Uzcátegui E, Baptista T. Olanzapine as an add-on treatment in migraine status: A randomized double-blind, placebo-controlled, pilot study. The European Journal of Psychiatry. 2012 Dec;26(4):260-5. | Acute Migraine |
| 87. Hasan MK, Khan I, Salam T, Sharmin M. The Sociodemographic Characteristics of Migraine Patients in Bangladesh. Int J Med Res Prof. 2019:59-62. | Small sample size of episodic migraine |
| 88. Choudhary MU, Nawaz J, Saddique MU, Zameer A. Comparison between topiramate and sodium valproate efficacy in the treatment of migraine. Pak J Med Health Sci. 2017 Jul 1;11(3):1005-7. | Small sample size of episodic migraine |
| 89. Naderinabi B, Saberi A, Hashemi M, Haghighi M, Biazar G, Gharehdaghi FA, Sedighinejad A, Chavoshi T. Acupuncture and botulinum toxin A injection in the treatment of chronic migraine: a randomized controlled study. Caspian Journal of Internal Medicine. 2017;8(3):196. | Small sample size of chronic migraine. Acupuncture is not medical treatment on NHS. |
| 90. Nappi G, Sandrini G, Savoini G, Cavallini A, De Rysky C, Micieli G. Comparative efficacy of cyclandelate versus flunarizine in the prophylactic treatment of migraine. Drugs. 1987 Mar;33(2):103-9. | Small sample size of episodic migraine |
| 91. Nichols R, Doty E, Sacco S, Ruff D, Pearlman E, Aurora SK. Analysis of initial nonresponders to Galcanezumab in patients with episodic or chronic migraine: results from the EVOLVE‐1, EVOLVE‐2, and REGAIN randomized, double‐blind, placebo‐controlled studies. Headache: The Journal of Head and Face Pain. 2019 Feb;59(2):192-204. | Mixed population |
| 92. Olsson JE, Behring HC, Forssman B, Hedman C, Hedman G, Johansson FA, Kinnman J, Pålhagen SE, Samuelsson M, Strandman E. Metoprolol and propranolol in migraine prophylaxis: a double‐blind multicentre study. Acta neurologica scandinavica. 1984 Sep;70(3):160-8. | Small sample size of episodic migraine |
| 93. Omranifard M, Abdali H, Ardakani MR, Talebianfar M. A comparison of outcome of medical and surgical treatment of migraine headache: In 1 year follow-up. Advanced Biomedical Research. 2016;5. | Small sample size of chronic migraine, compared with surgical treatment. |
| 94. Ondo WG, Vuong KD, Derman HS. Botulinum toxin A for chronic daily headache: a randomized, placebo-controlled, parallel design study. Cephalalgia. 2004 Jan;24(1):60-5. | Mixed of chronic tension type and chronic migraine headaches. |
| 95. Ozyalcin SN, Talu GK, Kiziltan E, Yucel B, Ertas M, Disci R. The efficacy and safety of venlafaxine in the prophylaxis of migraine. Headache: The Journal of Head and Face Pain. 2005 Feb;45(2):144-52. | Small sample size of migraine |
| 96. Kangasniemi P, Hedman C. Metoprolol and propranolol in the prophylactic treatment of classical and common migraine. A double-blind study. Cephalalgia. 1984 Jun;4(2):91-6. | Small sample size of migraine |
| 97. Kangasniemi PJ, Nyrke T, Lang AH, Petersen E. Femoxetine‐a new 5‐HT uptake inhibitor‐and propranolol in the prophylactic treatment of migraine. Acta neurologica scandinavica. 1983 Oct;68(4):262-7. | Drug not available and small sample size of migraine |
| 98. Goadsby PJ, Dodick DW, Ailani J, Trugman JM, Finnegan M, Lu K, Szegedi A. Safety, tolerability, and efficacy of orally administered atogepant for the prevention of episodic migraine in adults: a double-blind, randomised phase 2b/3 trial. The Lancet Neurology. 2020 Sep 1;19(9):727-37. | Small sample size of episodic migraine |
| 99. Pijpers JA, Kies DA, Louter MA, van Zwet EW, Ferrari MD, Terwindt GM. Acute withdrawal and botulinum toxin A in chronic migraine with medication overuse: a double-blind randomized controlled trial. Brain. 2019 May 1;142(5):1203-14. | Small sample size of chronic migraine |
| 100. Pradalier A, Serratrice G, Collard M, Hirsch E, Feve J, Masson M, Masson C, Dry J, Koulikovsky G, Nguyen G, Schbath J. Long-acting propranolol in migraine prophylaxis: results of a double-blind, placebo-controlled study. Cephalalgia. 1989 Dec;9(4):247-53. | Small sample size of episodic or chronic migraine |
| 101. Pradalier A, Lantéri-Minet M, Géraud G, Attain H, Lucas C, Delgado A. The PROMISE study: PROphylaxis of migraine with SEglor®(dihydroergotamine mesilate) in French primary care. CNS drugs. 2004 Dec;18(15):1149-63. | No report of Adverse Events |
| 102. Rahimdel A, Zeinali A, Yazdian-Anari P, Hajizadeh R, Arefnia E. Effectiveness of vitamin B2 versus sodium valproate in migraine prophylaxis: a randomized clinical trial. Electronic Physician. 2015 Oct;7(6):1344 | Small sample size of migraine |
| 103. Rapoport A, Mauskop A, Diener HC, Schwalen S, Pfeil J. Long‐term migraine prevention with topiramate: open‐label extension of pivotal trials. Headache: The Journal of Head and Face Pain. 2006 Jul;46(7):1151-60. | Mixed population of adults and adolescence |
| 104. Rasmussen MJ, Holt Larsen B, Borg L, Soelberg Sørensen P, Hansen PE. Tolfenamic acid versus propranolol in the prophylactic treatment of migraine. Acta neurologica scandinavica. 1994 Jun;89(6):446-50. | Small sample size of episodic or chronic migraine |
| 105. Kaushik R, Kaushik RM, Mahajan SK, Rajesh V. Biofeedback assisted diaphragmatic breathing and systematic relaxation versus propranolol in long term prophylaxis of migraine. Complementary therapies in medicine. 2005 Sep 1;13(3):165-74. | Small sample size of episodic or chronic migraine |
| 106. Lipton RB, Silberstein S, Dodick D, Cady R, Freitag F, Mathew N, Biondi DM, Ascher S, Olson WH, Hulihan J. Topiramate intervention to prevent transformation of episodic migraine: the topiramate INTREPID study. Cephalalgia. 2011 Jan;31(1):18-30. | Mixed population of adults and adolescence |
| 107. Ryan Sr RE. Comparative study of nadolol and propranolol in prophylactic treatment of migraine. American Heart Journal. 1984 Oct 1;108(4):1156-9. | Small sample size of episodic or chronic migraine |
| 108. Ryan RE, Diamond S, Ryan RE. Double blind study of clonidine and placebo for the prophylactic treatment of migraine. Headache: The Journal of Head and Face Pain. 1975 Oct;15(3):202-6. | Small sample size of migraine |
| 109. Ildefonso RL, Martín SA, Francisco HS, Mandeville Peter B, del Rocío RL, Manuel SM, Humberto TP. Topiramate vs. Amitriptyline in prophylactic treatment of migraine: A controlled clinical trial. Rev Mex Neurosci. 2010;11(5):338-42. | Small sample size of migraine |
| 110. Weber RB, Reinmuth OM. The treatment of migraine with propranolol. Neurology. 1972 Apr 1;22(4):366-9. | Small sample size of migraine |
| 111. Ruff DD, Ford JH, Tockhorn‐Heidenreich A, Stauffer VL, Govindan S, Aurora SK, Terwindt GM, Goadsby PJ. Efficacy of Galcanezumab in patients with episodic migraine and a history of preventive treatment failure: results from two global randomized clinical trials. European journal of neurology. 2020 Apr;27(4):609-18. | No report of Adverse Events |
| 112. Evers S, Vollmer-Haase J, Schwaag S, Rahmann A, Husstedt IW, Frese A. Botulinum toxin A in the prophylactic treatment of migraine–a randomized, double-blind, placebo-controlled study. Cephalalgia. 2004 Oct;24(10):838-43. | Small sample size of migraine |
| 113. Ghobadi SH, Jivad N. The prophylactic activity of Propranolol and nimodipineon migraine headache. World J Med Sci. 2013;8(2):144-6. | Small sample size of migraine |
| 114. Sadeghian H, Motiei-Langroudi R. Comparison of Levetiracetam and sodium Valproate in migraine prophylaxis: A randomized placebo-controlled study. Annals of Indian Academy of Neurology. 2015 Jan;18(1):45. | Mixed population of Adolescent and adult |
| 115. Sakai F, Takeshima T, Tatsuoka Y, Hirata K, Lenz R, Wang Y, Cheng S, Hirama T, Mikol DD. A randomized phase 2 study of Erenumab for the prevention of episodic migraine in Japanese adults. Headache: The Journal of Head and Face Pain. 2019 Nov;59(10):1731-42. | Small sample size of episodic migraine |
| 116. Sakai F, Takeshima T, Tatsuoka Y, Hirata K, Cheng S, Numachi Y, Peng C, Xue F, Mikol DD. Long‐term efficacy and safety during open‐label Erenumab treatment in Japanese patients with episodic migraine. Headache: The Journal of Head and Face Pain. 2021 Apr;61(4):653-61. | Small sample size of episodic migraine |
| 117. Santiago MD, Carvalho DD, Gabbai AA, Pinto MM, Moutran AR, Villa TR. Amitriptyline and aerobic exercise or amitriptyline alone in the treatment of chronic migraine: a randomized comparative study. Arquivos de neuro-psiquiatria. 2014;72:851-5. | Small sample size of chronic migraine, compared with exercise. |
| 118. Saper JR, Lake III AE, Cantrell DT, Winner PK, White JR. Chronic daily headache prophylaxis with tizanidine: a double‐blind, placebo‐controlled, multicenter outcome study. Headache: The Journal of Head and Face Pain. 2002 Jun;42(6):470-82. | Small sample size of chronic daily headache |
| 119. Schellenberg R, Lichtenthal A, Wöhling H, Graf C, Brixius K. Nebivolol and Metoprolol for Treating Migraine: An Advance on β‐Blocker Treatment?. Headache: The Journal of Head and Face Pain. 2008 Jan;48(1):118-25. | Small sample size of episodic or chronic migraine |
| 120. Seng EK, Holroyd KA. Behavioral migraine management modifies behavioral and cognitive coping in people with migraine. Headache: The Journal of Head and Face Pain. 2014 Oct;54(9):1470-83. | Not drug trial |
| 121. Bulut S, Berilgen MS, Baran A, Tekatas A, Atmaca M, Mungen B. Venlafaxine versus amitriptyline in the prophylactic treatment of migraine: randomized, double-blind, crossover study. Clinical neurology and neurosurgery. 2004 Dec 1;107(1):44-8. | Small sample size of episodic migraine |
| 122. Sonbolestan SA, Heshmat K, Javanmard SH, Saadatnia M. Efficacy of enalapril in migraine prophylaxis: a randomized, double-blind, placebo-controlled trial. International Journal of Preventive Medicine. 2013 Jan;4(1):72. | Small sample size of episodic migraine |
| 123. Rafie S, Karimian F, Ghomifar A, Karimian A. Effect of Pramipexole on Headache Relief in Patients with Concomitant Migraine and Restless Legs Syndrome; A Randomized, Controlled, Clinical Trial. Jundishapur Journal of Natural Pharmaceutical Products. 2019 Aug 31;14(3). | Small sample size of episodic or chronic migraine |
| 124. Shehata HS, Esmail EH, Abdelalim A, El-Jaafary S, Elmazny A, Sabbah A, Shalaby NM. Repetitive transcranial magnetic stimulation versus botulinum toxin injection in chronic migraine prophylaxis: a pilot randomized trial. Journal of pain research. 2016;9:771. | Small sample size of chronic migraine compared with TMS, which is not on NHS |
| 125. Silberstein SD, Collins SD, Long‐term Safety of Depakote in Headache Prophylaxis Study Group. Safety of divalproex sodium in migraine prophylaxis: An open‐label, long‐term study. Headache: The Journal of Head and Face Pain. 1999 Oct;39(9):633-43. | Not an RCT |
| 126. Silberstein SD, Neto W, Schmitt J, Jacobs D, MIGR-001 Study Group. Topiramate in migraine prevention: results of a large controlled trial. Archives of Neurology. 2004 Apr 1;61(4):490-5. | Mixed population of adults and adolescence |
| 127. Silberstein SD, Dodick DW, Lindblad AS, Holroyd K, Harrington M, Mathew NT, Hirtz D. Randomized, placebo-controlled trial of propranolol added to topiramate in chronic migraine. Neurology. 2012 Mar 27;78(13):976-84. | Small sample size of chronic migraine |
| 128. Silvestrini M, Bartolini M, Coccia M, Baruffaldi R, Taffi R, Provinciali L. Topiramate in the treatment of chronic migraine. Cephalalgia. 2003 Oct;23(8):820-4. | Small sample size of chronic migraine |
| 129. Skljarevski V, Oakes TM, Zhang QI, Ferguson MB, Martinez J, Camporeale A, Johnson KW, Shan Q, Carter J, Schacht A, Goadsby PJ. Effect of different doses of Galcanezumab vs placebo for episodic migraine prevention: a randomized clinical trial. JAMA neurology. 2018 Feb 1;75(2):187-93. | Small sample size of episodic migraine |
| 130. Sørensen PS, Hansen K, Olesen J. A placebo-controlled, double-blind, cross-over trial of flunarizine in common migraine. Cephalalgia. 1986 Mar;6(1):7-14. | Small sample size of migraine |
| 131. Silberstein SD, Loder E, Forde G, Papadopoulos G, Fairclough D, Greenberg S. The impact of migraine on daily activities: effect of Topiramate compared with placebo. Current medical research and opinion. 2006 Jun 1;22(6):1021-9. | No report of Adverse Events |
| 132. Silberstein S, Goode-Sellers S, Twomey C, Saiers J, Ascher J. Randomized, double-blind, placebo-controlled, phase II trial of gabapentin enacarbil for migraine prophylaxis. Cephalalgia. 2013 Jan;33(2):101-11. | Small sample size of episodic migraine |
| 133. Stovner LJ, Linde M, Gravdahl GB, Tronvik E, Aamodt AH, Sand T, Hagen K. A comparative study of candesartan versus propranolol for migraine prophylaxis: A randomised, triple-blind, placebo-controlled, double cross-over study. Cephalalgia. 2014 Jun;34(7):523-32. | Small sample size of episodic or chronic migraine |
| 134. Sujan MU, Rao MR, Kisan R, Abhishekh HA, Nalini A, Raju TR, Sathyaprabha TN. Influence of hydrotherapy on clinical and cardiac autonomic function in migraine patients. Journal of neurosciences in rural practice. 2016 Jan;7(01):109-13. | Not drug trial |
| 135. Zain S, Khan M, Alam R, Zafar I, Ahmed S. Comparison of efficacy and safety of topiramate with gabapentin in migraine prophylaxis: randomized open label control trial. J Pak Med Assoc. 2013 Jan 1;63(1):3-7. | Small sample size of episodic migraine |
| 136. Tatsuoka Y, Takeshima T, Ozeki A, Matsumura T. Treatment Satisfaction of Galcanezumab in Japanese Patients with Episodic Migraine: A Phase 2 Randomized Controlled Study. Neurology and therapy. 2021 Jun;10(1):265-78. | No report of Adverse Events |
| 137. Oakes TM, Skljarevski V, Zhang Q, Kielbasa W, Hodsdon ME, Detke HC, Camporeale A, Saper JR. Safety of Galcanezumab in patients with episodic migraine: a randomized placebo-controlled dose-ranging phase 2b study. Cephalalgia. 2018 May;38(6):1015-25. | Small sample size of episodic migraine |
| 138. Titus F, Dávalos A, Alom J, Codina A. 5-hydroxytryptophan versus methysergide in the prophylaxis of migraine. European neurology. 1986;25(5):327-9. | Small sample size of migraine |
| 139. Steiner TJ, Ahmed F, Findley LJ, MacGregor EA, Wilkinson M. S‐fluoxetine in the prophylaxis of migraine: a phase II double‐blind randomized placebo‐controlled study. Cephalalgia. 1998 Jun;18(5):283-6. | Small sample size of migraine |
| 140. Vahedi K, Taupin P, Djomby RF, El-Amrani M, Lutz G, Filipetti V, Landais P, Massiou H, Bousser MG. Efficacy and tolerability of acetazolamide in migraine prophylaxis: a randomised placebo-controlled trial. Journal of neurology. 2002 Feb;249(2):206-11. | Small sample size of episodic migraine |
| 141. Todorov V, Bogdanova D, Tonchev P, Milanov I. Repetitive transcranial magnetic stimulation over two target areas, sham stimulation and topiramate in the treatment of chronic migraine. Comptes rendus de l’Académie bulgare des Sciences. 2020 Jan 1;73(9). | Small sample size of chronic migraine compared with TMS which is not on NHS. |
| 142. Villani V, Prosperini L, Palombini F, Orzi F, Sette G. Single-blind, randomized, pilot study combining shiatsu and amitriptyline in refractory primary headaches. Neurological Sciences. 2017 Jun;38(6):999-1007. | Small sample size of episodic migraine |
| 143. Wammes‐van der EA, Smidt MH, Tijssen CC, Van ‘t Hoff AR, Lenderink, PharmD AW, Egberts, PharmD AC. Effect of low‐intensity acenocoumarol on frequency and severity of migraine attacks. Headache: The Journal of Head and Face Pain. 2005 Feb;45(2):137-43. | Small sample size of episodic or chronic migraine |
| 144. Xu JH, Mi HY. A randomized controlled trial of acupressure as an adjunctive therapy to sodium valproate on the prevention of chronic migraine with aura. Medicine. 2017 Jul;96(27). | Not a drug trial |
| 145. Yurekli VA, Akhan G, Kutluhan S, Uzar E, Koyuncuoglu HR, Gultekin F. The effect of sodium valproate on chronic daily headache and its subgroups. The journal of headache and pain. 2008 Feb;9(1):37-41. | Small sample size on chronic daily headache |
| 146. Zidan A, Hussaini S, Gibson S, Brooks G, Mejico L. Onabotulinumtoxin Type A reconstitution with preserved versus preservative‐free saline in chronic migraine (B‐RECON). A randomised, double‐blind trial. International Journal of Clinical Practice. 2020 Sep;74(9):e13522. | Trial of Botox preservative on injection site pain |
| 147. Silberstein SD, Hulihan J, Karim MR, Wu SC, Jordan D, Karvois D, Kamin M. Efficacy and tolerability of topiramate 200 mg/d in the prevention of migraine with/without aura in adults: a randomized, placebo-controlled, double-blind, 12-week pilot study. Clinical therapeutics. 2006 Jul 1;28(7):1002-11. | Small sample size of episodic migraine |
| 148. Askari G, Nasiri M, Mozaffari-Khosravi H, Rezaie M, Bagheri-Bidakhavidi M, Sadeghi O. The effects of folic acid and pyridoxine supplementation on characteristics of migraine attacks in migraine patients with aura: A double-blind, randomized placebo-controlled, clinical trial. Nutrition. 2017 Jun 1;38:74-9. | Acute migraine |
| 149. Hajihashemi P, Askari G, Khorvash F, Reza Maracy M, Nourian M. The effects of concurrent Coenzyme Q10, L-carnitine supplementation in migraine prophylaxis: A randomized, placebo-controlled, double-blind trial. Cephalalgia. 2019 Apr;39(5):648-54. | Small sample size of episodic migraine |
| 150. Bredfeldt RC, Sutherland JE, Kruse JE. Efficacy of transdermal clonidine for headache prophylaxis and reduction of narcotic use in migraine patients. J Fam Pract. 1989;29(2):153-6. | Small sample size of episodic migraine Small episodic migraine |
| 151. Centonze V, Macinagrossa G, Attolini E, Magrone D, Trizio T, Tesauro P, Campanozzi F, Altomare E, Albano O. Terapia preventiva dell'emicrania: flunarizina versus verapamil [Preventive therapy of migraine: flunarizine versus verapamil]. Clin Ter. 1985 Dec 15;115(5):333-9. Italian. PMID: 3830537. | Small sample size of episodic migraine |
| 152. Maizels M, Blumenfeld A, Burchette R. A combination of riboflavin, magnesium, and feverfew for migraine prophylaxis: a randomized trial. Headache: The Journal of Head and Face Pain. 2004 Oct;44(9):885-90. | Small sample size of episodic migraine |
| 153. Chitsaz A, Ghorbani A, Hoseinzadeh H, Nazari F, Norouzi R, Tajic S. Comparison of botulinum toxin type-A and divalproex sodium for prevention of chronic and episodic migraine. Neurology Asia. 2012 Jun 1;17(2). | Small sample size of episodic or chronic migraine |
| 154. Matin H, Taghian F, Chitsaz A. Artificial intelligence analysis to explore synchronize exercise, cobalamin, and magnesium as new actors to therapeutic of migraine symptoms: a randomized, placebo-controlled trial. Neurological Sciences. 2022 Feb 3:1-2. | Small sample size of episodic migraine |
| 155. Croop R, Lipton RB, Kudrow D, Stock DA, Kamen L, Conway CM, Stock EG, Coric V, Goadsby PJ. Oral rimegepant for preventive treatment of migraine: a phase 2/3, randomised, double-blind, placebo-controlled trial. The Lancet. 2021 Jan 2;397(10268):51-60. | Small sample size of episodic or chronic migraine |
| 156. Diamond S, Kudrow L, Stevens J, Shapiro DB. Long‐term study of propranolol in the treatment of migraine. Headache: The Journal of Head and Face Pain. 1982 Nov;22(6):268-71. | No outcome of interests |
| 157. Mottaghi T, Askari G, Khorvash F, Maracy MR. Effect of Vitamin D supplementation on symptoms and C-reactive protein in migraine patients. Journal of research in medical sciences: the official journal of Isfahan University of Medical Sciences. 2015 May;20(5):477. | Mixed population of adults and adolescence |
| 158. Nattagh-Eshtivani E, Dahri M, Hashemilar M, Tarighat-Esfanjani A. The effect of coenzyme Q10 supplementation on serum levels of lactate, pyruvate, matrix metalloproteinase 9 and nitric oxide in women with migraine. A double blind, placebo, controlled randomized clinical trial. European Journal of Integrative Medicine. 2018 Aug 1;21:70-6. | Small sample size of episodic migraine |
| 159. Shoeibi A, Olfati N, Soltani Sabi M, Salehi M, Mali S, Akbari Oryani M. Effectiveness of coenzyme Q10 in prophylactic treatment of migraine headache: an open-label, add-on, controlled trial. Acta Neurologica Belgica. 2017 Mar;117(1):103-9. | Small sample size of episodic migraine |
| 160. Tarighat Esfanjani A, Mahdavi R, Ebrahimi Mameghani M, Talebi M, Nikniaz Z, Safaiyan A. The effects of magnesium, l-carnitine, and concurrent magnesium–l-carnitine supplementation in migraine prophylaxis. Biological trace element research. 2012 Dec;150(1):42-8. | Small sample size of episodic migraine |
| 161. Hsieh LL, Liou HH, Lee LH, Chen TH, Yen AM. Effect of acupressure and trigger points in treating headache: a randomized controlled trial. The American Journal of Chinese Medicine. 2010;38(01):1-4. | Small sample size of episodic migraine |
| 162. Langohr HD, Gerber WD, Koletzki E, Mayer K, Schroth G. Clomipramine and Metoprolol in Migraine Prophylaxis—A Double‐blind Crossover Study. Headache: The Journal of Head and Face Pain. 1985 Mar;25(2):107-12. | Small sample size of episodic migraine |
| 163. Sândor PS, Di Clemente L, Coppola G, Saenger U, Fumal A, Magis D, Seidel L, Agosti RM, Schoenen J. Efficacy of coenzyme Q10 in migraine prophylaxis: a randomized controlled trial. Neurology. 2005 Feb 22;64(4):713-5. | Small sample size of episodic migraine |
| 164. Mathew NT. Prophylaxis of migraine and mixed headache. A randomized controlled study. Headache: The Journal of Head and Face Pain. 1981 May;21(3):105-9. | Small sample size of migraine |
| 165. Schoenen J, Jacquy J, Lenaerts M. Effectiveness of high‐dose riboflavin in migraine prophylaxis A randomized controlled trial. Neurology. 1998 Feb 1;50(2):466-70. | Small sample size of episodic migraine |
| 166. Cao K, Yu L, Gao Y, Fan Y, Zhao J, Zhang X, Xie W, Yang W, Dong M, Li T, Qiao X. Efficacy of zhengtian pill for migraine prophylaxis: a randomized, multicenter, double-blind, placebo-controlled, parallel-group study. European Journal of Integrative Medicine. 2014 Jun 1;6(3):259-67. | Episodic migraine |
| 167. Nelson CF, Bronfort G, Evans R, Boline P, Goldsmith C, Anderson AV. The efficacy of spinal manipulation, amitriptyline and the combination of both therapies for the prophylaxis of migraine headache. Journal of manipulative and physiological therapeutics. 1998 Oct 1;21(8):511-9. | Small sample size of episodic migraine |
| 168. Diener HC, Kronfeld K, Boewing G, Lungenhausen M, Maier C, Molsberger A, Tegenthoff M, Trampisch HJ, Zenz M, Meinert R, GERAC Migraine Study Group. Efficacy of acupuncture for the prophylaxis of migraine: a multicentre randomised controlled clinical trial. The Lancet Neurology. 2006 Apr 1;5(4):310-6. | Episodic, Control treatment not standardized |
| 169. Shukla R, Garg RK, Nag D, Ahuja RC. Nifedipine in migraine and tension headache: a randomised double blind crossover study. The Journal of the Association of Physicians of India. 1995 Nov 1;43(11):770-2. | Small sample size of episodic migraine |
| 170. Silberstein SD, Stark SR, Lucas SM, Christie SN, Degryse RE, Turkel CC, BoNTA-039 Study Group. Botulinum toxin type A for the prophylactic treatment of chronic daily headache: a randomized, double-blind, placebo-controlled trial. In Mayo Clinic Proceedings 2005 Sep 1 (Vol. 80, No. 9, pp. 1126-1137). Elsevier. | Paper has included mixed patients (77% chronic migraine, 18% chronic tension type headache, 3% new daily persistent headache and 1% other headaches). Hence, not all patients were chronic migraineurs. |
| 171. Ford JH, Stauffer VL, McAllister P, Akkala S, Sexson M, Ayer DW, Wang S. Functional impairment and disability among patients with migraine: evaluation of Galcanezumab in a long-term, open-label study. Quality of Life Research. 2021 Feb;30(2):455-64. | Small sample size of episodic migraine |
| 172. Anthony M. Interesting uses of beta blockers. 1. Beta blockers in migraine prevention. Australian family physician. 1981 Apr;10(4):258-62. | Not an RCT |
| 173. Arthur GP, Hornabrook RW. The treatment of migraine with BC 105 (pizotifen): a double blind trial. The New Zealand Medical Journal. 1971 Jan 1;73(464):5-9. | Small sample size of episodic migraine |
| 174. Ashkenazi A, Silberstein S. Botulinum toxin type A for the treatment of headache: why we say yes. Archives of neurology. 2008 Jan 1;65(1):146-9. | Not an RCT |
| 175. Bademosi O, Osuntokun BO. Pizotifen in the management of migraine. The Practitioner. 1978 Feb 1;220(1316):325-7. | Small sample size of episodic migraine |
| 176. Behan PO, Reid M. Propranolol in the treatment of migraine. The Practitioner. 1980 Feb;224(1340):201-3. | Small sample size of episodic migraine |
| 177. Chen SP, Fuh JL, Wang SJ. OnabotulinumtoxinA: preventive treatment for chronic migraine. Current pain and headache reports. 2011 Feb;15(1):4-7. | Not an RCT |
| 178. Diamond S, Freitag FG. A double-blind trial of flunarizine in migraine prophylaxis. Headache Quarterly-Current Treatment and Research. 1993 Jan 1;4(2):169-72. | Small sample size of episodic migraine |
| 179. Feuerstein T, Quebe-Fehling E. A double-blind, placebo-controlled, parallel-group, multicenter study of the safety and efficacy of gabapentin (CI-945) as a prophylactic interval therapy in patients with common migraine (Protocols 879-201,-205,-206,-207,-209). Research Report No. RR 4301-00066. Freiburg: Goedecke AG Research and Development. Available at: http://dida. library. ucsf. edu/pdf/brr13j10 1990. Accessed May 26; 2015. | Small sample size of episodic migraine |
| 180. Hübbe P. Controlled clinical trials of drugs for use in the prophylaxis of migraine. Danish Medical Bulletin. 1975 Mar 1;22(3):92-6. | Not an RCT |
| 181. Lance JW, Curran DA, Anthony M. Investigations into the mechanism and treatment of chronic headache. Medical journal of Australia. 1965 Nov;2(22):909-14. | Not an RCT |
| 182. Magnus-Miller L, Bernstein P, Caswell K. Double-blind, randomized, placebo-controlled, multicenter trial to determine the efficacy and safety of Neurontin®(gabapentin) in migraine prophylaxis administered in doses divided three times a day (TID)(protocol 945-220). Research Report No. RR 995‐00074. Morris Plains, NJ: Parke‐Davis Pharmaceutical Research Division of Warner‐Lambert Company Medical and Scientific Affairs Department; August 24, 1999. Available at: http://dida. library. ucsf. edu/pdf/arr13j10. 1999 Aug 24. | Small sample size of episodic migraine |
| 183. Magnus-Miller L, Bernstein P, Caswell K. Double-blind, randomized, placebo-controlled, multicenter trial to determine the efficacy and safety of Neurontin®(gabapentin) in migraine prophylaxis administered in doses divided three times a day (TID)(protocol 945-220). Research Report No. RR 995‐00074. Morris Plains, NJ: Parke‐Davis Pharmaceutical Research Division of Warner‐Lambert Company Medical and Scientific Affairs Department; August 24, 1999. Available at: http://dida. library. ucsf. edu/pdf/arr13j10. 1999 Aug 24. | Small sample size of episodic migraine |
| 184. Linde K, Rossnagel K. WITHDRAWN: Propranolol for migraine prophylaxis. Cochrane Database Syst Rev. 2017;2(2):CD003225. Published 2017 Feb 17. doi:10.1002/14651858.CD003225.pub3 | Not an RCT |
| 185. Nair KG. A pilot study of the value of propranolol in migraine. Journal of postgraduate medicine. 1975 Jul;21(3):111-3. | Small sample size of episodic migraine |
| 186. Pita E, Higueras A, Bolanos J, Perez N, Mundo A. Propranolol and migraine. A clinical trial. Archivos de Farmacologia y Toxicologia. 1977 Dec 1;3(3):273-8. | Small sample size of episodic migraine |
| 187. Verstraete M, Verhaeghe R. 20 Drugs acting on the cerebral and peripheral. Side Effects of Drugs Annual. 1977;10:172. | Small sample size of episodic migraine |
| 188. Rothrock JF. Topiramate for chronic migraine. | Not an RCT |
| 189. Ryan Sr RE, Ryan Jr RE, Sudilovsky A. Nadolol and placebo comparison study in the prophylactic treatment of migraine. Panminerva medica. 1982;24(2):89-94. | Small sample size of episodic migraine |
| 190. Saper JR, Good DC, Michalek J, Kaplan RJ, Xiao Y, Nadler S. Efficacy and tolerability of tizanidine as adjunctive therapy in the prophylaxis of chronic daily headache. Round Table Series-Royal Society of Medicine. 2002 Jan 1(75):13-22. | Small sample size of episodic migraine |
| 191. Yuan C, Wang JX, Yu JP, Yan F, Su SH. A double-blind trial of 5 calcium channel blockers in prophylaxis of migraine. CHINESE JOURNAL OF CLINICAL PHARMACOLOGY. 1998;14:14-7. | Small sample size of episodic migraine |
| 192. Mattimoe D, Newton W. High-dose riboflavin for migraine prophylaxis. The Journal of Family Practice. 1998 Jul 1;47(1):11-. | Small sample size of episodic migraine |
| 193. Gonçalves DA, Camparis CC, Speciali JG, et al.Treatment of comorbid migraine and temporo-mandibular disorders: A factorial, double-blind, randomized, placebo-controlled study. J Orofac Pain. 2013;27:325-335 | Small sample size of episodic migraine |
| 194. Schoenen J, Jacquy J, Lenaerts M. High-dose riboflavin is effective in migraine prophylaxis: Results from a double blind, randomized, placebo controlled trial. In Neurology 1997 Mar 1 (Vol. 48, No. 3, pp. 8005-8005). 227 EAST WASHINGTON SQ, PHILADELPHIA, PA 19106: LIPPINCOTT-RAVEN PUBL. | Small sample size of episodic migraine |
| 195. Hübbe P. The prophylactic treatment of migraine with an antiserotonin pizotifen (BC 105). Acta Neurologica Scandinavica. 1973 Mar;49(1):108-14. | Small sample size of episodic migraine |
| 196. Schoenen J, Jacquy J, Lenaerts ME, Loder E. High-dose riboflavin reduced the frequency of migraine headaches. Evidence-Based Medicine. 1998 Sep;3(5):151. | Small sample size of episodic migraine |
| 197. Rezaeiashtiani A, Jadidi A, Khanmohammadi-Hezaveh A, Aghaeipour SM, Pourandish Y, Malekhosseini S, Ghassami K, Mohammadbeigi A. Is the treatment of constipation can relieve the migraine symptoms? A randomized clinical trial study. Journal of Pediatric Neurosciences. 2019 Oct;14(4):186. | Small sample size of episodic migraine |
| 198. Shimell CJ, Fritz VU, Levien SL. A comparative trial of flunarizine and propranolol in the prevention of migraine. South African Medical Journal, 1990 Jan 1;77(2):75-7. | Small sample size of episodic migraine |
| 199. Wang LP, Zhang XZ, Guo J, Liu HL, Zhang Y, Liu CZ, Yi JH, Wang LP, Zhao JP, Li SS. Efficacy of acupuncture for migraine prophylaxis: a single-blinded, double-dummy, randomized controlled trial. PAIN®. 2011 Aug 1;152(8):1864-71. | Small sample size of episodic migraine |
| 200. Yang CP, Chang MH, Li TC, Hsieh CL, Hwang KL, Chang HH. Predicting prognostic factors in a randomized controlled trial of acupuncture versus topiramate treatment in patients with chronic migraine. The Clinical Journal of Pain. 2013 Nov 1;29(11):982-7. | Small sample size of chronic migraine compared with acupuncture. |
| 201. Manzoni, GC, et all., Multicentric, double-blind, randomized study on the efficacy and tolerance of dothiepin and amitriptyline for the prophylaxis of chronic migraine. Giornale di neuropsicofarmacologia. 1990; 12(5):179-184 | Small sample size of chronic migraine |
| 202. Lipton RB, Croop R, Stock EG, Stock DA, Morris BA, Frost M, Dubowchik GM, Conway CM, Coric V, Goadsby PJ. Rimegepant, an oral calcitonin gene–related peptide receptor antagonist, for migraine. New England Journal of Medicine. 2019 Jul 11;381(2):142-9. | Acute migraine |
| 203. Diener HC, Tfelt-Hansen P, Dahlöf C, et al. Topiramate in migraine prophylaxis. J Neurol 251, 943– 950 (2004). <https://doi.org/10.1007/s00415-004-0464->6 | Mixed population of adult and adolescents |
| 204. Ailani J, Andrews JS, Tockhorn-Heidenreich A, Wenzel R, Rettiganti M. Effect of Galcanezumab on Total Pain Burden in Patients Who Had Previously Not Benefited from Migraine Preventive Medication (CONQUER Trial): A Post Hoc Analysis. Advances in Therapy. 2022 Oct;39(10):4544-55. | No safety data for adverse events review and a small sample size of chronic migraine |
| 205. Igarashi H, Shibata M, Ozeki A, Matsumura T. Galcanezumab Effects on Migraine Severity and Symptoms in Japanese Patients with Episodic Migraine: Secondary Analysis of a Phase 2 Randomized Trial. Neurology and Therapy. 2022 Oct 20:1-5. | No safety data for adverse events review |
| 206. Chowdhury D, Chaudhuri JR, Ghosh P, Kulkarni R, Singh S, Thakur S, Thorat AV. Efficacy and tolerability of Erenumab for prevention of episodic migraine in India. Annals of Indian Academy of Neurology. 2022 May;25(3):433. | Small sample of episodic migraine |
| 207. Igarashi H, Shibata M, Ozeki A, Day KA, Matsumura T. Early Onset and Maintenance Effect of Galcanezumab in Japanese Patients with Episodic Migraine. Journal of Pain Research. 2021;14:3555. | No safety data for adverse events review |
| 208. Sakai F, Takeshima T, Tatsuoka Y, Hirata K, Cheng S, Numachi Y, Peng C, Xue F, Mikol DD. Long‐term efficacy and safety during open‐label Erenumab treatment in Japanese patients with episodic migraine. Headache: The Journal of Head and Face Pain. 2021 Apr;61(4):653-61. | Small sample of episodic migraine |
| 209. Okonkwo R, Tockhorn-Heidenreich A, Stroud C, Paget MA, Matharu MS, Tassorelli C. Efficacy of Galcanezumab in patients with migraine and history of failure to 3–4 preventive medication categories: subgroup analysis from CONQUER study. The journal of headache and pain. 2021 Dec;22(1):1-1. | No safety data for adverse events review |
| 210. Okonkwo R, Tockhorn-Heidenreich A, Stroud C, Paget MA, Matharu MS, Tassorelli C. Efficacy of Galcanezumab in patients with migraine and history of failure to 3–4 preventive medication categories: subgroup analysis from CONQUER study. The journal of headache and pain. 2021 Dec;22(1):1-1. | No safety data for adverse events review |
| 211. Ailani J, Lipton RB, Goadsby PJ, Guo H, Miceli R, Severt L, Finnegan M, Trugman JM. Atogepant for the preventive treatment of migraine. New England Journal of Medicine. 2021 Aug 19;385(8):695-706. | Episodic migraine |
| 212. Ashina M, Saper J, Cady R, Schaeffler BA, Biondi DM, Hirman J, Pederson S, Allan B, Smith J. Eptinezumab in episodic migraine: a randomized, double-blind, placebo-controlled study (PROMISE-1). Cephalalgia. 2020 Mar;40(3):241-54. | Episodic migraine |
| 213. Ashina, M., Cohen, J.M., Galic, M., Campos, V.R., Barash, S., Ning, X., Kessler, Y., Janka, L. and Diener, H.C., 2021. Efficacy and safety of Fremanezumab in patients with episodic and chronic migraine with documented inadequate response to 2 to 4 classes of migraine preventive medications over 6 months of treatment in the phase 3b FOCUS study. The Journal of Headache and Pain, 22(1), pp.1-13. | Episodic migraine |
| 214. Ashina M, Goadsby PJ, Reuter U, Silberstein S, Dodick DW, Xue F, Zhang F, Paiva da Silva Lima G, Cheng S, Mikol DD. Long‐term efficacy and safety of Erenumab in migraine prevention: results from a 5‐year, open‐label treatment phase of a randomized clinical trial. European journal of neurology. 2021 May;28(5):1716-25. | Episodic migraine |
| 215. Aurora SK, Gawel M, Brandes JL, Pokta S, VanDenburgh AM, BOTOX North American Episodic Migraine Study Group. Botulinum toxin type a prophylactic treatment of episodic migraine: A randomized, double‐blind, placebo‐controlled exploratory study. Headache: The Journal of Head and Face Pain. 2007 Apr;47(4):486-99. | Episodic migraine |
| 216. Brandes JL, Saper JR, Diamond M, Couch JR, Lewis DW, Schmitt J, Neto W, Schwabe S, Jacobs D, MIGR-002 Study Group, MIGR-002 Study Group. Topiramate for migraine prevention: a randomized controlled trial. Jama. 2004 Feb 25;291(8):965-73. | Episodic migraine |
| 217. Brandes JL, Diener HC, Dolezil D, Freeman MC, McAllister PJ, Winner P, Klatt J, Cheng S, Zhang F, Wen S, Ritter S. The spectrum of response to Erenumab in patients with chronic migraine and subgroup analysis of patients achieving≥ 50%,≥ 75%, and 100% response. Cephalalgia. 2020 Jan;40(1):28-38. | Episodic migraine |
| 218. Couch JR, Amitriptyline Versus Placebo Study Group. Amitriptyline in the prophylactic treatment of migraine and chronic daily headache. Headache: The Journal of Head and Face Pain. 2011 Jan;51(1):33-51. | Episodic migraine |
| 219. Dodick DW, Goadsby PJ, Spierings EL, Scherer JC, Sweeney SP, Grayzel DS. Safety and efficacy of LY2951742, a monoclonal antibody to calcitonin gene-related peptide, for the prevention of migraine: a phase 2, randomised, double-blind, placebo-controlled study. The Lancet Neurology. 2014 Sep 1;13(9):885-92. | Episodic migraine |
| 220. Dodick DW, Ashina M, Brandes JL, Kudrow D, Lanteri-Minet M, Osipova V, Palmer K, Picard H, Mikol DD, Lenz RA. ARISE: a phase 3 randomized trial of Erenumab for episodic migraine. Cephalalgia. 2018 May;38(6):1026-37. | Episodic migraine |
| 221. Dodick DW, Freitag F, Banks J, Saper J, Xiang J, Rupnow M, Biondi D, Greenberg SJ, Hulihan J, CAPSS-277 Investigator Group. Topiramate versus amitriptyline in migraine prevention: a 26-week, multicenter, randomized, double-blind, double-dummy, parallel-group noninferiority trial in adult migraineurs. Clinical therapeutics. 2009 Mar 1;31(3):542-59. | Episodic migraine |
| 222. Diener HC, Matias-Guiu J, Hartung E, Pfaffenrath V, Ludin HP, Nappi G, De Beukelaar F, Study Group. Efficacy and tolerability in migraine prophylaxis of flunarizine in reduced doses: a comparison with propranolol 160 mg daily. Cephalalgia. 2002 Apr;22(3):209-21. | Episodic migraine |
| 223. Diener HC, Tfelt-Hansen P, Dahlöf C, Láinez MJ, Sandrini G, Wang SJ, Neto W, Vijapurkar U, Doyle A, Jacobs D, MIGR-003 Study Group. Topiramate in migraine prophylaxis: Results from a placebo–controlled trial with propranolol as an active control. Journal of neurology. 2004 Aug;251:943-50. | Episodic migraine |
| 224. Dodick DW, Silberstein SD, Bigal ME, Yeung PP, Goadsby PJ, Blankenbiller T, Grozinski-Wolff M, Yang R, Ma Y, Aycardi E. Effect of Fremanezumab compared with placebo for prevention of episodic migraine: a randomized clinical trial. Jama. 2018 May 15;319(19):1999-2008. | Episodic migraine |
| 225. Goadsby PJ, Reuter U, Hallström Y, Broessner G, Bonner JH, Zhang F, Sapra S, Picard H, Mikol DD, Lenz RA. A controlled trial of Erenumab for episodic migraine. New England Journal of Medicine. 2017 Nov 30;377(22):2123-32. | Episodic migraine |
| 226. Sun H, Dodick DW, Silberstein S, Goadsby PJ, Reuter U, Ashina M, Saper J, Cady R, Chon Y, Dietrich J, Lenz R. Safety and efficacy of AMG 334 for prevention of episodic migraine: a randomised, double-blind, placebo-controlled, phase 2 trial. The Lancet Neurology. 2016 Apr 1;15(4):382-90. | Episodic migraine |
| 227. Jedynak J, Eross E, Gendolla A, Rettiganti M, Stauffer VL. Shift from high-frequency to low-frequency episodic migraine in patients treated with Galcanezumab: results from two global randomized clinical trials. The Journal of Headache and Pain. 2021 Dec;22(1):1-0. | Episodic migraine |
| 228. Kalita J, Bhoi SK, Misra UK. Amitriptyline vs divalproate in migraine prophylaxis: a randomized controlled trial. Acta Neurologica Scandinavica. 2013 Jul;128(1):65-72. | Episodic migraine |
| 229. Hirata K, Sakai F, Takeshima T, Imai N, Matsumori Y, Yoshida R, Numachi Y, Peng C, Mikol DD, Cheng S. Efficacy and safety of Erenumab in Japanese migraine patients with prior preventive treatment failure or concomitant preventive treatment: subgroup analyses of a phase 3, randomized trial. The Journal of Headache and Pain. 2021 Dec;22(1):1-9. | Episodic migraine |
| 230. Yang CP, Chang MH, Li TC, Hsieh CL, Hwang KL, Chang HH. Predicting prognostic factors in a randomized controlled trial of acupuncture versus topiramate treatment in patients with chronic migraine. The Clinical Journal of Pain. 2013 Nov 1;29(11):982-7. | Episodic migraine |
| 231. Lipton RB, Croop R, Stock EG, Stock DA, Morris BA, Frost M, Dubowchik GM, Conway CM, Coric V, Goadsby PJ. Rimegepant, an oral calcitonin gene–related peptide receptor antagonist, for migraine. New England Journal of Medicine. 2019 Jul 11;381(2):142-9. | Episodic migraine |
| 232. Lucking CH, Oestreich W, Schmidt R, Soyka D. Flunarizine vs. propranolol in the prophylaxis of migraine: two double-blind comparative studies in more than 400 patients. Cephalalgia. 1988 Sep;8(8_suppl):21-6. | Episodic migraine |
| 233. Relja M, Poole AC, Schoenen J, Pascual J, Lei X, Thompson C. A multicentre, double-blind, randomized, placebo-controlled, parallel group study of multiple treatments of botulinum toxin type A (BoNTA) for the prophylaxis of episodic migraine headaches. Cephalalgia. 2007 Jun;27(6):492-503. | Episodic migraine |
| 234. Janssen Pharmaceutical KK. A Dose-Finding Study of Topiramate (JNS019) in Participants with Migraine. NIH US National Library of Medicine Clinical Trials. gov.[Revised on July 2013][Accessed 23rd November 2020] https://clinicaltrials. gov/ct2/show/NCT01081795. | Episodic migraine |
| 235. Goadsby PJ, Paemeleire K, Broessner G, Brandes J, Klatt J, Zhang F, Picard H, Lenz R, Mikol DD. Efficacy and safety of Erenumab (AMG334) in episodic migraine patients with prior preventive treatment failure: a subgroup analysis of a randomized, double-blind, placebo-controlled study. Cephalalgia. 2019 Jun;39(7):817-26. | Episodic migraine |
| 236. Goadsby PJ, Dodick DW, Ailani J, Trugman JM, Finnegan M, Lu K, Szegedi A. Safety, tolerability, and efficacy of orally administered atogepant for the prevention of episodic migraine in adults: a double-blind, randomised phase 2b/3 trial. The Lancet Neurology. 2020 Sep 1;19(9):727-37. | Episodic migraine |
| 237. Rapoport A, Mauskop A, Diener HC, Schwalen S, Pfeil J. Long‐term migraine prevention with topiramate: open‐label extension of pivotal trials. Headache: The Journal of Head and Face Pain. 2006 Jul;46(7):1151-60. | Episodic migraine |
| 238. Lipton RB, Silberstein S, Dodick D, Cady R, Freitag F, Mathew N, Biondi DM, Ascher S, Olson WH, Hulihan J. Topiramate intervention to prevent transformation of episodic migraine: the topiramate INTREPID study. Cephalalgia. 2011 Jan;31(1):18-30. | Episodic migraine |
| 239. Ruff DD, Ford JH, Tockhorn‐Heidenreich A, Stauffer VL, Govindan S, Aurora SK, Terwindt GM, Goadsby PJ. Efficacy of Galcanezumab in patients with episodic migraine and a history of preventive treatment failure: results from two global randomized clinical trials. European Journal of Neurology. 2020 Apr;27(4):609-18. | Episodic migraine |
| 240. Sakai F, Takeshima T, Tatsuoka Y, Hirata K, Lenz R, Wang Y, Cheng S, Hirama T, Mikol DD. A randomized phase 2 study of Erenumab for the prevention of episodic migraine in Japanese adults. Headache: The Journal of Head and Face Pain. 2019 Nov;59(10):1731-42. | Episodic migraine |
| 241. Sakai F, Ozeki A, Skljarevski V. Efficacy and safety of Galcanezumab for prevention of migraine headache in Japanese patients with episodic migraine: a phase 2 randomized controlled clinical trial. Cephalalgia Reports. 2020 Jul 2;3:2515816320932573. | Episodic migraine |
| 242. Sakai F, Suzuki N, Kim BK, Tatsuoka Y, Imai N, Ning X, Ishida M, Nagano K, Iba K, Kondo H, Koga N. Efficacy and safety of Fremanezumab for episodic migraine prevention: Multicenter, randomized, double‐blind, placebo‐controlled, parallel‐group trial in Japanese and Korean patients. Headache: The Journal of Head and Face Pain. 2021 Jul;61(7):1102-11. | Episodic migraine |
| 243. Sakai F, Takeshima T, Tatsuoka Y, Hirata K, Cheng S, Numachi Y, Peng C, Xue F, Mikol DD. Long‐term efficacy and safety during open‐label Erenumab treatment in Japanese patients with episodic migraine. Headache: The Journal of Head and Face Pain. 2021 Apr;61(4):653-61. | Episodic migraine |
| 244. Smith TR, Janelidze M, Chakhava G, Cady R, Hirman J, Allan B, Pederson S, Smith J, Schaeffler B. Eptinezumab for the prevention of episodic migraine: sustained effect through 1 year of treatment in the PROMISE-1 study. Clinical therapeutics. 2020 Dec 1;42(12):2254-65. | Episodic migraine |
| 245. Spierings, E.L., Ning, X., Ramirez Campos, V., Cohen, J.M., Barash, S. and Buse, D.C., 2021. Improvements in quality of life and work productivity with up to 6 months of Fremanezumab treatment in patients with episodic and chronic migraine and documented inadequate response to 2 to 4 classes of migraine‐preventive medications in the phase 3b FOCUS study. Headache: The Journal of Head and Face Pain, 61(9), pp.1376-1386. | Episodic migraine |
| 246. Stauffer VL, Dodick DW, Zhang Q, Carter JN, Ailani J, Conley RR. Evaluation of Galcanezumab for the prevention of episodic migraine: the EVOLVE-1 randomized clinical trial. JAMA neurology. 2018 Sep 1;75(9):1080-8. | Episodic migraine |
| 247. Stauffer VL, Wang S, Voulgaropoulos M, Skljarevski V, Kovacik A, Aurora SK. Effect of Galcanezumab following treatment cessation in patients with migraine: results from 2 randomized phase 3 trials. Headache: The Journal of Head and Face Pain. 2019 Jun;59(6):834-47. | Episodic migraine |
| 248. Silberstein SD, Loder E, Forde G, Papadopoulos G, Fairclough D, Greenberg S. The impact of migraine on daily activities: effect of topiramate compared with placebo. Current medical research and opinion. 2006 Jun 1;22(6):1021-9. | Episodic migraine |
| 249. Silberstein S, Goode-Sellers S, Twomey C, Saiers J, Ascher J. Randomized, double-blind, placebo-controlled, phase II trial of gabapentin enacarbil for migraine prophylaxis. Cephalalgia. 2013 Jan;33(2):101-11. | Episodic migraine |
| 250. Tatsuoka Y, Takeshima T, Ozeki A, Matsumura T. Treatment satisfaction of Galcanezumab in Japanese patients with episodic migraine: a phase 2 randomized controlled study. Neurology and Therapy. 2021 Jun;10:265-78. | Episodic migraine |
| 251. Oakes, T.M., Kovacs, R., Rosen, N., Doty, E., Kemmer, P., Aurora, S.K. and Camporeale, A., 2020. Evaluation of cardiovascular outcomes in adult patients with episodic or chronic migraine treated with Galcanezumab: data from three phase 3, randomized, double‐blind, placebo‐controlled EVOLVE‐1, EVOLVE‐2, and REGAIN studies. Headache: The Journal of Head and Face Pain, 60(1), pp.110-123. | Episodic migraine |
| 252. Oakes TM, Skljarevski V, Zhang Q, Kielbasa W, Hodsdon ME, Detke HC, Camporeale A, Saper JR. Safety of Galcanezumab in patients with episodic migraine: a randomized placebo-controlled dose-ranging phase 2b study. Cephalalgia. 2018 May;38(6):1015-25. | Episodic migraine |
| 253. Schwedt TJ, Lipton RB, Ailani J, Silberstein SD, Tassorelli C, Guo H, Lu K, Dabruzzo B, Miceli R, Severt L, Finnegan M. Time course of efficacy of atogepant for the preventive treatment of migraine: results from the randomized, double-blind ADVANCE trial. Cephalalgia. 2022 Jan;42(1):3-11. | Episodic migraine |
| 254. Reuter U, Goadsby PJ, Lanteri-Minet M, Wen S, Hours-Zesiger P, Ferrari MD, Klatt J. Efficacy and tolerability of Erenumab in patients with episodic migraine in whom two-to-four previous preventive treatments were unsuccessful: a randomised, double-blind, placebo-controlled, phase 3b study. The Lancet. 2018 Nov 24;392(10161):2280-7. | Episodic migraine |
| 255. Reuter U, Ehrlich M, Gendolla A, Heinze A, Klatt J, Wen S, Hours-Zesiger P, Nickisch J, Sieder C, Hentschke C, Maier-Peuschel M. Erenumab versus topiramate for the prevention of migraine–a randomised, double-blind, active-controlled phase 4 trial. Cephalalgia. 2022 Feb;42(2):108-18. | Episodic migraine |
| 256. Skljarevski V, Matharu M, Millen BA, Ossipov MH, Kim BK, Yang JY. Efficacy and safety of Galcanezumab for the prevention of episodic migraine: results of the EVOLVE-2 phase 3 randomized controlled clinical trial. Cephalalgia. 2018 Jul;38(8):1442-54. | Episodic migraine |
| 257. Wang SJ, Roxas Jr AA, Saravia B, Kim BK, Chowdhury D, Riachi N, Tai ML, Tanprawate S, Ngoc TT, Zhao YJ, Mikol DD. Randomised, controlled trial of Erenumab for the prevention of episodic migraine in patients from Asia, the Middle East, and Latin America: The EMPOwER study. Cephalalgia. 2021 Nov;41(13):1285-97. | Episodic migraine |
| 258. Yang CP, Chang MH, Li TC, Hsieh CL, Hwang KL, Chang HH. Predicting prognostic factors in a randomized controlled trial of acupuncture versus topiramate treatment in patients with chronic migraine. The Clinical Journal of Pain. 2013 Nov 1;29(11):982-7. | Episodic migraine |
| 259. Fazlalizadeh H, Khamseh F, Soleimani B, Tajik A. Comparative study of topiramate versus sodium valproate in the prevention of migraine headaches. Medical Sciences Journal of Islamic Azad University. 2009;19(2). | Episodic migraine |
| 260. Brandes JL, Kudrow DB, Rothrock JF, Rupnow MF, Fairclough DL, Greenberg SJ. Assessing the ability of topiramate to improve the daily activities of patients with migraine. In Mayo Clinic Proceedings 2006 Oct 1 (Vol. 81, No. 10, pp. 1311-1319). Elsevier. | Episodic migraine |
| 261. Diamond S, Kudrow L, Stevens J, Shapiro DB. Long‐term study of propranolol in the treatment of migraine. Headache: The Journal of Head and Face Pain. 1982 Nov;22(6):268-71. | Episodic migraine |
| 262. Diener HC, Agosti R, Allais G, Bergmans P, Bussone G, Davies B, Ertas M, Lanteri-Minet M, Reuter U, Del Río MS, Schoenen J. Cessation versus continuation of 6-month migraine preventive therapy with topiramate (PROMPT): a randomised, double-blind, placebo-controlled trial. The Lancet Neurology. 2007 Dec 1;6(12):1054-62. | Episodic migraine |
| 263. Elkind AH, O’Carroll P, Blumenfeld A, DeGryse R, Dimitrova R. A series of three sequential, randomized, controlled studies of repeated treatments with botulinum toxin type A for migraine prophylaxis. The Journal of Pain. 2006 Oct 1;7(10):688-96. | Episodic migraine |
| 264. Ho TW, Connor KM, Zhang Y, Pearlman E, Koppenhaver J, Fan X, Lines C, Edvinsson L, Goadsby PJ, Michelson D. Randomized controlled trial of the CGRP receptor antagonist telcagepant for migraine prevention. Neurology. 2014 Sep 9;83(11):958-66. | Episodic migraine |
| 265. Mulleners, W.M., Kim, B.K., Láinez, M.J., Lanteri-Minet, M., Pozo-Rosich, P., Wang, S., Tockhorn-Heidenreich, A., Aurora, S.K., Nichols, R.M., Yunes-Medina, L. and Detke, H.C., 2020. Safety and efficacy of Galcanezumab in patients for whom previous migraine preventive medication from two to four categories had failed (CONQUER): a multicentre, randomised, double-blind, placebo-controlled, phase 3b trial. The Lancet Neurology, 19(10), pp.814-825. | Episodic migraine |
| 266. Ashina, M., Lanteri-Minet, M., Pozo-Rosich, P., Ettrup, A., Christoffersen, C.L., Josiassen, M.K., Phul, R. and Sperling, B., 2022. Safety and efficacy of Eptinezumab for migraine prevention in patients with two-to-four previous preventive treatment failures (DELIVER): a multi-arm, randomised, double-blind, placebo-controlled, phase 3b trial. The Lancet Neurology, 21(7), pp.597-607. | Episodic migraine |
| 267. Hu B, Li G, Li X, Wu S, Yu T, Li X, Zhao H, Jia Z, Zhuang J, Yu S. Galcanezumab in episodic migraine: the phase 3, randomized, double-blind, placebo-controlled PERSIST study. The Journal of Headache and Pain. 2022 Dec;23(1):1-1. | Episodic migraine |
| 268. Rapid resolution of migraine symptoms after initiating the preventive treatment Eptinezumab during a migraine attack: results from the randomized RELIEF trial. | Episodic migraine |
| 269. Winner PK, McAllister P, Chakhava G, Ailani J, Ettrup A, Josiassen MK, Lindsten A, Mehta L, Cady R. Effects of intravenous Eptinezumab vs placebo on headache pain and most bothersome symptom when initiated during a migraine attack: a randomized clinical trial. Jama. 2021 Jun 15;325(23):2348-56. | Episodic migraine |
| 270. Reuter U, Lucas C, Dolezil D, Hand AL, Port MD, Nichols RM, Stroud C, Tockhorn-Heidenreich A, Detke HC. Galcanezumab in patients with multiple previous migraine preventive medication category failures: results from the open-label period of the CONQUER trial. Advances in therapy. 2021 Nov;38:5465-83. | Episodic migraine |
| 271. Ferrari MD, Reuter U, Goadsby PJ, da Silva Lima GP, Mondal S, Wen S, Tenenbaum N, Pandhi S, Lanteri-Minet M, Stites T. Two-year efficacy and safety of Erenumab in participants with episodic migraine and 2–4 prior preventive treatment failures: results from the LIBERTY study. Journal of Neurology, Neurosurgery & Psychiatry. 2022 Mar 1;93(3):254-62. | Episodic migraine |

Additional file 2: Appendix 4. Baseline characteristics of the 11 RCTs presented in 51 included studies- part 1

| Author, Year  (Primary study) [Trial Name] | Author, Year (Secondary publications) | Country | Definition criteria | Treatment duration (week), and trial design | Treatment | | | | Number of participants (ITT) | Female (%) | Mean Age | Mean BMI | Mean MMD | Mean MHD | Mean MSQ-RR | Mean MSQ- PR | Mean MSQ-EF |
| --- | --- | --- | --- | --- | --- | --- | --- | --- | --- | --- | --- | --- | --- | --- | --- | --- | --- |
|  |  |  |  |  | Name | Dose | Route of administration | Frequency |  |  |  |  |  |  |  |  |  |
| Aurora 2010 ^1^  [PREEMPT1] | Dodick 2019^2^ and 2010^3^,  Silberstein 2020^4^,  Aurora 2014^5^,  Lipton 2016^6^ | 56 sites in North America | ICHD-3 | 24DB | Placebo | - | - | - | 338 | 85.8 | 42.1 | 27.3 | 19.1 | 19.8 | 38.8* | 56.1* | 43.3* |
|  |  |  |  |  | OnabotulinumtoxinA | 155 U  +40 U | IM at 39 sites | Every 12 week | 341 | 89.1 | 41.2 | 26.7 | 19.1 | 20 | 39* | 56.7* | 43.3* |
| Detke 2018 ^7^  [REGAIN] | Ruff 2019^8^,  Ford 2021^9^, Förderreuther 2018^10^, Aliani 2020^11^,  Ament 2021^12^  Ailani 2022^13^  Tobin 2022^14^  Poso-Rosich 2022^15^ | 116 centers in Argentina, Canada, Czech Republic, Germany, Israel, Italy, Mexico, Netherlands, Spain, Taiwan, UK, and USA | ICHD-3 | 12DB | Placebo | - | - | - | 558 | 87 | 41.6 | 26.5 | 19.6 | 21.5 | 38.4 | 55 | 44.2 |
|  |  |  |  |  | Galcanezumab | 120 mg | SC | Monthly | 278 | 85 | 39.7 | 26.4 | 19.4 | 21.2 | 39.3 | 55.5 | 45.3 |
|  |  |  |  |  |  | 240 mg | SC | Monthly | 277 | 82 | 41.1 | 26.7 | 19.2 | 21.4 | 38.9 | 57.1 | 45.7 |
| Diener 2010  ^16^  [PREEMPT2] | Dodick 2019^2^, and 2010^3^,  Silberstein 2020 ^4^,  Aurora 2014 ^5^,  Lipton 2016^6^ | 56 sites in North America | ICHD-3 | 24DB | Placebo | - | - | - | 358 | 84.6 | 40.9 | 27.1 | 18.7 | 19.7 | 38.8* | 56.1* | 43.3* |
|  |  |  |  |  | OnabotulinumtoxinA | 155U+40 U | IM at 39 sites | Every 12 week | 347 | 86.2 | 41 | 26.7 | 19.2 | 19.9 | 39* | 56.7* | 43.3* |
| Dodick 2019 ^17^ | - | 92 clinics/sites in USA, Australia, New Zealand, and republic of Georgia. | ICHD-3 | 12DB | Placebo | - | - | - | 121 | 90 | 37.2 | 27.6 | 16.4 | 21.1 | - | - | - |
|  |  |  |  |  | Eptinezumab | 300 mg | IV | Single dose | 121 | 81 | 37.2 | 27.3 | 16.5 | 21.1 | - | - | - |
|  |  |  |  |  |  | 100 mg | IV | Single dose | 122 | 85 | 36.7 | 27.9 | 16.9 | 21.7 | - | - | - |
|  |  |  |  |  |  | 30 mg | IV | Single dose | 122 | 91 | 35.7 | 27.1 | 16.2 | 21 | - | - | - |
|  |  |  |  |  |  | 10 mg | IV | Single dose | 130 | 87 | 36.4 | 27.4 | 16.4 | 21 | - | - | - |
| Ferrari 2019 ^18^  [FOCUS] | Spierings 2021^19^  Ferrari 2022^20^  Maassen Van Den Brink 2022^21^ | 104 sites in Europe and the USA | ICHD-3 | 12DB | Placebo | - | - | - | 279 | 84 | 46.8 | 25.3 | - | - | - | - | - |
|  |  |  |  |  | Fremanezumab | 675 mg | SC | Single dose | 276 | 83 | 45.8 | 25.1 | - | - | - | - | - |
|  |  |  |  |  |  | 675+225+225 mg | SC | Monthly | 283 | 84 | 45.9 | 25.3 | - | - | - | - | - |
| Lipton 2020 ^22^  [PROMISE2] | Diener 2021^23^,  Silberstein 2020^24^  Buse 2022^25^  Cowan 2022^26^  Marmura 2022^27^  Lipton 2022^28^  Apelian 2022^29^  Ashina 2022^30^  Vincent 2022^31^  Vincent 2022^32^  McAllister 2022^33^  Ashina 2022^34^  Starling 2023^35^ | 128  sites in 13 countries across the USA and Europe | ICHD-3 | 24DB | Placebo | - | - | - | 366 | 88.8 | 39.6 | 27 | 16.2 | 20.6 | - | - | - |
|  |  |  |  |  | Eptinezumab | 300 mg | IV | Single dose | 350 | 89.7 | 41 | 26.2 | 16.1 | 20.6 | - | - | - |
|  |  |  |  |  |  | 100 mg | IV | Single dose | 356 | 86.2 | 41 | 26.4 | 16.1 | 20.4 | - | - | - |
| Rothrock 2019 ^36^  [FORWARD] | Blumenfeld 2020^37^ | USA | ICHD-3 | 24OL | OnabotulinumtoxinA | 155U | IM at 31 sites | Every 12 week | 140 | 84 | 40.2 | 28.9 | - | 22.1 | - | - | - |
|  |  |  |  |  | Topiramate | 100 mg | Oral | Twice daily | 142 | 86 | 39.4 | 28.8 | - | 21.9 | - | - | - |
| Sakai 2021 ^38^ | Takeshima 2022^39^  Saigoh 2023^40^ | 67 institutions in Japan and Korea | ICHD-3 | 12DB | Placebo | - | - | - | 191 | 85.3 | 42.1 | 22.8 | 15.4 | 21.2 | - | - | - |
|  |  |  |  |  | Fremanezumab | 675 mg | SC | Single dose | 191 | 86.4 | 43.5 | 22.4 | 15.2 | 21.1 | - | - | - |
|  |  |  |  |  |  | 675+225+225 mg | SC | Monthly | 189 | 86.2 | 42.7 | 23.4 | 16.4 | 21.6 | - | - | - |
| Silberstein 2007 ^41^ | Silberstein 2009^42^,  Dodick 2007^43^ | 46 clinics/sites in USA | ICHD-2 | 16DB | Placebo | - | - | - | 153 | 86.9 | 38.6 | 28 | 15.1 | 20.8 | 42.4 | 62.4 | 40.6 |
|  |  |  |  |  | Topiramate | 100 mg | Oral | Twice daily | 153 | 83.7 | 37.8 | 29.1 | 15.2 | 20.4 | 43.7 | 63.5 | 43.7 |
| Silberstein  2017 ^44^  [HALO] | Winner 2019^45^,  Lipton 2020^46^,  Silberstein 2020^47^,  Blumenfeld 2021^48^ | 132 sites in nine countries across the USA and Europe | ICHD-3 | 12DB | Placebo | - | - | - | 375 | 88 | 41.4 | 26.5 | 20.3 | 16.4 | - | - | - |
|  |  |  |  |  | Fremanezumab | 675 mg | SC | Single dose | 376 | 88 | 42 | 26.6 | 20.4 | 16.2 | - | - | - |
|  |  |  |  |  |  | 675+225+225 mg | SC | Monthly | 379 | 87 | 40.6 | 26.5 | 20.3 | 16 | - | - | - |
| Tepper 2017 ^49^ | Brandes 2020^50^, Messoud Ashina 2018^51^,  Tapper 2019^52^,  Lipton 2019 ^53^  Ashina 2022^34^ | 69 headache and clinical research centers in Canada, USA and Europe | ICHD-3 | 12DB | Placebo | - | - | - | 286 | 79 | 42.1 | 26.3 | 18.2 | 21.1 | 42.8 | 60.3 | 53 |
|  |  |  |  |  | Erenumab | 70 mg | SC | Monthly | 191 | 87 | 41.4 | 26 | 17.9 | 20.5 | 44.7 | 61.9 | 53.6 |
|  |  |  |  |  |  | 140 mg | SC | Monthly | 190 | 84 | 42.9 | 26 | 17.8 | 20.7 | 45.6 | 62.9 | 56.7 |
| Yu 2022 ^54^  [DRAGON] | - | 64 sites in 9 Asian countries | ICHD-3 | 12DB | Placebo | - | - | - | 278 | 85.3 | 41.9 | 23.1 | 19.3 | 22.1 | - | - | - |
|  |  |  |  |  | Erenumab | 70 MG | SC | Monthly | 279 | 77.8 | 41.4 | 23.3 | 19.1 | 21.7 | - | - | - |

*The baseline values were not reported separately for PREEMPT1 and PREEMPT2

Abbreviations: BMI: Body Mass Index; DB: Double Blind; ICHD-2: International Classification of Headache Disorders version 2; ICHD-3: The International Classification of Headache Disorders, 3rd edition; IM: Intramuscular; ITT: Intention-to-Treat; IV, Intravenous; MHD: Monthly Headache Days; MMD: Monthly Migraine Days; MSQ-EF: Migraine Specific Quality of Life - Emotional Function; MSQ-PR: Migraine Specific Quality of Life - Preventative Role; MSQ-RR: Migraine Specific Quality of Life - Restrictive Role; OL: Open label; PREEMPT: Participants in The Phase III REsearch Evaluating Migraine Prophylaxis Therapy; SC, Subcutaneous.

Appendix 4: Baseline characteristics of the 12 RCTs presented in 55 included studies- part 2

| **First author, year/ Country** | **Purpose/ Study design and Date** | **Sample size justifications** | **Key inclusion criteria** | **Key exclusion criteria** | **Conclusion** |
| --- | --- | --- | --- | --- | --- |
| **Author, year:**  Aurora, 2010^1^  **Country:**  56 North American sites | **Purpose:**  to assess efficacy, safety and tolerability of onabotulinumtoxinA (BTA) as headache prophylaxis in adults with chronic migraine.  **Study design:**  phase 3 study, with a 24-week, double-blind, parallel-group, placebo-controlled phase followed by a 32-week, open-label phase  **Date:**  23 January 2006 to 16 July 2008 | - Planned enrollment: 650 patients. - Minimum retained sample size for headache episode frequency at week 24: n = 240 per group with SD of 5.5 - 90% power to detect 1.75 between-group difference in mean change from baseline at week 24, using alpha = 0.05   Larger sample size planned for long-term safety evaluations (>150 patients with 5 active treatment cycles) due to the study's long duration (56 weeks per patient) | - Adult (18 to 65 years) with a history of migraine according ICHD-II - Randomized patients provided diary data on >20 of 28 days during baseline. - Having >15 headache days with each day consisting of >4 hours of continuous headache and with >50% of days being migraine or probable migraine days and >4 distinct headache episodes, each lasting >4 hours. | - Previous use of botulinum toxin of any serotype or immunization to any botulinum toxin serotype - Any medical condition that puts the patient at increased risk with exposure to BTA - Diagnosis of complicated migraine, chronic tension-type headache, hypnic headache, hemicrania continua, new daily persistent headache - Use of prophylactic headache medication within 28 days prior to week -4 - Unremitting headache lasting continuously throughout the 4-week baseline period - Known or suspected Temporomandibular Disorders (TMD) - Diagnosis of fibromyalgia - Beck depression inventory score >24 at week-4 - Psychiatric problems that may have interfered with study participation | There was no between-group difference for the primary endpoint, headache episodes. However, significant reductions from baseline were observed for BTA for headache and migraine days, cumulative hours of headache-on-headache days and frequency of moderate/severe headache days, which in turn reduced the burden of illness in adults with disabling chronic migraine. |
| **Author, year:**  Detke, 2018^7^  **Country:**  Argentina, Canada, Czech Republic, Germany, Israel, Italy, Mexico, the Netherlands, Spain, Taiwan, the United Kingdom, and the United States | **Purpose:**  To evaluate the efficacy and safety of Galcanezumab, a humanized monoclonal antibody that selectively binds to calcitonin gene-related peptide, in the preventive treatment of chronic migraine.  **Study design:**  phase 3, randomized, double-blind, placebo-controlled study  **Date:**  January 2016 to March 2017 | • Target sample size: 1,140  • Assumed a 15% discontinuation rate  • Assumed an effect size of 0.30 in the last month of the 3-month treatment phase  • Aimed to provide approximately 95% power for at least 1 Galcanezumab group to separate from placebo    • Used a 1-sided 0.025 significance level. | - Adult18 to 65 years with CM as defined by ICHD-3 beta with at least 15 headache days - Migraine onset before 50 years of age. - Patients could take acute headache medication as needed throughout the trial but could take opioid- or barbiturate containing medications no more than 3 days per month, could not take oral corticosteroids, and could receive no more than 1 steroid injection during the study and only if in an emergency setting. - Patients had to wash out all migraine preventive medications except topiramate or propranolol - Patients also needed at least 1 headache-free day per month within 3 months before screening period. | - Are currently enrolled in or have participated within the last 30 days or within 5 half-lives (whichever is longer) in a clinical trial involving an investigational product. - Current use or prior exposure to galcanezumab or another calcitonin gene-related peptide (CGRP) antibody. - Known hypersensitivity to multiple drugs, monoclonal antibodies or other therapeutic proteins, or to galcanezumab. - History of persistent daily headache, cluster headache or migraine subtypes including hemiplegic (sporadic or familial) migraine, ophthalmoplegic migraine, and migraine with brainstem aura (basilar-type migraine) defined by IHS ICHD-3 beta | Both doses of Galcanezumab were superior to placebo in reducing the number of monthly MHDs. Galcanezumab appears efficacious, safe, and well tolerated for the preventive treatment of chronic migraine. |
| **Author, year:**  Diener, 2010  ^16^  **Country:**  At 66 global sites North America and 16 European | **Purpose:**  to evaluate the efficacy and safety of onabotulinumtoxinA (BTA) for prophylaxis of headaches in adults with chronic migraine  **Study design:**  a phase 3 study, with a 24-week, double-blind, placebo-controlled phase, followed by a 32-week, open-label phase.  **Date:**  7 February 2006 to 11 August 2008 | • Planned enrolment: 650 patients  • For headache day frequency, a minimum sample size of n=325 per group, SD of 6.7  • Provided >90% power to detect >1.75 between-group difference in mean change from baseline using a two-sided a=0.05 at week 24 | - Men or women aged 18–65 years with a history of migraine meeting the diagnostic criteria listed in ICHD-II section 1, migraine—with the exception of ‘‘complicated migraine’’ (i.e., hemiplegic migraine, basilar-type migraine, ophthalmoplegic migraine, migrainous infarction)—and with headache occurring on >15 days/four weeks were eligible. | - With any medical condition that might put them at increased risk if exposed to onabotulinumtoxinA (e.g., myasthenia gravis, Eaton Lambert syndrome, amyotrophic lateral sclerosis, any other significant disease that could interfere with neuromuscular function). - Diagnosis of other primary or secondary headache disorder, use of any headache prophylactic medication within 28 days of day 1 of baseline, - Beck Depression Inventory score of >24 at day 1 of baseline, temporomandibular disorder, fibromyalgia, psychiatric disorders that could interfere with study participation, or previous exposure at any time to any botulinum toxin serotype. - Prior to administration of study treatment, women of childbearing potential were required to have a negative urine pregnancy test and have been using a reliable means of Contraception | The results of PREEMPT 2 demonstrate that onabotulinumtoxinA is effective for prophylaxis of headache in adults with chronic migraine. Repeated onabotulinumtoxinA treatments were safe and well tolerated |
| **Author, year:** Dodick, 2019 ^17^  **Country:**  82 in the United States, four in Australia, and three each in New Zealand and the Republic of Georgia | **Purpose:**  To determine the safety, tolerability, and effectiveness of four dose levels of Eptinezumab and to inform the phase 3 development program.  **Study design:**  phase 2b, parallel-group, double-blind, randomized, placebo-controlled, dose-ranging clinical trial.  **Date:**  December 2014 to December 2016 | • 10% 2-sided alpha level used for each pairwise test  • 120 patients per group provided at least 90% power for each test individually  • Assumes difference in 75% responder rates > 19%  • Achieved 90% power for a pairwise comparison with 10% alpha, 120 patients per group  • Placebo rate of 25%, ALD403 rate of 43%. | - Adult 18–55 years with CM according ICHD-3b - Established at age >=35 years and history of CM of >=1 year. - >=15 headache days, of which>=8 were assessed as migraine days during baseline priod. - Use of hormonal therapy and preventive medications for headache except botulinum toxin, was allowed if the dosing has been stable for >3 months before screening, and was maintained at the same dosing level throughout the trial - The use of barbiturates or opioids for the acute treatment of CM was allowed if the dosing had been stable for 3 months before screening, and dosing did not exceed 4 days/month. - Patients with CM who were diagnosed with medication overuse headache | - Confounding pain syndromes (e.g. fibromyalgia, chronic low back pain, complex regional pain syndrome) or any pain syndrome that requires regular analgesia - Psychiatric conditions that are uncontrolled and untreated, including conditions that are not controlled for a minimum of 6 months prior to screening. - History or diagnosis of complicated migraine (ICHD-III beta version, 2013), chronic tension-type headache, hypnic headache, cluster headache, hemicrania continua, new daily persistent headache, migraine with brainstem aura, sporadic and familial hemiplegic migraine - Unable to differentiate migraine from other headaches - Subject has received botulinum toxin for migraine or for any other medical/cosmetic reasons requiring injections in the head, face, or neck within 4 months prior to screening. - Have any clinically significant concurrent medical condition | The results of this trial demonstrate that Eptinezumab appears effective and well-tolerated for the preventive treatment of chronic migraine and justifies the conduct of pivotal phase 3 trials for migraine prevention. |
| **Author, year:**  Ferrari, 2019 ^18^  **Country:**  Belgium, the  Czech Republic, Denmark, Finland, France, Germany, Italy, the Netherlands, Poland, Spain, Sweden, Switzerland,  the UK, and the USA. | **Purpose:**  To investigate the efficacy and tolerability of monthly and quarterly fremanezumab compared with placebo in patients with difficult-to-treat episodic or chronic migraine, who had documented failure to two to four pharmacological classes of migraine preventive  medications.  **Study design:**  Phase 3 FOCUS trial, randomised, double-blind, placebo-controlled, parallel-group | • Sample size of 705 participants (235 per treatment group) needed for 90% power to detect a difference of 1.8 in migraine days (assuming common SD of 6 days) at an alpha level of 0.05.  • Planned for 268 participants per treatment group for randomization, assuming a 12% discontinuation rate. | - Adult(18–70 years), had   a diagnosis of migraine with onset at or before age 50 years.   - Chronic migraine history at least 12 months before screening. - > 15 headache days per month, with at least 8 migraine days - Participants with and without overuse of acute headache medication - With failure to two to four classes of migraine preventive medications in the past 10 years. | - At the time of screening visit, participant is receiving any preventive migraine medications, regardless of the medical indication for more than 5 days and expects to continue with these medications. - Participant has received onabotulinumtoxinA for migraine or for any medical or cosmetic reasons requiring injections in the head, face, or neck during the 3 months before screening visit. - The participant has used an intervention/device (for example; scheduled nerve blocks and transcranial magnetic stimulation) for migraine during the 2 months prior to screening. - The participant uses triptans/ergots as preventive therapies for migraine. - Participant uses non-steroidal anti-inflammatory drugs (NSAIDs) as preventive therapy for migraine on nearly daily basis for other indications. Note: Low dose aspirin (for example; 81 mg) used for cardiovascular disease prevention is allowed. | Fremanezumab was effective and well tolerated in patients with difficult-to-treat migraine who had previously not responded to up to four classes of migraine preventive medications. |
| **Author, year:**  Lipton, 2020^22^  **Country:**  13 countries (United States, Spain, Ukraine, Russian  Federation, United Kingdom, Republic of Georgia, Hungary,  Italy, Slovakia, Germany, Czech Republic, Denmark, and  Belgium) | **Purpose:**  To evaluate the efficacy and safety of Eptinezumab, a humanized anti–calcitonin gene-related peptide monoclonal antibody, in the preventive treatment of chronic migraine (CM).  **Study design:**  phase 3, double-blind, randomized, placebo-controlled, parallel-group  **Date:**  30 November 2016, to 20 April 2018. | • Planned sample: 350 patients per group with at least 90% power  • Assumed treatment effect was ≥1 day and the common standard deviation (SD) was ≤4 days. | - Adults (18 to 65 years) of age (inclusive) with a diagnosis of migraine at or before 50 years of age if they had a history of CM for ≥12 months before screening, completed the headache electronic diary (eDiary) on ≥24 of the 28 days and experienced ≥15 to ≤26 headache days and ≥8 migraine days during the 28-day screening period. - Migraine preventive medication use had to be stable for ≥3 months before screening. Hormonal therapy was also permitted if it was stable and ongoing ≥3 months before screening. - Patients using barbiturates or prescription opioids ≤4 d/mo were eligible for participation if use was stable for ≥2 months before screening. - Patients with CM and medication-overuse headache with the exception of the overuse of barbiturates or opioids | - Patients using opioids or barbiturates ≥5 d/mo - With a confounding pain disorder or clinically significant pain syndromes; uncontrolled or untreated psychiatric conditions; acute or active temporomandibular disorders; history or diagnosis of a headache or migraine disorders that did not meet the ICHD-3 criteria - present or previous malignancies, any active, progressive, or unstable cardiovascular, neurologic, or autoimmune disorder; newly diagnosed or uncontrolled hypertension. - Women who were pregnant, breastfeeding, or planning to become pregnant during the study - positive for HIV, hepatitis B surface antigen, or hepatitis C - A concurrent medical condition or laboratory abnormality during the screening period or before dosing on day 0; - body mass index ≥39 kg/m2 - or recent or planned surgery requiring general anesthesia within 8 weeks before screening or during the duration of the study - botulinum toxin (any type) for migraine or for any other medical cosmetic reasons requiring injections within 4 months before screening or during the screening period - any monoclonal antibody treatment within 6 months of screening; or Eptinezumab or any monoclonal antibody targeting the CGRP pathway. | In patients with CM, Eptinezumab 100 and 300 mg was associated with a significant reduction in MMDs from the day after IV administration through week 12, was well tolerated, and demonstrated an acceptable safety profile. |
| **Author, year:** Rothrock, 2019 ^36^  **Country:**  United State | **Purpose:**  To compare effectiveness of onabotulinumtoxinA and topiramate for chronic migraine (CM) prevention.  **Study design:**  multicenter, randomized, parallel-group, post-authorization, open-label prospective study (ethodology. After 12 weeks, patients initially randomized to topiramate could cross over to onabotulinumtoxinA treatment  **Date:**  August 2014 to September 2017 | • Sample size of approximately 400 patients is needed for 90% power to detect a treatment difference of 16% (200 patients per group)  • 280 patients provide 80% power  • Enrollment target not met despite a 6-month extension to 24 months  • 80% power deemed sufficient | - Adult (18-65) had to record ≥20 diary days during 28 days baseline screening - reported ≥15 headache days. - Patients taking other preventive treatments were eligible for enrollment if the dose had been stable and well tolerated for ≥12 weeks before screening and the patient was willing to maintain a stable dose. - Patients were permitted to take prescription or over the-counter acute headache pain medication, recording use in their daily diary | - Taking opioid-containing products for acute headache treatment more than 8 days during a 28-day period - Previous treatment with botulinum toxin of any serotype for any reason - Previous treatment with topiramate - On a ketogenic diet (high in fat, low in carbohydrates) - History of acute myopia or increased intraocular pressure - Diagnosis of myasthenia gravis, Eaton-Lambert syndrome, amyotrophic lateral sclerosis or any other significant disease that might interfere with neuromuscular function - Acupuncture, transcutaneous electrical stimulation (TENS), cranial traction, dental splints for headache, or injection of anesthetics/steroids in the 4 weeks prior to screening. | Although in those few patients who were randomized to the oral medication and completed the treatment phase, topiramate was at least as efficacious as Onabotulinumtoxin A.  The high discontinuation rate associated with topiramate (the majority (51%) of patients discontinued treatment because of AEs) appears to diminish its clinical value significantly. Compared to only 4% of BTA group.  Results also demonstrate that BTA is a safe and often effective alternative for patients with CM who discontinue treatment with topiramate. |
| **Author, year:**  Sakai F, 2021^38^  **Country:**  Japan and Korea | **Purpose**:  To determine the efficacy and safety of fremanezumab administration in Japanese and Korean patients with chronic migraine (CM).  **Study design:** multicenter, randomized, double-blind, placebo-controlled, parallel-group  **Date:**  November 2017 and November 2019 | - The difference in mean change from baseline in the monthly average number of headache days of at least moderate severity between fremanezumab monthly and placebo group was 1.7 days     To detect with at least 80% power | - Patient with migraine onset at ≤50 years of age - Headache occurring on ≥15 days and fulfilling any of the following on ≥8 days: (ICHD-3 beta diagnostic criteria C and D for 1.1 Migraine without aura, criteria B and C for 1.2 Migraine with aura, Probable migraine. - Not using preventive migraine medications for migraine or other medical conditions or using no more than 1 preventive migraine medication for migraine or other medical conditions if the dose and regimen have been stable for at least 2 months prior to giving informed consent. | - The lack of efficacy of at least two of four clusters of preventive medications despite an ade-quate treatment, - Unremitting headaches with duration more than 80% of waking hours and with less than 4 days without headache per month, - Clinically significant major organ disease - Patient has received onabotulinumtoxin A for migraine or for any medical or cosmetic reason requiring injection in the head, face, or neck during the 4 months prior to giving informed consent. - Patient is using medications containing opioids or barbiturates on more than 4 days per month for the treatment of migraine or for any other reason. - Patient has used an intervention or device for migraine during the 2 months prior to giving informed consent. | Fremanezumab effectively prevents CM in Japanese and Korean patients and was well tolerated. No safety signal was detected. |
| **Author, year:** Silberstein SD,2007^41^  **Country:**  USA | **Purpose**:  To evaluate the efficacy and safety of topiramate (100 mg/day) compared with placebo for the treatment of chronic migraine  **Study design:** randomized, double-blind, placebo-controlled, parallel-group, multicenter trial  **Date:**  September 2003 to March 2005 | Nothing mentioned | - Adult subjects with at least 15headache days per 28 days with head pain for at least30 minutes, On at least half of these days, subjects have experienced migraine with or with-out aura or migrainous headache - At least 11 score of Migraine Disability Assessment (MIDAS) at visit 1 | - Previously failed more than 2 adequate trials of migraine preventive medications - Previously failed an adequate trial of topira-mate therapy due to lack of efficacy or adverseevents - History of cluster headache or basilar, ophthal-moplegic, or hemiplegic migraines - Migraine onset after age 50 - Overuse of acute migraine medication - History of hepatic disorder or nephrolithiasis - Progressive neurologic disorder other than mi-graine - Pregnant or nursing | Topiramate resulted in statistically significant improvements compared with placebo in mean monthly migraine/migraineurs and migraine headache days. Topiramate is safe and generally well tolerated. |
| **Author, year:** Silberstein SD, 2017  ^44^  **Country:**  132 sites in nine countries | **Purpose:**  To compared two Fremanezumab dose regimens with placebo for the prevention of chronic migraine.  **Study design:**  Randomized, double-blind, placebo-controlled, parallel-group trial  **Date:**  March 2016 through January 2017 | • Sample size planned: 1020 participants.   - 90% power to detect a mean (±SD) difference of 1.7±6.3 in the average number of headache days per month between the Fremanezumab-monthly group and the placebo group.   • Using a two-sided alpha level of 0.05.   - Assuming discontinuation rate of 15% | - Adult (18 to 70 years), a history of migraine according to ICHD-3 beta for at least 12 months. - ≥15 headache days with ≥8 migraine days. - The protocol allowed inclusion of up to 30% of patients using a stable dose of one migraine-preventive medication (hereafter referred to as preventive medication) for at least 2 months before the beginning of the preintervention period to continue these medications | - The use of BTA during the 4 months before screening - The use of interventions or devices for migraine, such as nerve blocks and transcranial magnetic stimulation, during the 2 months before screening - The use of opioid or barbiturate medications on more than 4 days during the pre-intervention period - and a lack of efficacy, after an adequate therapeutic trial, of at least two of four clusters of preventive medications | Fremanezumab as a preventive treatment for chronic migraine resulted in a lower frequency of headache than placebo in this 12-week trial. Injection-site reactions to the drug were common |
| **Author, year:** Tepper, 2017^49^  **Country:**  North America (Canada and the USA) and Europe  (Czech Republic, Denmark, Finland, Germany, Norway,  Poland, Sweden, and the UK | **Purpose:**  To assess the safety and  efficacy of erenumab 70 mg and 140 mg in patients with  chronic migraine.  **Study design:**  phase 2, randomised, double-blind, placebo-controlled, multicentre  **Date:**  April 3, 2014, to Dec 4, 201 | • Planned sample size: 279 for placebo and 186 for erenumab 70 mg with a treatment effect of -1.9 days and SD of 6.1  •Provided 85% power using a two-sample t-test with a two-sided significance level of 0.04  • Planned sample size: 279 for placebo and 186 for erenumab 140 mg with a treatment effect of -2.21 days and common SD of 6.1  • Provided 85% power using a two-sample t-test with a two-sided significance level of 0.01. | - History of at least 5 attacks of migraine without aura and/or migraine with visual sensory, speech and/or language, retinal or brainstem aura. - History of ≥ 15 headache days per month of which ≥ 8 headache days were assessed by the subject as migraine day. - ≥ 4 distinct headache episodes, each lasting ≥ 4 hours OR if shorter, associated with use of a triptan or ergot-derivative on the same calendar day based on the eDiary calculations. - Demonstrated at least 80% compliance with the eDiary. | - History of cluster headache or hemiplegic migraine headache - Unable to differentiate migraine from other headaches - Failed > 3 medication categories due to lack of efficacy for prophylactic treatment of migraine. - Received botulinum toxin in head or neck region within 4 months prior to screening. - Used a prohibited migraine prophylactic medication, device or procedure within 2 months prior to the start of the baseline phase | In patients with chronic migraine, Erenumab 70 mg and 140 mg reduced the number of monthly migraine days with a safety profile similar to placebo, providing evidence that Erenumab could be a potential therapy for migraine prevention. Further research is needed to understand long-term efficacy and safety of Erenumab, and the applicability of this study to real-world settings. |
| **Author, year:**  Yu 2022 ^54^  **Country:**  China, India, the Republic of Korea, Malaysia, the Philippines, Singapore, Taiwan, Thailand, and Vietnam | **Purpose:**  evaluated the efcacy and safety of erenumab in patients with chronic migraine (CM) from Asia  **Study design:**  phase 3, 12-week, randomised, double-blind, placebo-controlled  **Date:**  August 2019 and August 2021 | - Planned sample size: 550 patients. - Key assumptions in calculating the sample size were based on prior results from the erenumab global pivotal CM study. - A treatment diference in terms of change from baseline in MMD for erenumab 70  mg versus placebo was assumed at−2.0  days. - The common SD of the primary variable was estimated at 6.8. - Given a 1:1 randomisation ratio between erenumab 70 mg and placebo, it was required to enroll a total of 550 patients (including a 10% drop out rate) to achieve approximately 90% power under a two-sided 0.05 alpha level | - Adults aged 18–65 years with a history of CM with or without aura for at least 12 months before screening as defned by the International Classifcation of Headache Disorders, 3rd edition (ICHD-3). - Patients with a history of≥15 headache days/month, of which≥8 headache days met criteria as migraine days during the baseline period, and who had demonstrated at least 80% compliance with the eDiary during the baseline period. - Concomitant therapies with possible migraine prophylactic efects taken for indications other than migraine must have been administered at a stable dose within the 3 months prior to the start of the baseline period and throughout the study. | - Participants older than 50  years at migraine onset. - History of cluster or hemiplegic migraine headache; CM with continuous pain; unable to diferentiate migraine from other headaches; opioid and/or opioid-containing analgesic (for>4 days per month) or butalbital-containing analgesic (for>2 days per month) for any indication within one month before the start of or during the baseline period; - prior migraine preventive treatment failure in>3 medication categories (categories provided in Supplementary Table 2); - prior botulinum toxin A treatment in the head/ neck region within 4 months before the start of or during the baseline period, active chronic pain syndromes (such as fbromyalgia and chronic pelvic pain), or other medical conditions. - Pregnant or nursing (lactating) women, and women of childbearing potential | Treatment with erenumab 70  mg demonstrated greater reductions in MMD compared with placebo at all assessment time points, from the frst post-baseline assessment at Week 4 and sustained throughout the study. No new safety signals were observed during the DBTP compared with the previous trials. |

Additional file 2: Appendix 5. Network meta-analysis results

Table 1: Head-to-head comparisons of treatments for mean change in MHD from baseline (MDs, 95% CrI)

| Eptinezumab 300mg |  |  |  |  |  |  |  |  |
| --- | --- | --- | --- | --- | --- | --- | --- | --- |
| -0.36 (-1.47, 0.78) | Fremanezumab-M |  |  |  |  |  |  |  |
| -0.62 (-1.38, 0.16) | 0.27 (-0.89, 1.37) | Eptinezumab 100mg |  |  |  |  |  |  |
| 0.61 (-0.48, 1.65) | 0.25 (-0.82, 1.35) | -0.01 (-1.06, 1.02) | OnabotulinumtoxinA |  |  |  |  |  |
| -0.66 (-1.97, 0.67) | 0.30 (-1.02, 1.72) | -0.04 (-1.31, 1.28) | -0.05 (-1.41, 1.26) | Galcanezumab 120mg |  |  |  |  |
| -0.65 (-1.77, 0.49) | -0.30 (-1.11, 0.50) | -0.03 (-1.15, 1.10) | -0.04 (-1.11, 1.06) | 0.01 (-1.33, 1.42) | Fremanezumab-Q |  |  |  |
| -0.86 (-2.16, 0.46) | 0.51 (-0.88, 1.85) | -0.24 (-1.52, 1.06) | -0.25 (-1.56, 1.06) | 0.21 (-0.87, 1.32) | 0.21 (-1.18, 1.58) | Galcanezumab 240mg |  |  |
| -1.36 (-2.84, 0.06) | 1.01 (-0.53, 2.52) | -0.74 (-2.20, 0.67) | -0.75 (-2.18, 0.68) | 0.71 (-0.93, 2.37) | 0.71 (-0.76, 2.22) | 0.50 (-1.12, 2.18) | Topiramate 100mg |  |
| **-2.46 (-3.23, -1.69)** | **-2.11 (-2.94, -1.29)** | **-1.84 (-2.59, -1.08)** | **-1.85 (-2.59, -1.13)** | **-1.81 (-2.87, -0.66)** | **-1.81 (-2.63, -0.98)** | **-1.60 (-2.69, -0.50)** | -1.10 (-2.33, 0.17) | Placebo |


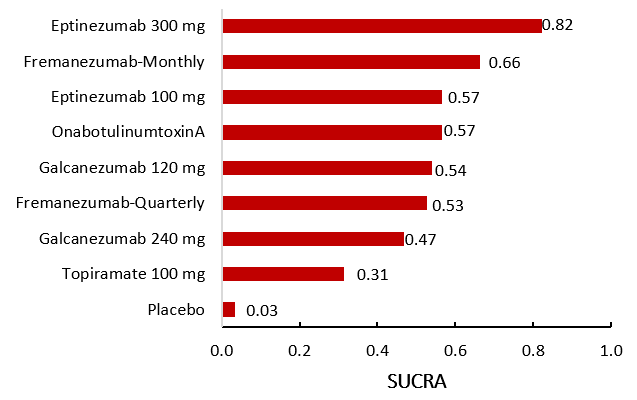


Figure 1: surface under the cumulative ranking curve (SUCRA) for mean change in MHD from baseline


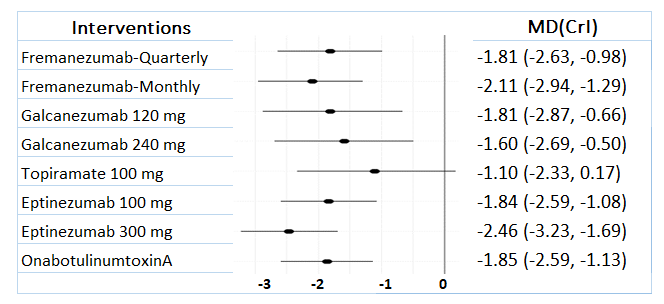


Figure 2: Forest plot for mean change in MHD from baseline (MDs, 95% CrI)

Table 2: Comparing fit of NMA and UME models for MHD

|  | Residual deviance (18 data points) | pD* | (DIC)** |
| --- | --- | --- | --- |
| NMA (consistency) Model | 16.8 | 15.1 | 31.8 |
| Unrelated Mean Effects (Inconsistency) Model | 16.7 | 16.3 | 33 |

*pD: sum of leverage, also known as the effective number of parameters and shows the model complexity.

**DIC: Deviance Information Criteria (DIC provides a measure of goodness of model fit that penalises model complexity, the lower is more fitted. More than three difference is meaningful.


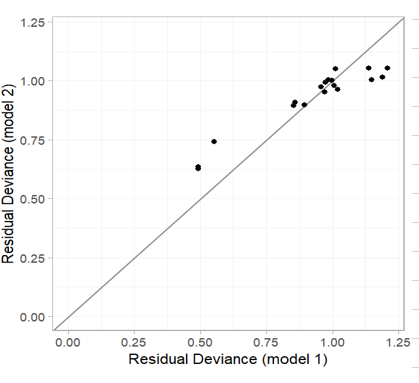


Dev-Dev plot to identify inconsistent data-points, the data points are scattered in a further distance from the bivector form the origin, they shows being source of inconsistency.

Figure 3: Global Consistency Test for mean change in MHD from baseline (Unrelated Mean Effects (Inconsistency) Model and Fixed NMA model)

Table 3: Head-to-head comparisons of treatments for mean change in MMD from baseline (MDs, 95% CrI)

| Fremanezumab-M |  |  |  |  |  |  |  |  |  |  |
| --- | --- | --- | --- | --- | --- | --- | --- | --- | --- | --- |
| 0.17 (-0.99, 1.36) | Eptinezumab 300mg |  |  |  |  |  |  |  |  |  |
| -0.46 (-1.08, 0.18) | -0.29 (-1.47, 0.93) | Fremanezumab-Q |  |  |  |  |  |  |  |  |
| 0.50 (-0.75, 1.71) | -0.33 (-1.74, 0.15) | 0.04 (-1.20, 1.23) | Erenumab 140mg |  |  |  |  |  |  |  |
| 0.66 (-0.61, 1.92) | -0.48 (-1.97, 0.98) | 0.20 (-1.04, 1.46) | -0.16 (-1.66, 1.37) | Galcanezumab 120mg |  |  |  |  |  |  |
| 0.65 (-0.40, 1.68) | -0.48 (-1.78, 0.85) | 0.19 (-0.89, 1.28) | -0.16 (-1.25, 0.89) | 0 (-1.41, 1.38) | Erenumab 70mg |  |  |  |  |  |
| 0.68 (-0.45, 1.79) | -0.51 (-1.49, 0.47) | 0.22 (-0.88, 1.37) | 0.18 (-1.19, 1.53) | 0.02 (-1.40, 1.47) | 0.03 (-1.23, 1.30) | Eptinezumab 100mg |  |  |  |  |
| 0.80 (-0.14, 1.75) | 0.63 (-0.57, 1.85) | 0.34 (-0.59, 1.27) | 0.30 (-0.99, 1.60) | 0.14 (-1.21, 1.50) | 0.15 (-0.96, 1.25) | 0.12 (-1.06, 1.28) | BTA |  |  |  |
| 0.86 (-0.37, 2.07) | -0.69 (-2.17, 0.76) | 0.40 (-0.87, 1.64) | -0.36 (-1.94, 1.13) | 0.20 (-0.83, 1.27) | -0.20 (-1.62, 1.17) | -0.18 (-1.61, 1.23) | -0.06 (-1.37, 1.26) | Galcanezumab 240mg |  |  |
| 1.27 (-0.19, 2.76) | -1.1 (-2.79, 0.52) | 0.82 (-0.64, 2.35) | 0.78 (-0.97, 2.48) | 0.62 (-1.18, 2.39) | 0.62 (-0.91, 2.22) | -0.06 (-2.22, 1.02) | -0.47 (-2.02, 1.04) | 0.42 (-1.37, 2.22) | Topiramate 100mg |  |
| **-2.77 (-3.36, -2.17)** | **-2.60 ( -3.61, -1.59)** | **-2.31 (-2.95, -1.67)** | **-2.27 (-3.31, -1.24)** | **-2.11 (-3.22, -0.95)** | **-2.11 (-3.01, -1.26)** | **-2.09 (-3.01, -1.17)** | **-1.97 (-2.69, -1.26)** | **-1.91 (-3.02, -0.85)** | **-1.49 (-2.82, -0.15)** | Placebo |


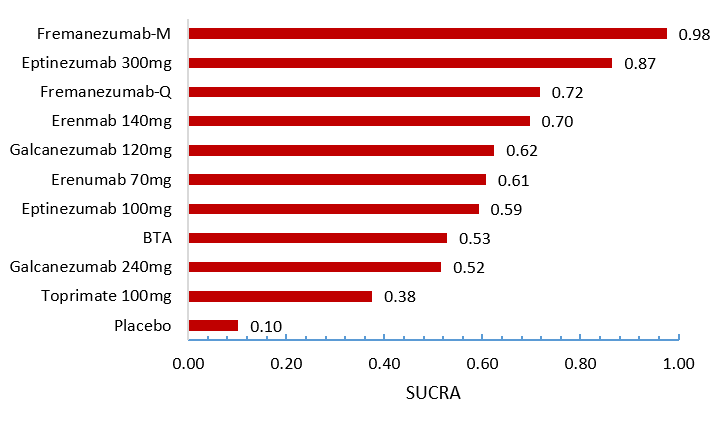
**Figure 4: surface under the cumulative ranking curve (SUCRA) for mean change in MMD from baseline**


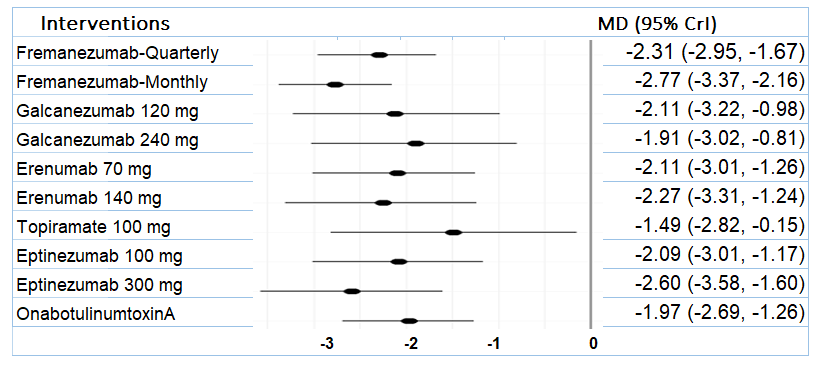


Figure 5: Forest plot for mean change in MMD from baseline (MDs, 95% CrI)

Table 4: Comparing fit of NMA and UME models for MMD

|  | Residual deviance (29 data points) | pD* | (DIC)** |
| --- | --- | --- | --- |
| NMA (consistency) Model | 34.1 | 20.9 | 55 |
| Unrelated Mean Effects (Inconsistency) Model | 34 | 20.8 | 54.8 |

*pD: sum of leverage, also known as the effective number of parameters and shows the model complexity.

**DIC: Deviance Information Criteria (DIC provides a measure of goodness of model fit that penalises model complexity, the lower is more fitted. More than three difference is meaningful.


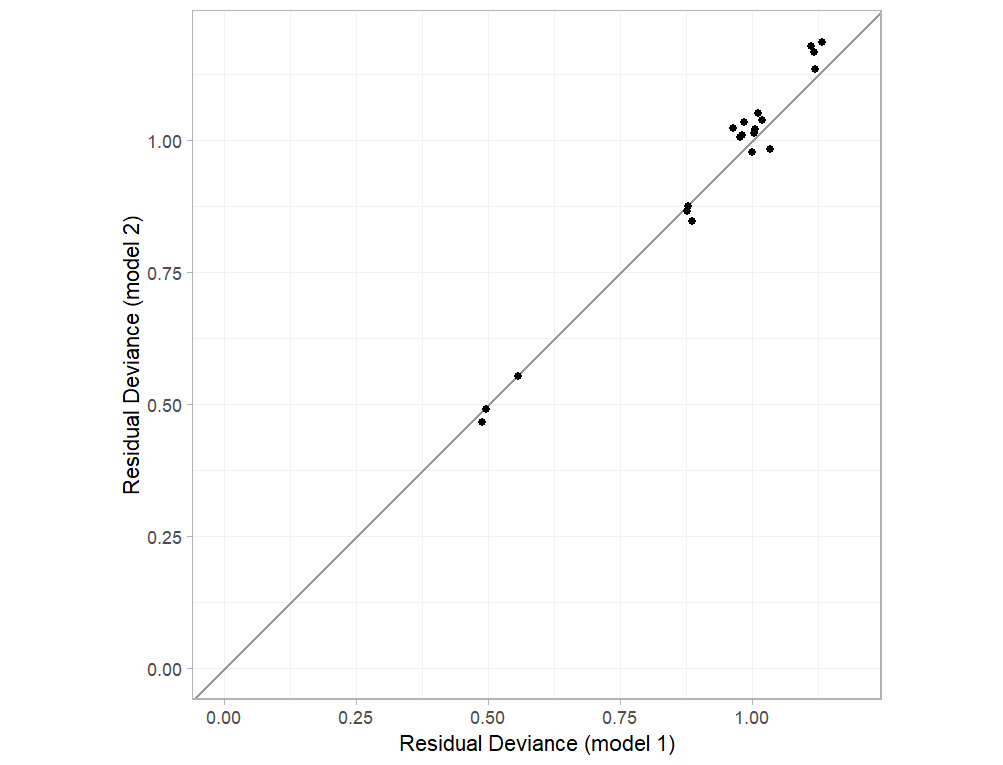


Dev-Dev plot to identify inconsistent data-points, the data points are scattered in a further distance from the bivector form the origin, they shows being source of inconsistency.

Figure 6: Global Consistency Test for mean change in MMD from baseline (Unrelated Mean Effects (Inconsistency) Model and Fixed NMA model)

|   **B**  **A**  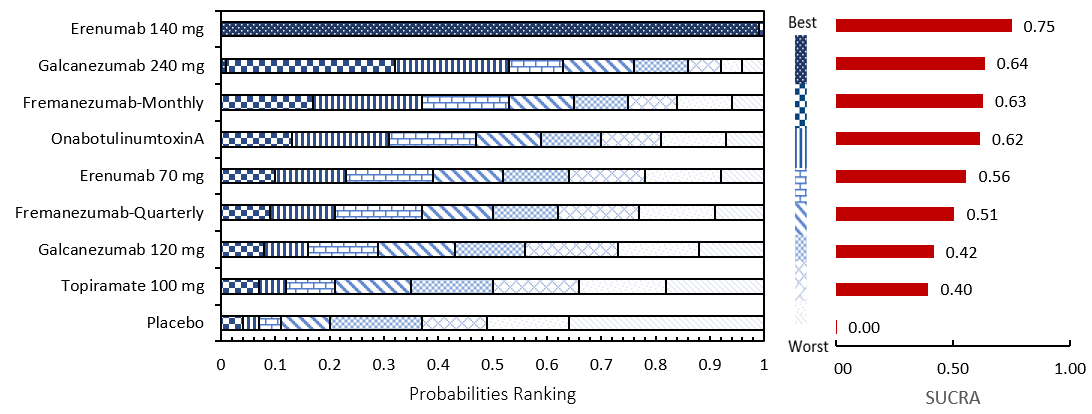 |
| --- |
| A - The forest plot shows the mean difference (MDs) and 95% credible interval (CrI) compared with placebo as reference treatment. MDs lower than 0 indicate favoured results for the intervention.  B - Ranking probabilities graph (hatching blue bars) of each treatment and red bars are the SUCRA values for each treatment. The probabilities ranking ranged from 0 to 1. Due to rounding in R software these do not equate to one.  Abbreviations: MSQ-RR, Migraine Specific Quality of Life - Restrictive Role; SUCRA, surface under the cumulative ranking curve. |

Figure 7: Summary of the change in MSQ-RR from baseline

Table 5: Head-to-head comparisons of treatments for mean change in MSQ-RR from baseline

| Erenumab 140mg |  |  |  |  |  |  |  |  |
| --- | --- | --- | --- | --- | --- | --- | --- | --- |
| 1.02 (-4.32, 6.49) | Galcanezumab 240mg |  |  |  |  |  |  |  |
| 1.02 (-4.15, 6.29) | 0.01 (-4.58, 4.60) | Fremanezumab-M |  |  |  |  |  |  |
| -0.96 (-6.59, 4.94) | 0.06 (-4.79, 4.89) | 0.06(-4.78, 4.75) | BTA |  |  |  |  |  |
| 1.41 (-3.01, 5.86) | -0.39 (-5.47, 4.59) | -0.39 (-5.42, 4.53) | 0.45 (-5.09, 5.81) | Erenumab 70mg |  |  |  |  |
| 1.71 (-3.49, 6.85) | 0.68 (-3.68, 5.07) | 0.69 (-2.38, 3.66) | 0.75 (-4.04, 5.37) | 0.30 (-4.68, 5.26) | Fremanezumab-Q |  |  |  |
| 2.33 (-2.82, 7.76) | 1.31 (-2.02, 4.76) | -1.31 (-5.72, 3.21) | 1.37 (-3.53, 6.16) | 0.92 (-4.06, 5.88) | -0.62 (-4.95, 3.81) | Galcanezumab 120mg |  |  |
| -2.95 (-10.46, 4.43) | -1.93 (-8.71, 5.06) | -1.93 (-8.75,4.92) | 1.99 (-5.23, 9.11) | -1.54 (-9.02, 5.87) | -1.24 (-7.91, 5.52) | -0.62 (-7.51, 6.17) | Topiramate 100mg |  |
| **7.28 (3.05, 11.65)** | **6.26(2.96, 9.49)** | **6.27 (3.09, 9.28)** | **6.32 (2.51, 9.95)** | **5.87 (2.03, 9.87)** | **5.58 (2.68, 8.54)** | **4.95(1.91, 8.08)** | 4.33 (-1.88, 10.50) | Placebo |

Mean differences more than 0 favour the column-defining treatment; Credible Intervals not including 0 are highlighted in bold. Abbreviations: Fremanezumab-M, Fremanezumab monthly; Fremanezumab-Q, Fremanezumab quarterly

Table 6: The model fit result for mean change in MSQ-RR from baseline

|  | Residual deviance (13 data points) | pD* | DIC** |
| --- | --- | --- | --- |
| Fixed model | 12.9 | 12.9 | 25.8 |
| Random model | 13.1 | 13.1 | 26.3 |

*pD: sum of leverage, also known as the effective number of parameters and shows the model complexity.

**DIC: Deviance Information Criteria (DIC provides a measure of goodness of model fit that penalises model complexity, the lower is more fitted. More than three difference is meaningful.

Table 7: Comparing fit of NMA and UME models for MSQ-RR

|  | **Residual deviance (13 data points)** | **pD*** | **(DIC)**** |
| --- | --- | --- | --- |
| NMA (consistency) Model | 12.9 | 12.9 | 25.8 |
| Unrelated Mean Effects (Inconsistency) Model | 13 | 13 | 26 |

*pD: sum of leverage, also known as the effective number of parameters and shows the model complexity. **DIC: Deviance Information Criteria (DIC provides a measure of goodness of model fit that penalises model complexity, the lower is more fitted. More than three difference is meaningful.


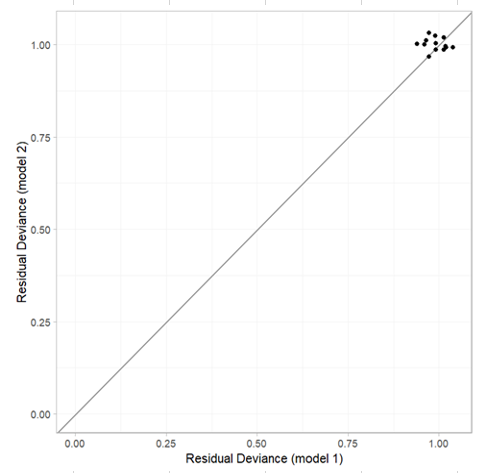


Dev-Dev plot to identify inconsistent data-points, the data points are scattered in a further distance from the bivector form the origin, they shows being source of inconsistency.

**Figure 8: Global Consistency Test for mean change in MSQ-RR from baseline (Unrelated Mean Effects (Inconsistency) Model and Fixed NMA model)**

| **A**  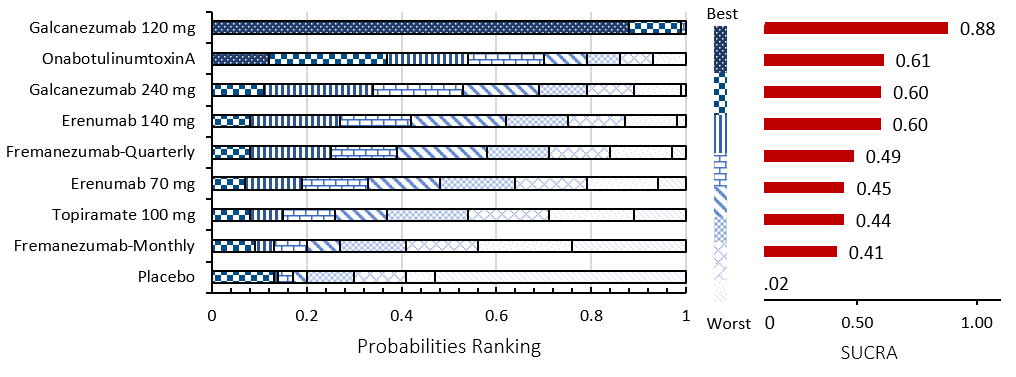  **B** |
| --- |
| A - The forest plot shows the mean difference (MDs) and 95% credible interval (CrI) compared with placebo as reference treatment. MDs lower than 0 indicate favoured results for the intervention.  B - Ranking probabilities graph (hatching blue bars) of each treatment and red bars are the SUCRA values for each treatment. The probabilities ranking ranged from 0 to 1. Due to rounding in R software these do not equate to one.  Abbreviations: MSQ-PR, Migraine Specific Quality of Life - Preventative Role; SUCRA, surface under the cumulative ranking curve. |

Figure 9: Summary of the change in MSQ-PR from baseline

Table 8: Head-to-head comparisons of treatments for mean change in MSQ-PR from baseline

| Galcanezumab 120mg |  |  |  |  |  |  |  |  |
| --- | --- | --- | --- | --- | --- | --- | --- | --- |
| -1.95 (-6.41, 2.37) | BTA |  |  |  |  |  |  |  |
| -1.89 (-5.15, 1.44) | -0.07 (-4.56, 4.41) | Galcanezumab 240mg |  |  |  |  |  |  |
| -2.04 (-6.59, 2.51) | 0.09 (-4.32, 4.60) | -0.15 (-4.26, 4.30) | Erenumab 140mg |  |  |  |  |  |
| 2.68 (-1.42, 6.73) | 0.73 (-3.30, 4.55) | 0.79 (-3.28, 4.87) | 0.64 (-3.44, 4.63) | Fremanezumab-Q |  |  |  |  |
| -2.88 (-7.34, 1.49) | 0.93 (-3.51, 5.37) | -0.99 (-5.57, 3.64) | 0.84 (-2.69, 4.29) | -0.20 (-4.47, 3.81) | Erenumab 70mg |  |  |  |
| -3.19 (-10.04, 3.51) | 1.24 (-5.50, 8.32) | -1.30 (-8.06, 5.64) | -1.15 (-8.18, 5.69) | -0.51 (-7.05, 6.02) | -0.31 (-7.11, 6.38) | Topiramate 100mg |  |  |
| 3.08 (-1.06, 7.14) | 1.12 (-2.71, 5.08) | 1.19 (-2.87, 5.17) | 1.03 (-3.08, 5.21) | -0.39 (-2.81, 1.96) | 0.20 (-3.88, 4.30) | -0.11 (-6.76, 6.27) | Fremanezumab-M |  |
| **6.97 (3.79, 10.24)** | **5.01 (1.99, 8.01)** | **5.08 (1.84, 8.35)** | **4.93 (1.70, 8.20)** | **4.29 (1.90, 6.81)** | **4.09 (0.76, 7.31)** | 3.78 (-2.37, 9.80) | **3.89 (1.39, 6.41)** | Placebo |

Mean differences more than 0 favour the column-defining treatment; Credible Intervals not including 0 are highlighted in bold.

Abbreviations: Fremanezumab-M, Fremanezumab Monthly; Fremanezumab-Q, Fremanezumab Quarterly.

Table 9: The model fit result for mean change in MSQ-PR from baseline

|  | Residual deviance (13 data points) | pD* | DIC** |
| --- | --- | --- | --- |
| Fixed model | 13.1 | 13.1 | 26.2 |
| Random model | 12.8 | 12.8 | 25.7 |

*pD: sum of leverage, also known as the effective number of parameters and shows the model complexity.

**DIC: Deviance Information Criteria (DIC provides a measure of goodness of model fit that penalises model complexity, the lower is more fitted. More than three difference is meaningful.

Table 10: Comparing fit of NMA and UME models for MSQ-PR

|  | **Residual deviance (13 data points)** | **pD*** | **(DIC)**** |
| --- | --- | --- | --- |
| NMA (consistency) Model | 13.1 | 13.1 | 26.2 |
| Unrelated Mean Effects (Inconsistency) Model | 12.9 | 12.9 | 25.8 |

*pD: sum of leverage, also known as the effective number of parameters and shows the model complexity.

**DIC: Deviance Information Criteria (DIC provides a measure of goodness of model fit that penalises model complexity, the lower is more fitted. More than three difference is meaningful.


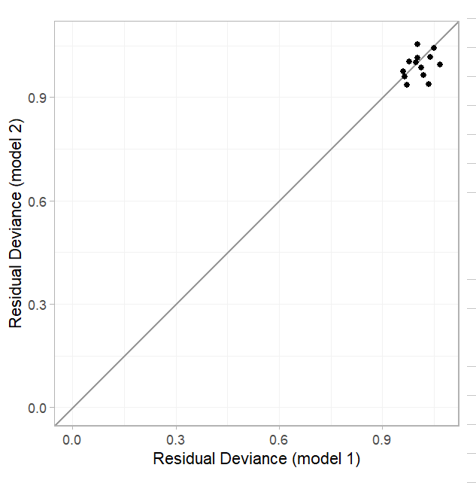


Dev-Dev plot to identify inconsistent data-points, the data points are scattered in a further distance from the bivector form the origin, they shows being source of inconsistency.

Figure 10: Global Consistency Test for mean change in MSQ-PR from baseline (Unrelated Mean Effects (Inconsistency) Model and Fixed NMA model)

| **A**  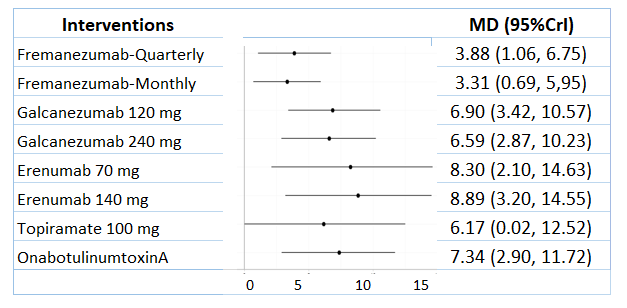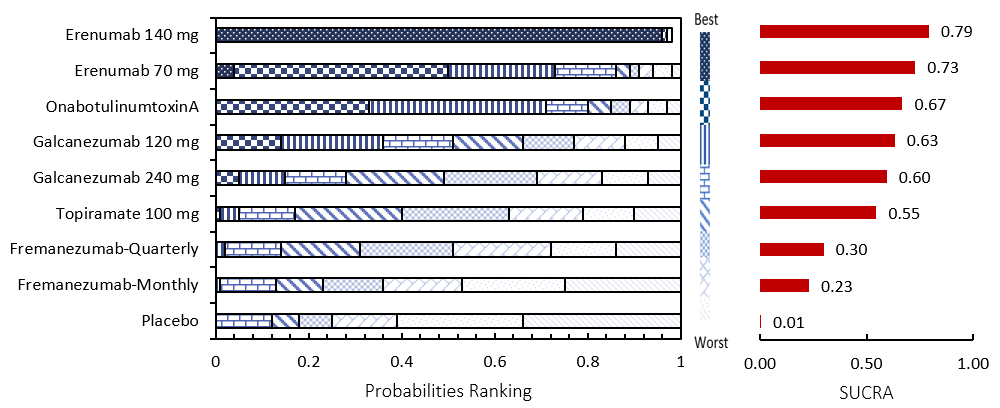  **B** |
| --- |
| A - The forest plot shows the mean difference (MDs) and 95% credible interval (CrI) compared with placebo as reference treatment. MDs lower than 0 indicate favoured results for the intervention.  B - Ranking probabilities graph (hatching blue bars) of each treatment and red bars are the SUCRA values for each treatment. The probabilities ranking ranged from 0 to 1. Due to rounding in R software these do not equate to one.  Abbreviations: MSQ-EF, Migraine Specific Quality of Life - Emotional Function; SUCRA, surface under the cumulative ranking curve. |

**Figure 11: Summary of the Change in MSQ-EF from baseline**

Table 11: The model fit result for mean change in MSQ-EF from baseline

|  | Residual deviance (13 data points) | pD* | DIC** |
| --- | --- | --- | --- |
| Fixed model | 13 | 13 | 26 |
| Random model | 12.8 | 12.8 | 25.6 |

*pD: sum of leverage, also known as the effective number of parameters and shows the model complexity.

**DIC: Deviance Information Criteria (DIC provides a measure of goodness of model fit that penalises model complexity, the lower is more fitted. More than three difference is meaningful.

Table 12: Comparing fit of NMA and UME models for MSQ-EF

|  | **Residual deviance (13 data points)** | **pD*** | **(DIC)**** |
| --- | --- | --- | --- |
| NMA (consistency) Model | 13 | 13 | 26 |
| Unrelated Mean Effects (Inconsistency) Model | 13 | 13 | 26 |

*pD: sum of leverage, also known as the effective number of parameters and shows the model complexity.

**DIC: Deviance Information Criteria (DIC provides a measure of goodness of model fit that penalises model complexity, the lower is more fitted. More than three difference is meaningful.

Table 13: Head-to-head comparisons of treatments for mean change in MSQ-EF from baseline

| Erenumab 140mg |  |  |  |  |  |  |  |  |
| --- | --- | --- | --- | --- | --- | --- | --- | --- |
| 0.59 (-5.82, 7.14) | Erenumab 70mg |  |  |  |  |  |  |  |
| -1.55(-8.60, 5.55) | -0.97 (-8.49, 6.75) | BTA |  |  |  |  |  |  |
| 1.99 (-4.84, 8.74) | 1.41 (-5.89, 8.58) | 0.44 (-5.48, 6.13) | Galcanezumab 120mg |  |  |  |  |  |
| 2.30 (-4.41, 9.12) | 1.71 (-5.70, 9.30) | 0.74 (-4.96, 6.49) | -0.31 (-4.08, 3.49) | Galcanezumab 240mg |  |  |  |  |
| -2.72 (-10.92, 5.49) | -2.13 (-11.04,6.98) | 1.17 (-6.56, 8.78) | -0.73 (-7.79, 6.61) | -0.42(-7.52, 6.94) | Topiramate 100mg |  |  |  |
| 5.01 (-1.35, 11.35) | 4.42 (-2.31 11.35) | 3.46 (-1.88, 8.79) | 3.02 (-1.73, 7.69) | 2.71 (-2.00, 7.39) | 2.29 (-4.60, 9.13 | Fremanezumab-Q |  |  |
| 5.58 (-0.77, 11.65) | 4.99 (-3.48, 11.74) | 4.03 (-1.10, 9.18) | 3.59 (-1.09, 8.25) | 3.28 (-1.31, 7.72) | 2.86 (-3.78, 9.31) | -0.57 (-3.31, 2.21) | Fremanezumab-M |  |
| **8.89 (3.20, 14.55)** | **8.30 (2.10, 14.63)** | **7.34 (2.90, 11.72)** | **6.90 (3.42, 10.57)** | **6.59 (2.87, 10.23)** | **6.17 (0.02, 12.52)** | **3.88 (1.06, 6.75** | **3.31 (0.69, 5,95)** | Placebo |

Mean differences more than 0 favour the column-defining treatment; Credible Intervals not including 0 are highlighted in bold.Abbreviations: Fremanezumab-M, Fremanezumab Monthly; Fremanezumab-Q, Fremanezumab Quarterly.


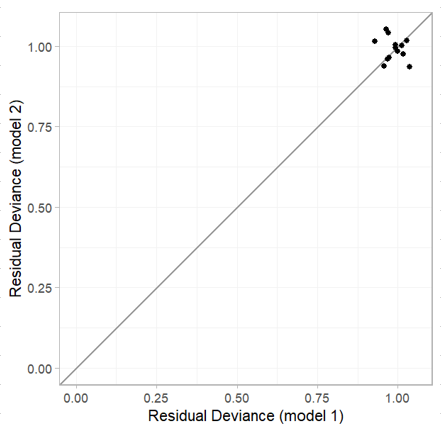


Dev-Dev plot to identify inconsistent data-points, the data points are scattered in a further distance from the bivector form the origin, they shows being source of inconsistency.

**Figure 12: Global Consistency Test for mean change in MSQ-EF from baseline (Unrelated Mean Effects (Inconsistency) Model and Fixed NMA model)**

Table 14: Head-to-head comparisons of treatments for mean change in HIT-6 from baseline (MDs, 95% CrI)

| Eptinezumab 300mg |  |  |  |  |  |  |  |
| --- | --- | --- | --- | --- | --- | --- | --- |
| -0.72 (-2.56, 1.07) | Erenumab 140mg |  |  |  |  |  |  |
| -0.72 (-2.60, 1.19) | 0 (-1.55, 1.61) | Erenumab 70mg |  |  |  |  |  |
| 1.14 (-0.53, 2.80) | 0.42 (-1.48, 2.32) | 0.42 (-1.49, 2.38) | BTA |  |  |  |  |
| -1.25 (-2.62, 0.2) | -0.53 (-2.16, 1.2) | -0.54 (-2.21, 1.18) | -0.12 (-1.62, 1.36) | Fremanezumab-M |  |  |  |
| -1.44 (-2.83, 0.03) | -0.73 (-2.43, 1.04) | -0.73 (-2.42, 1.06) | -0.31 (-1.8, 1.2) | -0.19 (-1.2, 0.65) | Fremanezumab-Q |  |  |
| **-1.66 (-2.75, -0.58)** | 0.95 (-0.82, 2.68) | 0.95 (-0.81, 2.70) | -0.53 (-2.01, 1.02) | 0.41 (-0.81, 1.64) | -1.44 (-2.83, 0.03) | Eptinezumab 100mg |  |
| **-3.23 (-4.33, -2.11)** | **-2.51 (-3.94, -1.06)** | **-2.52 (-4.02, -1.02)** | **-2.10 (-3.34, -0.90)** | **-1.98 (-2.77, -1.16)** | **-1.79 (-2.66, -0.9)** | **-1.57 (-2.5, -0.064)** | Placebo |


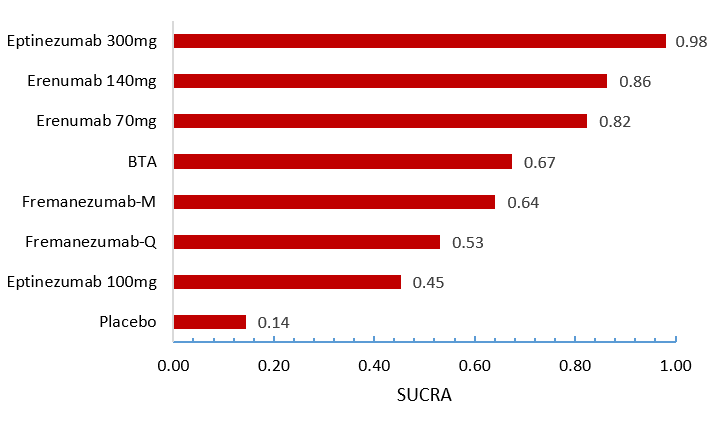


Figure 13: surface under the cumulative ranking curve (SUCRA) for mean change in HIT-6 from baseline


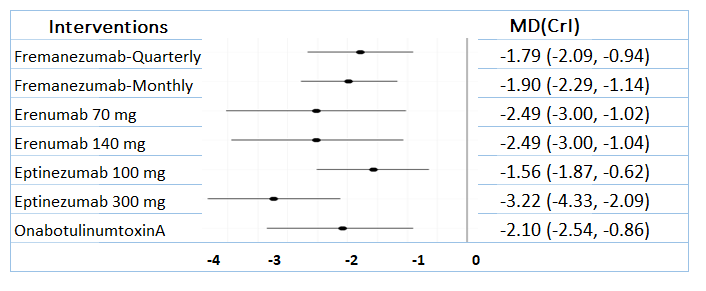


Figure 14: Forest plot for mean change in HIT-6 from baseline

Table 15: The model fit result for mean change in HIT-6 from baseline

|  | Residual deviance (17 data points) | pD* | DIC** |
| --- | --- | --- | --- |
| Fixed model | 16.2 | 13 | 29.2 |
| Random model | 16.1 | 12.9 | 29 |

*pD: sum of leverage, also known as the effective number of parameters and shows the model complexity.

**DIC: Deviance Information Criteria (DIC provides a measure of goodness of model fit that penalises model complexity, the lower is more fitted. More than three difference is meaningful.

Table 16: Comparing fit of NMA and UME models for HIT-6

|  | **Residual deviance (17 data points)** | **pD*** | **(DIC)**** |
| --- | --- | --- | --- |
| NMA (consistency) Model | 16.2 | 13 | 29.2 |
| Unrelated Mean Effects (Inconsistency) Model | 16.3 | 13.1 | 29.4 |

*pD: sum of leverage, also known as the effective number of parameters and shows the model complexity.

**DIC: Deviance Information Criteria (DIC provides a measure of goodness of model fit that penalises model complexity, the lower is more fitted. More than three difference is meaningful.


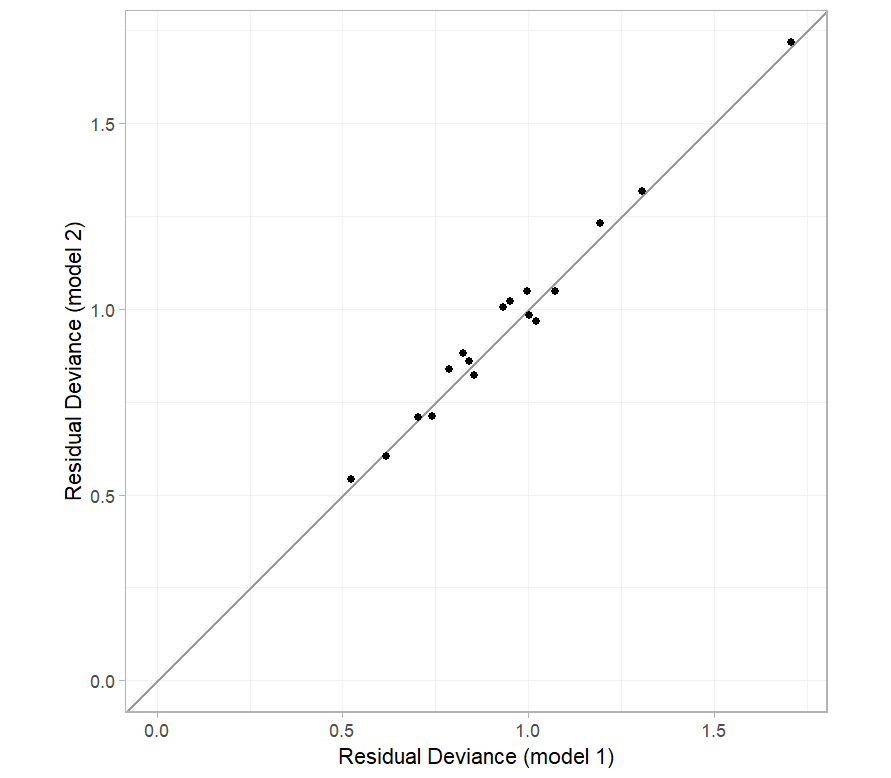


Dev-Dev plot to identify inconsistent data-points, the data points are scattered in a further distance from the bivector form the origin, they shows being source of inconsistency.

**Figure 15: Global Consistency Test for mean change in MSQ-EF from baseline (Unrelated Mean Effects (Inconsistency) Model and Fixed NMA model)**

| 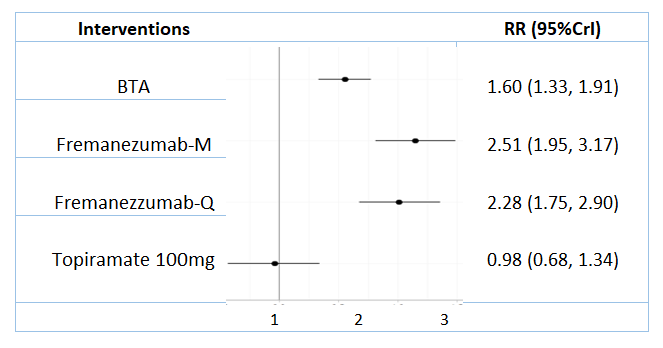  **B**  **A**  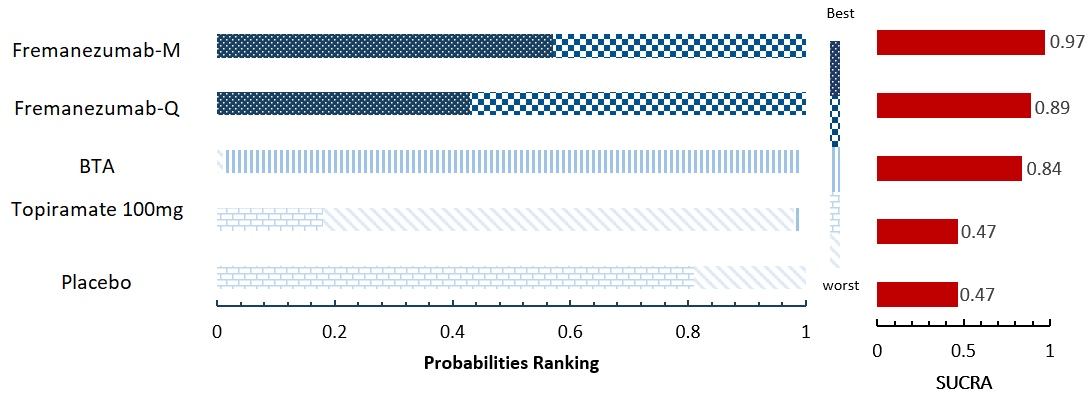 |
| --- |
| A - The forest plot shows the risk ratios (RRs) and 95% credible interval (CrI) compared with placebo as reference treatment. RRs larger than 1 indicate favoured results for the intervention.  B - Ranking probabilities graph (hatching blue bars) of each treatment and red bars are the SUCRA values for each treatment. The probabilities ranking ranged from 0 to 1. Due to rounding in R software these do not equate to one. |

Figure 16: Summary of 50% or more reduction in headache days

Table 17: The model fit result for 50% or more reduction in headache days

|  | **Residual deviance (9 data points)** | **pD*** | **DIC**** |
| --- | --- | --- | --- |
| Fixed model | 10.5 | 8 | 18.5 |
| Random model | 10 | 8.1 | 18.1 |

*pD: sum of leverage, also known as the effective number of parameters and shows the model complexity.

**DIC: Deviance Information Criteria (DIC) provides a measure of goodness of model fit that penalises model complexity, the lower is more fitted. More than three difference is meaningful.

Table 18: Comparing fit of NMA and UME models for 50% or more reduction in headache days

|  | Residual deviance (9 data points) | pD* | (DIC)** |
| --- | --- | --- | --- |
| NMA (consistency) Model | 9.1 | 9.1 | 18.2 |
| Unrelated Mean Effects (Inconsistency) Model | 10.5 | 8 | 18.5 |

*pD: sum of leverage, also known as the effective number of parameters and shows the model complexity.

**DIC: Deviance Information Criteria (DIC provides a measure of goodness of model fit that penalises model complexity, the lower is more fitted. More than three difference is meaningful


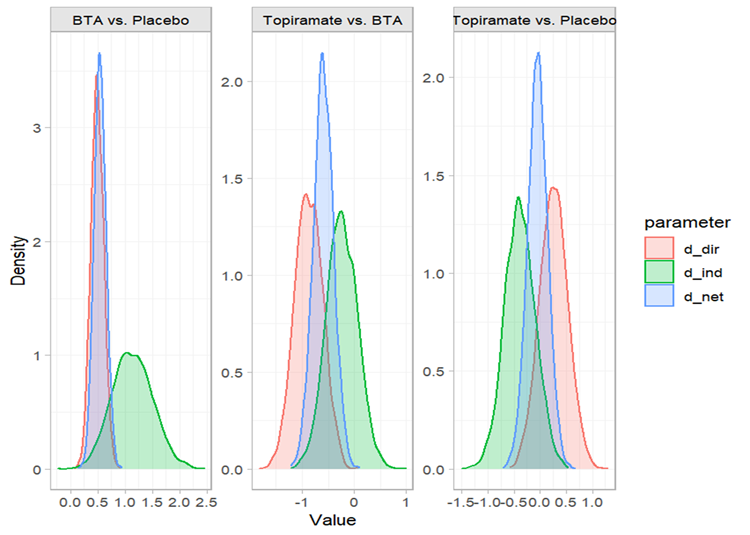


Figure 17: Local Consistency test for 50% or more reduction in headache days

| **B**  **A**  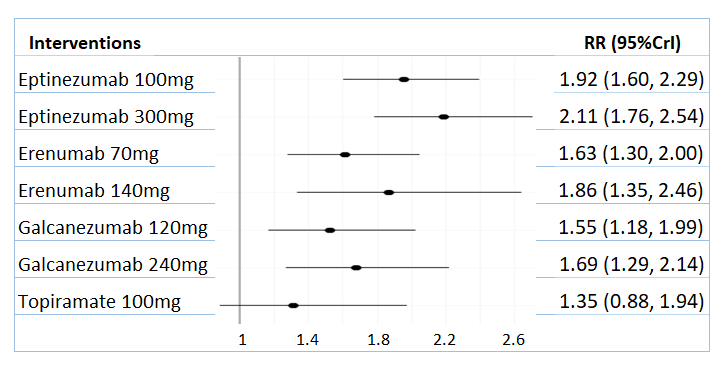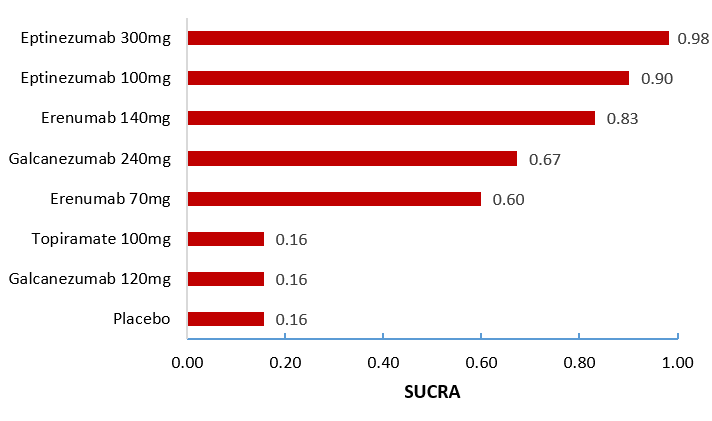 |
| --- |
| A - The forest plot shows the risk ratios (RRs) and 95% credible interval (CrI) compared with placebo as reference treatment. RRs larger than 1 indicate favoured results for the intervention.  B - The SUCRA values for each treatment. |

Figure 18: Summary of 50% or more reduction in migraine days

Table 19: The model fit result for 50% or more reduction in migraine days

|  | **Residual deviance (19 data points)** | **pD*** | **DIC**** |
| --- | --- | --- | --- |
| Fixed model | 18.1 | 14 | 32.1 |
| Random model | 17.9 | 14.6 | 32.6 |

*pD: sum of leverage, also known as the effective number of parameters and shows the model complexity.

**DIC: Deviance Information Criteria (DIC) provides a measure of goodness of model fit that penalises model complexity, the lower is more fitted. More than three difference is meaningful.

Table 20: Comparing fit of NMA and UME models for 50% or more reduction in migraine days

|  | Residual deviance (19 data points) | pD* | (DIC)** |
| --- | --- | --- | --- |
| NMA (consistency) Model | 18.1 | 14 | 32.1 |
| Unrelated Mean Effects (Inconsistency) Model | 18.2 | 14.5 | 32.7 |

*pD: sum of leverage, also known as the effective number of parameters and shows the model complexity.

**Additional file 2: Appendix 6. Risk of bias assessment result**

| 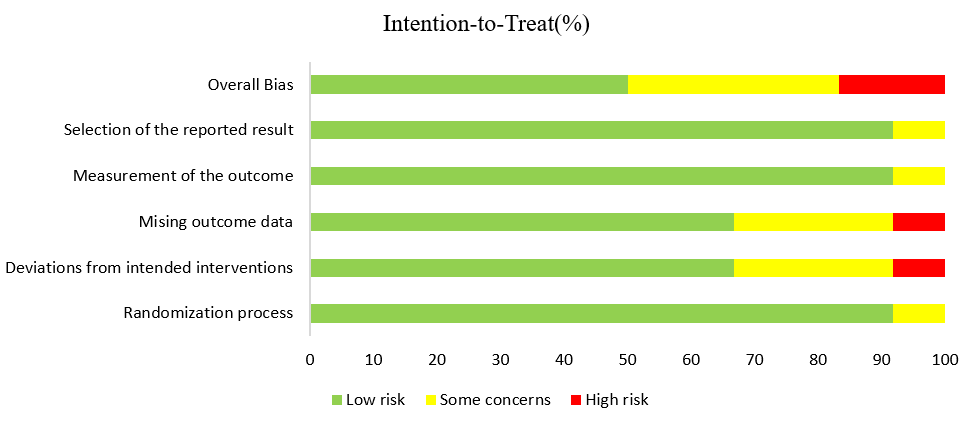 |
| --- |
| 1. **Summary of risk of bias assessment** |
| 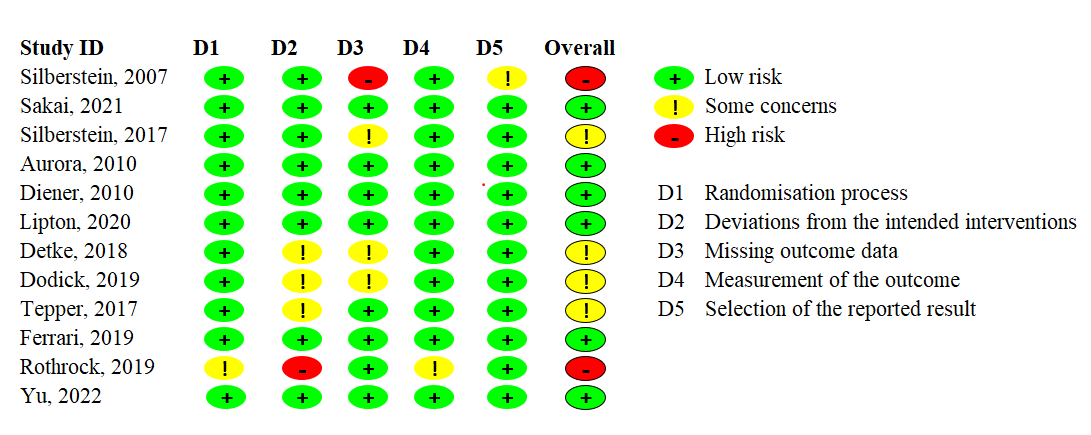 |
| 1. **Traffic lights for the risk of bias for each included study** |

Figure 1: Risk of bias assessment result

**Additional file 2: Appendix 7.** The Grading **of Recommendations Assessment, Development and Evaluation (GRADE) approach for rating the quality of estimates of treatment effect size.**

- **Study Limitations:** Determining risk of bias; the rating was downgraded by one level when the studies with high risk of bias in at least one domain contributed to the comparisons.
- **Imprecision**: Focusing on width of the confidence interval; the rating was downgraded by one level when the null value lies within the 95% Credible IntervaIs. The null value is zero for continuous outcomes.
- **Inconsistency:** Judging the extent of heterogeneity; tau-squared values were very low with little suggestion of heterogeneity for all outcomes. And, also evaluating the inconsistent loops of evidence which in our six NMA model, there was no evidence of close loops to assess local inconsistency. Global inconsistency test showed a consistent evidence of data points.
- **Indirectness:** Evaluating indirectness of populations, interventions and outcomes; these were assured in the network by limiting the included outcomes specific to adult with chronic migraine at week 12 and only for double blind phase of RCTs. Effect modifiers are balanced across the network. Thus, there is no evidence of intransitivity.
- **Publication Bias:** Non-statistical consideration of likelihood of non-publication of evidence, was assured through comprehensive searching in registered trials in the clinicaltrials.gov.

GRADE assessments indicated a relative certainty of evidence. The robust certainty degree was particularly highlighted for those estimations which drugs were compared with placebo.

Table 1: Summary of GRADE results for each outcomes

| **GRADE level**  **Outcomes** | **High** | **Moderate** | **Low** | **Very low** |
| --- | --- | --- | --- | --- |
| **MHDs** | * | * | * |  |
| **MMDs** | * | * |  |  |
| **MSQ-RR** | * | * |  |  |
| **MSQ-PR** | * |  | * |  |
| **MSQ-EF** | * | * |  |  |
| **HIT-6** | * | * |  |  |

**References**

1. Aurora S, Dodick D, Turkel C, et al. OnabotulinumtoxinA for treatment of chronic migraine: results from the double-blind, randomized, placebo-controlled phase of the PREEMPT 1 trial. *Cephalalgia* 2010;30(7):793-803.

2. Dodick DW, Silberstein SD, Lipton RB, et al. Early onset of effect of onabotulinumtoxinA for chronic migraine treatment: analysis of PREEMPT data. *Cephalalgia* 2019;39(8):945-56.

3. Dodick DW, Turkel CC, DeGryse RE, et al. OnabotulinumtoxinA for treatment of chronic migraine: Pooled results from the double‐blind, randomized, placebo‐controlled phases of the PREEMPT clinical program. *Headache: The Journal of Head and Face Pain* 2010;50(6):921-36.

4. Silberstein SD, Diener H-C, Dodick DW, et al. The impact of onabotulinumtoxinA vs. placebo on efficacy outcomes in headache day responder and nonresponder patients with chronic migraine. *Pain and Therapy* 2020;9(2):695-707.

5. Aurora S, Dodick D, Diener HC, et al. OnabotulinumtoxinA for chronic migraine: efficacy, safety, and tolerability in patients who received all five treatment cycles in the PREEMPT clinical program. *Acta Neurologica Scandinavica* 2014;129(1):61-70.

6. Lipton RB, Rosen NL, Ailani J, et al. OnabotulinumtoxinA improves quality of life and reduces impact of chronic migraine over one year of treatment: pooled results from the PREEMPT randomized clinical trial program. *Cephalalgia* 2016;36(9):899-908.

7. Detke HC, Goadsby PJ, Wang S, et al. Galcanezumab in chronic migraine: the randomized, double-blind, placebo-controlled REGAIN study. *Neurology* 2018;91(24):e2211-e21.

8. Ruff DD, Ford JH, Tockhorn-Heidenreich A, et al. Efficacy of galcanezumab in patients with chronic migraine and a history of preventive treatment failure. *Cephalalgia* 2019;39(8):931-44.

9. Ford J, Tassorelli C, Leroux E, et al. Changes in patient functioning and disability: results from a phase 3, double-blind, randomized, placebo-controlled clinical trial evaluating galcanezumab for chronic migraine prevention (REGAIN). *Quality of Life Research* 2021;30(1):105-15.

10. Förderreuther S, Zhang Q, Stauffer VL, et al. Preventive effects of galcanezumab in adult patients with episodic or chronic migraine are persistent: data from the phase 3, randomized, double-blind, placebo-controlled EVOLVE-1, EVOLVE-2, and REGAIN studies. *The journal of headache and pain* 2018;19(1):1-9.

11. Ailani J, Andrews JS, Rettiganti M, et al. Impact of galcanezumab on total pain burden: findings from phase 3 randomized, double-blind, placebo-controlled studies in patients with episodic or chronic migraine (EVOLVE-1, EVOLVE-2, and REGAIN trials). *The journal of headache and pain* 2020;21(1):1-9.

12. Ament M, Day K, Stauffer VL, et al. Effect of galcanezumab on severity and symptoms of migraine in phase 3 trials in patients with episodic or chronic migraine. *The journal of headache and pain* 2021;22(1):1-10.

13. Ailani J, Kuruppu DK, Rettiganti M, et al. Does “wearing off” of efficacy occur in galcanezumab‐treated patients at the end of the monthly treatment cycle? Post hoc analyses of four phase III randomized trials. *Headache: The Journal of Head and Face Pain* 2022;62(2):198-207.

14. Tobin JA, Joshi S, Ford JH, et al. Reductions in acute medication use and healthcare resource utilization in patients with chronic migraine: a secondary analysis of a phase 3, randomized, double-blind, placebo-controlled study of galcanezumab with open-label extension (REGAIN). *J Med Econ* 2022;25(1):1030-38.

15. Pozo-Rosich P, Dodick DW, Ettrup A, et al. Shift in diagnostic classification of migraine after initiation of preventive treatment with eptinezumab: post hoc analysis of the PROMISE studies. *BMC Neurol* 2022;22(1):1-10.

16. Diener H, Dodick D, Aurora S, et al. OnabotulinumtoxinA for treatment of chronic migraine: results from the double-blind, randomized, placebo-controlled phase of the PREEMPT 2 trial. *Cephalalgia* 2010;30(7):804-14.

17. Dodick DW, Lipton RB, Silberstein S, et al. Eptinezumab for prevention of chronic migraine: a randomized phase 2b clinical trial. *Cephalalgia* 2019;39(9):1075-85.

18. Ferrari MD, Diener HC, Ning X, et al. Fremanezumab versus placebo for migraine prevention in patients with documented failure to up to four migraine preventive medication classes (FOCUS): a randomised, double-blind, placebo-controlled, phase 3b trial. *The Lancet* 2019;394(10203):1030-40.

19. Spierings EL, Ning X, Ramirez Campos V, et al. Improvements in quality of life and work productivity with up to 6 months of fremanezumab treatment in patients with episodic and chronic migraine and documented inadequate response to 2 to 4 classes of migraine‐preventive medications in the phase 3b FOCUS study. *Headache: The Journal of Head and Face Pain* 2021;61(9):1376-86.

20. Ferrari MD, Zuurbier KW, Barash S, et al. Fremanezumab in individuals with chronic migraine who had inadequate response to onabotulinumtoxinA and topiramate or valproic acid. *Headache* 2022;62(4):530.

21. MaassenVanDenBrink A, Terwindt GM, Cohen JM, et al. Impact of age and sex on the efficacy of fremanezumab in patients with difficult-to-treat migraine: results of the randomized, placebo-controlled, phase 3b FOCUS study. *The journal of headache and pain* 2021;22(1):152.

22. Lipton RB, Goadsby PJ, Smith J, et al. Efficacy and safety of eptinezumab in patients with chronic migraine: PROMISE-2. *Neurology* 2020;94(13):e1365-e77.

23. Diener HC, Marmura MJ, Tepper SJ, et al. Efficacy, tolerability, and safety of eptinezumab in patients with a dual diagnosis of chronic migraine and medication‐overuse headache: subgroup analysis of PROMISE‐2. *Headache: The Journal of Head and Face Pain* 2021;61(1):125-36.

24. Silberstein S, Diamond M, Hindiyeh NA, et al. Eptinezumab for the prevention of chronic migraine: efficacy and safety through 24 weeks of treatment in the phase 3 PROMISE-2 (Prevention of migraine via intravenous ALD403 safety and efficacy–2) study. *The journal of headache and pain* 2020;21(1):1-12.

25. Buse DC, Winner PK, Charleston IV L, et al. Early response to eptinezumab indicates high likelihood of continued response in patients with chronic migraine. *The journal of headache and pain* 2022;23(1):29.

26. Cowan RP, Marmura MJ, Diener H-C, et al. Quantity changes in acute headache medication use among patients with chronic migraine treated with eptinezumab: subanalysis of the PROMISE-2 study. *The Journal of Headache and Pain* 2022;23(1):1-11.

27. Marmura MJ, Diener HC, Cowan RP, et al. Preventive migraine treatment with eptinezumab reduced acute headache medication and headache frequency to below diagnostic thresholds in patients with chronic migraine and medication‐overuse headache. *Headache: The Journal of Head and Face Pain* 2021;61(9):1421-31.

28. Lipton RB, Goadsby PJ, Dodick DW, et al. Evaluating the clinical utility of the patient‐identified most bothersome symptom measure from PROMISE‐2 for research in migraine prevention. *Headache: The Journal of Head and Face Pain* 2022;62(6):690-99.

29. Apelian R, Boyle L, Hirman J, et al. Measuring dose-related efficacy of eptinezumab for migraine prevention: post hoc analysis of PROMISE-1 and PROMISE-2. *The journal of headache and pain* 2022;23(1):1-12.

30. Ashina M, McAllister P, Cady R, et al. Efficacy and safety of eptinezumab in patients with migraine and self-reported aura: Post hoc analysis of PROMISE-1 and PROMISE-2. *Cephalalgia* 2022;42(8):696-704.

31. Martin V, Nagy AJ, Janelidze M, et al. Impact of baseline characteristics on the efficacy and safety of Eptinezumab in patients with migraine: subgroup analyses of PROMISE-1 and PROMISE-2. *Clinical Therapeutics* 2022;44(3):389-402.

32. Martin V, Tassorelli C, Ettrup A, et al. Eptinezumab for migraine prevention in patients 50 years or older. *Acta Neurologica Scandinavica* 2022;145(6):698-705.

33. McAllister P, Kudrow D, Cady R, et al. Reduction in migraine-associated burden after eptinezumab treatment in patients with chronic migraine. *Cephalalgia* 2022;42(10):1005-12.

34. Ashina M, Tepper SJ, Brandes JL, et al. Long‐term efficacy and safety of erenumab in patients with chronic migraine in whom prior preventive treatments had failed: A subgroup analysis. *Headache: The Journal of Head and Face Pain* 2022;62(5):624-33.

35. Starling AJ, Cowan RP, Buse DC, et al. Eptinezumab improved patient‐reported outcomes in patients with migraine and medication‐overuse headache: Subgroup analysis of the randomized PROMISE‐2 trial. *Headache: The Journal of Head and Face Pain* 2023;63(2):264-74.

36. Rothrock JF, Adams AM, Lipton RB, et al. FORWARD study: evaluating the comparative effectiveness of onabotulinumtoxinA and topiramate for headache prevention in adults with chronic migraine. *Headache: The Journal of Head and Face Pain* 2019;59(10):1700-13.

37. Blumenfeld AM, Patel AT, Turner IM, et al. Patient-reported outcomes from a 1-year, real-world, head-to-head comparison of onabotulinumtoxinA and topiramate for headache prevention in adults with chronic migraine. *Journal of Primary Care & Community Health* 2020;11:2150132720959936.

38. Sakai F, Suzuki N, Kim BK, et al. Efficacy and safety of fremanezumab for chronic migraine prevention: Multicenter, randomized, double‐blind, placebo‐controlled, parallel‐group trial in Japanese and Korean patients. *Headache: The Journal of Head and Face Pain* 2021;61(7):1092-101.

39. Takeshima T, Nakai M, Shibasaki Y, et al. Early onset of efficacy with fremanezumab in patients with episodic and chronic migraine: subanalysis of two phase 2b/3 trials in Japanese and Korean patients. *The Journal of Headache and Pain* 2022;23(1):1-9.

40. Saigoh K, Takeshima T, Nakai M, et al. Fremanezumab for Chronic Migraine Prevention in Japanese Patients: Subgroup Analysis from Two International Trials. *Journal of Pain Research* 2023:1311-19.

41. Silberstein SD, Lipton RB, Dodick DW, et al. Efficacy and safety of topiramate for the treatment of chronic migraine: A randomized, double‐blind, placebo‐controlled trial. *Headache: The Journal of Head and Face Pain* 2007;47(2):170-80.

42. Silberstein S, Lipton R, Dodick D, et al. Topiramate treatment of chronic migraine: A randomized, placebo‐controlled trial of quality of life and other efficacy measures. *Headache: The Journal of Head and Face Pain* 2009;49(8):1153-62.

43. Dodick DW, Silberstein S, Saper J, et al. The impact of topiramate on health‐related quality of life indicators in chronic migraine. *Headache: The Journal of Head and Face Pain* 2007;47(10):1398-408.

44. Silberstein SD, Dodick DW, Bigal ME, et al. Fremanezumab for the preventive treatment of chronic migraine. *New England Journal of Medicine* 2017;377(22):2113-22.

45. Winner PK, Spierings EL, Yeung PP, et al. Early onset of efficacy with fremanezumab for the preventive treatment of chronic migraine. *Headache: The Journal of Head and Face Pain* 2019;59(10):1743-52.

46. Lipton RB, Cohen JM, Gandhi SK, et al. Effect of fremanezumab on quality of life and productivity in patients with chronic migraine. *Neurology* 2020;95(7):e878-e88.

47. Silberstein SD, Cohen JM, Seminerio MJ, et al. The impact of fremanezumab on medication overuse in patients with chronic migraine: subgroup analysis of the HALO CM study. *The journal of headache and pain* 2020;21(1):1-10.

48. Blumenfeld AM, Stevanovic DM, Ortega M, et al. No “Wearing‐Off Effect” Seen in Quarterly or Monthly Dosing of Fremanezumab: Subanalysis of a Randomized Long‐Term Study. *Headache: The Journal of Head and Face Pain* 2020;60(10):2431-43.

49. Tepper S, Ashina M, Reuter U, et al. Safety and efficacy of erenumab for preventive treatment of chronic migraine: a randomised, double-blind, placebo-controlled phase 2 trial. *The Lancet Neurology* 2017;16(6):425-34.

50. Brandes JL, Diener H-C, Dolezil D, et al. The spectrum of response to erenumab in patients with chronic migraine and subgroup analysis of patients achieving≥ 50%,≥ 75%, and 100% response. *Cephalalgia* 2020;40(1):28-38.

51. Ashina M, Tepper S, Brandes JL, et al. Efficacy and safety of erenumab (AMG334) in chronic migraine patients with prior preventive treatment failure: a subgroup analysis of a randomized, double-blind, placebo-controlled study. *Cephalalgia* 2018;38(10):1611-21.

52. Tepper SJ, Diener H-C, Ashina M, et al. Erenumab in chronic migraine with medication overuse: subgroup analysis of a randomized trial. *Neurology* 2019;92(20):e2309-e20.

53. Lipton RB, Tepper SJ, Reuter U, et al. Erenumab in chronic migraine: patient-reported outcomes in a randomized double-blind study. *Neurology* 2019;92(19):e2250-e60.

54. Yu S, Kim B-K, Wang H, et al. A phase 3, randomised, placebo-controlled study of erenumab for the prevention of chronic migraine in patients from Asia: the DRAGON study. *The Journal of Headache and Pain* 2022;23(1):146.
